# Analytical Validation of an Assay for Concurrent Measurement of Amino Acids in Dog Serum and Comparison of Amino Acid Concentrations between Whole Blood, Plasma, and Serum from Dogs

**DOI:** 10.3390/metabo12100891

**Published:** 2022-09-22

**Authors:** Amanda B. Blake, Patricia E. Ishii, Robert K. Phillips, Jonathan A. Lidbury, Joerg M. Steiner, Jan S. Suchodolski

**Affiliations:** Gastrointestinal Laboratory, Department of Small Animal Clinical Sciences, Texas A&M University, College Station, TX 77843, USA

**Keywords:** aminogram, stability of amino acids, amino acid analysis, canine, gastrointestinal, deproteinization

## Abstract

Amino acids play an important role in metabolism. Comprehensive analytical validation of an assay for the concurrent measurement of a large number of amino acids in dogs is lacking, which precludes its usefulness in a clinical setting. Amino acids are often measured in plasma or whole blood. However, serum is commonly used for gastrointestinal diagnostic testing in dogs and is therefore convenient to use. This study aimed to analytically validate an assay for the concurrent measurement of amino acids in dog serum and to evaluate differences in amino acid concentrations in whole blood, plasma, and serum in dogs. Analytical validation of the assay (Biochrom 30+ Amino Acid Analyzer) was performed on fresh or banked serum samples from dogs. Whole blood, plasma, and serum from 36 healthy dogs were analyzed, and concentrations of the three sample types were compared. The assay was demonstrated to be precise, reproducible, accurate, linear, and stable for the measurement of the majority of compounds detected in dog serum. Cystine, glutamic acid, and ethanolamine were shown to be unstable at conditions commonly encountered in clinical settings. Significant differences in concentrations were identified between whole blood, plasma, and serum for 33 of 42 compounds. Amino acid profiles in serum and plasma were more similar to each other than to those in whole blood. While some amino acids are present in similar concentrations in whole blood, plasma, and serum, others are highly dependent on the type of biofluid, and measurements warrant strict adherence to sample type-based reference intervals.

## 1. Introduction

Amino acids (AA) are involved in the biosynthesis of functional molecules, the maintenance of tight junction proteins, the nourishment of enterocytes, and the regulation of metabolic pathways, oxidative stress, and inflammation of the gut [1]. Amino acids also have a direct relationship with the microbiota—bacteria in the gut that metabolize dietary amino acids and proteins into new metabolites that can then be absorbed by the host. For example, aromatic amino acid metabolites produced by the microbiota, such as indolepropionic acid, positively affect intestinal permeability and systemic immunity [2]. Another example is tryptamine, a bacterial metabolite of dietary tryptophan that has been shown to accelerate gastrointestinal (GI) transit by activating colonic secretion pathways in the host via epithelial G-protein-coupled receptors [3]. Therefore, amino acids have the potential to serve as non-invasive markers of GI health.

One significant hurdle to expanding our knowledge of amino acids in GI diseases in veterinary medicine is that amino acids are not commonly measured in veterinary medicine, and to the authors’ knowledge, only one commercial laboratory in the United States offers this service commercially. Plasma amino acids are routinely measured in humans, for example, in newborn infants to detect inborn errors of metabolism. Ion exchange chromatography (IEC) or liquid chromatography coupled to visible light or fluorescence-based detection have been used for more than 50 years for the detection and quantification of amino acids, and are still used today. New methods of detection and quantification involve mass spectrometry or nuclear magnetic resonance-based methods. However, the significantly increased cost to purchase, maintain, and operate these newer types of instruments can be a limiting factor to scalability in veterinary medicine. An extensive validation of IEC-based methods to measure amino acids in plasma or serum from dogs has not yet been published. Even though the majority of human studies utilize plasma as their preferred sample type (AA have been reported to be more stable in plasma than serum [4]), many clinical diagnostic tests for dog and cat gastrointestinal disease currently utilize serum [5,6]. Therefore, it may be more practical to measure AA in dog serum if possible.

The aims of this study were to analytically validate an assay for the concurrent measurement of a wide range of amino acids in dog serum and to measure and compare amino acid concentrations in whole blood, plasma, and serum from healthy dogs.

## 2. Materials and Methods

### 2.1. Sample Collection

This study was approved by the TAMU Institutional Animal Care and Use Committee (animal use protocol 2018-0112 CA and 2020-0184 CA). Excess dog serum from samples submitted to the Gastrointestinal Laboratory at Texas A&M University for routine diagnostic testing was used for analytical validation after all personal identifying features had been removed from the samples. For testing of short-term stability, serum was collected prospectively from 4 apparently healthy dogs recruited in October 2021. Additionally, healthy privately-owned pet dogs that had absence of clinical signs based on an owner questionnaire were recruited prospectively in October of 2018. Owners were requested to withhold food from their dog for 8–12 h prior to their appointment. Following recruitment, physical examination was performed, and blood, feces, and urine were collected to rule out clinically relevant abnormalities on complete blood count (CBC), serum biochemistry profile, urinalysis, canine fecal dysbiosis index [7], and gastrointestinal panel (serum cobalamin, folate, trypsin-like immunoreactivity, and pancreatic lipase immunoreactivity). Lithium heparin blood and whole clotted blood (without separator gel, allowed to clot at room temperature for 30 min) were centrifuged at 1800× *g* at 4 °C for 15 min to obtain plasma and serum, respectively. Multiple aliquots of each sample type were made for validation purposes. Within one hour of collection, aliquots of lithium heparin blood, serum, and plasma were stored at 4 °C and kept for 24 h to simulate overnight shipping conditions. Then, samples were deproteinized and transferred to −80 °C storage until further analysis.

### 2.2. Amino acid Analysis

Amino acids and other nitrogenous compounds were measured with a Biochrom 30+ lithium high performance amino acid analyzer (Biochrom Ltd., Cambridge, UK) following manufacturer’s guidelines. Biological samples were deproteinized following laboratory protocols (dx.doi.org/10.17504/protocols.io.877hzrn) [PROTOCOL DOI]. Briefly, samples were deproteinized with a 1:1 (*v*/*v*) addition of 5% sulfosalicylic acid + 500 µM L-norleucine as an internal standard and subsequently centrifuged at 10,000× *g* for 5 min at 4 °C. After centrifugation the precipitate was discarded. Whole blood required an additional transfer and centrifugation due to the loose nature of the precipitate. Up to 500 µL of supernatant was transferred to a 0.2 µm PVDF centrifugal filter tube and centrifuged at 10,000× *g* for 5 min at 4 °C. The supernatant was then placed on a chilled autosampler and injected into the instrument. Samples were not kept on the autosampler (4 °C) for longer than 48 h prior to being injected on the column. If needed, samples were stored at −80 °C prior to being placed on the autosampler. The instrument parameters were set to an injection volume of 30 µL with partial loop fill injection.

### 2.3. Validation

Two milliliters of excess serum were obtained from 8 different dogs and deproteinized according to the previously mentioned protocol. The resulting extract was distributed into 15 aliquots and stored at −80 °C. Eight aliquots from the same dog were run consecutively on the same day to test intra-assay variability (precision). The last injection from each dog was used together with the remaining aliquots to test inter-assay variability (reproducibility)—aliquots from each individual dog were run at least one day apart (and up to two weeks apart) 7 additional times.

Spiking recovery (accuracy/matrix effect) was tested by spiking excess serum from 5 dogs with pure standard (Amino acid standards, physiological, analytical standard, acidics/neutrals, basics, and L-glutamine, 2500 µM, Millipore Sigma, Burlington, MA, USA) to give a final added concentration of 100 µM, 200 µM, and 300 µM for the low, medium, and high spike, respectively (Table 1). Because this would increase the sample volume by more than 5%, the following equation was used to calculate spiking recovery percentage (*%R*):

(1) $$\%R=\frac{\left(spiked\;sample\;result-\left(unspiked\;sample\;result*DF\right)\right)*100\%}{known\;spike\;added\;concentration}$$

where $DF=\frac{sample\;volume}{total\;volume\;of\;sample\;plus\;spike}$.

Samples were also diluted with equivalent amounts of loading buffer to measure dilutional parallelism (linearity; Table 1). Dilutional parallelism was additionally tested with pure standards in standard diluent (lithium loading buffer, Biochrom Ltd., Cambridge, UK) due to the wide range of concentrations for the various amino acids in individual biological samples. Standard dilutions were made using amounts listed in Table S1.

To test stability of amino acids, excess serum from 8 dogs (collected the previous day and shipped on ice to the GI Lab at Texas A&M University) were distributed into 5 aliquots each and stored at −80 °C. The first aliquot was deproteinized immediately prior to storage, and the rest of the aliquots were thawed and deproteinized once a week for 4 weeks and returned to −80 °C storage. All samples were run as a batch at the end of the 4-week time period. After 61 weeks, the baseline aliquot was used to repeat amino acid analysis to test effects of long-term storage of deproteinized samples at −80 °C.

The second part of stability testing examined short-term storage conditions that clinical samples might be exposed to when being collected in a veterinary clinical setting. Fresh serum was collected from 4 dogs to test short-term stability. Blood samples were allowed to clot at room temperature for 30 min, then centrifuged at 1800× *g* at 4 °C for 15 min. Serum was transferred to a clean tube. Aliquots were made and tested under the listed conditions in Table 2.

Using a previously described objective photometric method [8], hemolysis scores (HS) were assigned to serum and plasma samples from healthy dogs to identify correlations between hemolysis and concentrations of amino acids.

To calculate the lower limit of quantitation (LLOQ), 5 replicates of low concentration standards (2.5 µM, 5 µM, 10 µM, and 15 µM) were analyzed for consistency (coefficient of variation; CV%) and signal to noise ratios (in 4 of the 5 replicates).

### 2.4. Statistical Analysis

A Shapiro–Wilk test for normality of the data was performed, and given non-normal distribution, Friedman test with Dunn’s *post hoc* multiple comparisons tests were performed to determine differences between amino acid concentrations over time in stability studies, as well as differences of AA concentrations between whole blood, plasma, and serum. Spearman’s correlations were performed to analyze correlations between individual amino acid concentrations in serum and plasma, serum biochemistry panel, CBC, clinical data (body condition score, fecal score, age), GI panel, and hemolysis score (HS). Spearman’s correlations were also performed to analyze correlations for each individual amino acid between the sample types (whole blood, plasma, and serum). These correlations were corrected for false discovery rate using Benjamini-Hochberg correction. All other validation testing was evaluated by calculating coefficients of variation (CV%) and observed to expected ratios (OE%). Data was analyzed using two statistical software programs (JMP Pro v14, SAS Institute Inc., Cary, NC, USA; Graph Pad Prism v 9.0.1, GraphPad Software, San Diego, CA, USA).

## 3. Results

### 3.1. Analytical Validation for Dog Serum

Signal to noise ratios above 3 were determined acceptable. Standard concentrations for which this was not achievable in the majority of 4 replicates were deemed below the lower limit of quantitation (LLOQ). Signal to noise ratios are available in Table S2. The determined LLOQ for the vast majority of compounds was 5 µM, with the exception of urea (30 µM), sarcosine (30 µM), β-aminoisobutyric acid (10 µM), γ-aminobutyric acid (10 µM), hydroxyproline (20 µM), and proline (10 µM). The cutoff LLOQ values have been multiplied by 2 to account for the dilution factor of 2 in serum samples.

### 3.2. Intra-Assay Variability (Precision)

A total of 31 compounds were detected with the majority of the eight samples’ median concentrations above the detection limit. The range of median CV%’s was 0.2–8.8 with only a few compounds with a CV% above 10% for some of the samples tested: phosphoserine, α-aminoadipic acid, cystathionine, 1-methylhistidine, and 3-methylhistidine (Table S3).

### 3.3. Inter-Assay Variability (Reproducibility)

A total of 32 compounds were detected with the majority of the samples above the detection limit. The range of median CV%’s was 0.7–21.5 with only a few compounds with a CV% above 10% for some of the samples tested: phosphoserine, α-aminoadipic acid, cystathionine, hydroxylysine, 1-methylhistidine, 3-methylhistidine, and carnosine (Table S4).

### 3.4. Spiking Recovery (Accuracy/Matrix Effect)

Results of spiking recovery are listed in Table S5. All recoveries for the following compounds fell within the acceptable range of 80–120%: phosphoethanolamine, aspartic acid, threonine, serine, asparagine, glutamic acid, glycine, citrulline, α-aminobutyric acid, valine, cystine, methionine, cystathionine, isoleucine, leucine, tyrosine, β-alanine, phenylalanine, β-aminoisobutyric acid, γ-aminobutyric acid, ethanolamine, ammonia, hydroxylysine, ornithine, lysine, 1-methylhistidine, histidine, tryptophan, 3-methylhistidine, anserine, carnosine, arginine, hydroxyproline, and proline. A few compounds had one or more recoveries fall outside of the acceptable range of 80–120%: phosphoserine (low spike only), taurine, urea, glutamine (low spike only), sarcosine (low spike only), α-aminoadipic acid (low spike only), alanine (low spike only), and homocysteine (low spike only).

### 3.5. Dilutional Parallelism (Linearity)

The results of sample dilution are listed in Table S6. A total of 24 compounds were detected in 3 or more of the 5 dogs tested: taurine, urea, threonine, serine, asparagine, glutamic acid, glutamine, glycine, alanine, citrulline, α-aminobutyric acid, valine, methionine, isoleucine, leucine, tyrosine, phenylalanine, ammonia, lysine, histidine, tryptophan, carnosine, arginine, and proline. The OE% for the majority of detected compounds fell within the accepted range (80–120%). Recovery was between 80–120% for all 5 dogs tested at all dilutions (down to 0.64 dilution factor) for 12 compounds: urea, threonine, serine, asparagine, glutamine, glycine, alanine, valine, lysine, histidine, arginine, and proline. When expected concentrations were below 26 µM for one or more sample, several compounds fell out of acceptable recovery range: glutamic acid, citrulline, α-aminobutyric acid, tyrosine, phenylalanine, ammonia, tryptophan, and carnosine. Methionine, isoleucine, and leucine had poor recovery at the strongest dilution (0.64 dilution factor) in one or two dogs tested. Taurine had poor recovery at all dilutions tested for one dog, and at 0.64 dilution factor for three dogs.

The results for linearity of standards are listed in Table S7. Several compounds were linear throughout the full range of standards (2.5–750 µM), including taurine, phosphoethanolamine, aspartic acid, threonine, serine, asparagine, glutamic acid, citrulline, valine, phenylalanine, β-aminoisobutyric acid, and arginine. Several more were linear down to 5 µM (α-aminobutyric acid, methionine, cystathionine, β-alanine, γ-aminobutyric acid, ornithine, and histidine) or 10 µM (phosphoserine, glutamine, alanine, tyrosine, homocystine, hydroxylysine, lysine, 1-methylhistidine, 3-methylhistidine, carnosine, hydroxyproline, and proline). Glycine, cystine, isoleucine, leucine, tryptophan, and anserine were linear down to 15 µM, α-aminoadipic acid and ethanolamine were linear down to 20 µM, ammonia was linear to 50 µM, urea was linear to 125 µM, and sarcosine was linear to 250 µM.

### 3.6. Stability

**Figure 1:**
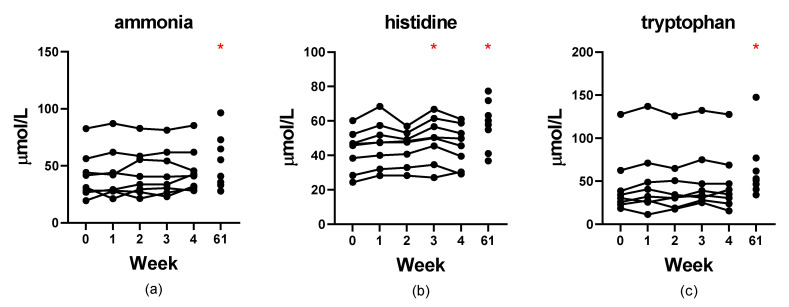
Stability of serum concentrations (µM) of ammonia (**a**), histidine (**b**), and tryptophan (**c**) in naïve serum stored at −80 °C for up to 4 weeks and deproteinized serum stored for 61 weeks. Red asterisks indicate significance (*p* < 0.05) compared to week 0.

The stability of serum amino acid concentrations for serum stored at −80 °C for up to 4 weeks and the stability of deproteinized serum samples stored at −80 °C for 61 weeks is presented File S1. The CV% for most compounds was acceptable (<10%) with the exception of a few compounds that were present in the samples at very low concentrations. The median CV% for 9 compounds was borderline acceptable (10–20%): aspartic acid, asparagine, glutamic acid, α-aminobutyric acid, isoleucine, tyrosine, ammonia, histidine, tryptophan. However, only 3 of these (ammonia, histidine, and tryptophan) also showed differences using Friedman’s and Dunn’s *post hoc* testing after storage for 61 weeks when compared to baseline (Figure 1). It is important to note that these results also include variability due to deproteinization since the samples were deproteinized separately one week apart.

**Figure 2:**
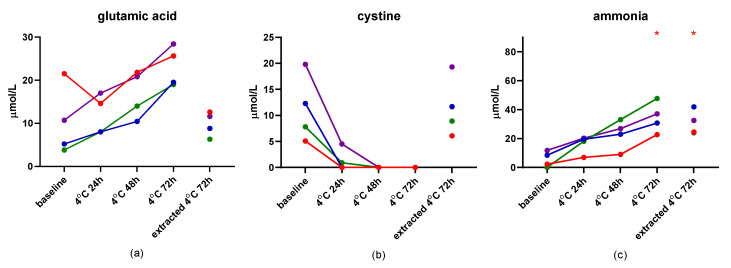
Stability of serum concentrations of glutamic acid (**a**), cystine (**b**), and ammonia (**c**) in naïve serum stored at 4 °C for up to 72 h and deproteinized serum stored for 72 h. Red asterisks indicate significance (*p* < 0.05) compared to baseline.

**Figure 3:**
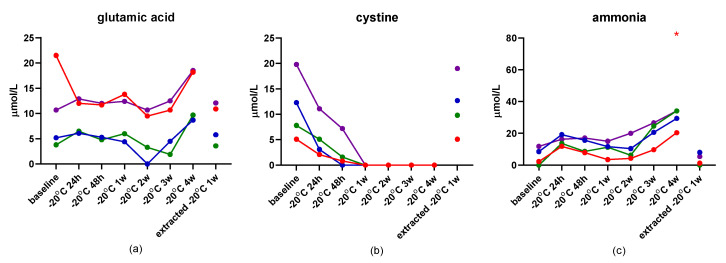
Stability of serum concentrations of glutamic acid (**a**), cystine (**b**), and ammonia (**c**) in naïve serum stored at −20 °C for up to 4 weeks and deproteinized serum stored for 1 week. Red asterisks indicate significance (*p* < 0.05) compared to baseline.

The results of the second short-term stability study are presented in Files S2 and S3. Storage of dog serum at 4 °C for up to 72 h yielded acceptable CV% (<10%), with the exception of those for the measurement of glutamic acid, cystine, ammonia, and hydroxyproline, with ammonia being the only one that also showed a difference with Dunn’s test at both 72 h time points (Figure 2). Storage of dog serum at −20 °C for up to 4 weeks showed the same amino acids as above with unacceptable CV%, but also isoleucine, ethanolamine, and carnosine. Ammonia was increased at 4 weeks of storage at −20 °C when compared to baseline (Figure 3).

### 3.7. Effect of Hemolysis

In serum, hemolysis (HS) positively correlated with phosphoserine, aspartic acid, and phosphoethanolamine, and negatively correlated with α-aminobutyric acid and phenylalanine. In plasma, HS positively correlated with aspartic acid and glutamic acid, and negatively correlated with α-aminobutyric acid and phenylalanine.

### 3.8. Other Correlations

In both serum and plasma, branched chain amino acids (BCAA; isoleucine, leucine and valine) positively correlated with white blood cell count. Additionally, in both serum and plasma, 3-methylhistidine showed a strong positive correlation with creatinine, and carnosine positively correlated with blood urea nitrogen. Glutamine positively correlated with age in both serum and plasma. Results of correlation analysis are presented in Table S8 for serum and in Table S9 for plasma.

### 3.9. Comparison of Results in Whole Blood, Plasma, and Serum

A total of 51 dogs were screened for inclusion. After biological sample testing, 15 dogs were excluded due to one or more clinically relevant abnormalities, leaving 36 dogs to be analyzed as a healthy group. Age of these dogs ranged from 0.6 to 13 years with a median age of 2.9 years. Of the dogs, 3 were intact females, 2 were intact males, 18 were spayed females, and 13 were neutered males. Eighteen dogs were mixed breed, four were Labrador Retrievers, two were miniature Dachshunds, and there were one of each of the following breeds: American Cocker Spaniel, Australian Shepherd, Catahoula, Fox Terrier, German Shepherd, German Shorthaired Pointer, Golden Retriever, Great Dane, Greyhound, Jack Russell Terrier, miniature Australian Shepherd, and Staffordshire Bull Terrier.

**Figure 4:**
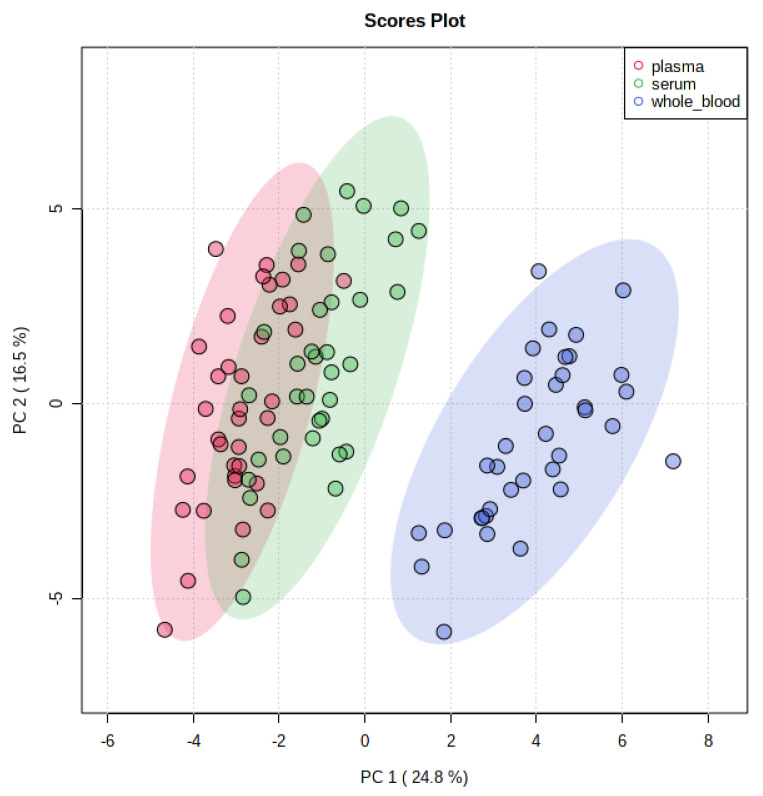
Principal component analysis scores 2D plot of amino acid profiles in whole blood, plasma, and serum. Plot was made using Metaboanalyst 5.0 free online software package and autoscaling of the concentration data.

At least one difference between whole blood, plasma, and serum concentrations was identified for 33 compounds (Table 3). No difference was identified for 6 compounds, and 3 compounds were not detected in any samples. Concentrations of taurine, phosphoethanolamine, aspartic acid, serine, glutamic acid, α-aminoadipic acid, citrulline, cystathionine, tyrosine, ammonia, ornithine, lysine, histidine, and arginine were significantly higher in whole blood than in plasma or serum. Concentrations of asparagine, glutamine, proline, glycine, valine, methionine, leucine, and phenylalanine were significantly higher in serum than in plasma or whole blood. Concentrations for 23 compounds were higher in serum than in plasma. Amino acid profiles of whole blood clustered separately from those of plasma and serum (Figure 4).

**Figure 5:**
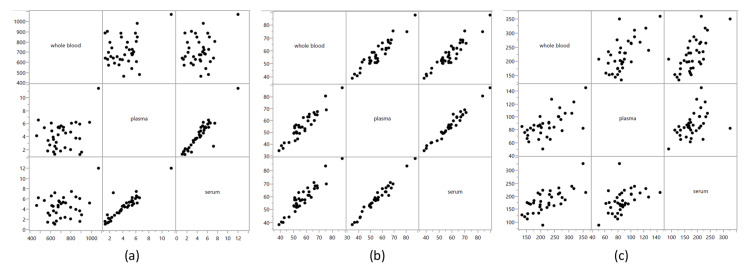
Spearman’s correlation of amino acid concentrations in whole blood, plasma, and serum from 36 healthy dogs. (**a**) α-aminoadipic acid, (**b**) isoleucine, (**c**) taurine. Axis values are in µM concentrations.

Correlations of amino acids between sample type varied. Of 117 comparisons, 19 showed a strong positive correlation (0.61 < ρ < 0.80; q < 0.001) and 70 showed a very strong positive correlation (0.81 < ρ < 1.00; q < 0.001). Figure 5 shows correlation graphs of representative amino acids, α-aminoadipic acid, isoleucine, and taurine. Concentrations of α-aminoadipic acid had a very strong positive correlation between plasma and serum (ρ = 0.873, q < 0.001), and neither of these were correlated with concentrations in whole blood (ρ = 0.0139, q = 0.431; and ρ = 0.133, q = 0.446; respectively). Isoleucine had very strong correlations in concentration between all three sample types (ρ > 0.894, q < 0.001 for the three comparisons). Taurine concentrations were only moderately positively correlated between whole blood and plasma (ρ = 0.559, q < 0.001) and plasma and serum (ρ = 0.524, q = 0.001) and strongly correlated between whole blood and serum (ρ = 0.648, q < 0.001). File S4 shows correlation graphs of all amino acids between sample type, and Table S10 shows the multiple comparison-adjusted *p*-values for these comparisons.

## 4. Discussion

There is growing interest in measuring amino acids in veterinary medicine. However, there are multiple sample types from which circulating free amino acids can be measured, including whole blood, plasma, and serum. Additionally, published reference intervals in dogs are only available for plasma (most amino acids) and whole blood (taurine only) [9]. This study provides a direct comparison of amino acid concentrations in all three biofluids in healthy dogs.

In summary, this study analytically validated a method to concurrently measure a wide variety of amino acids in dog serum using the Biochrom 30+ Amino Acid Analyzer. This study also showed that amino acid concentrations are generally different between whole blood, plasma, and serum (in 33 out of 39 detected compounds), and that serum contains higher concentrations of many amino acids when compared to plasma.

### 4.1. Analytical Validation of Dog Serum

This study demonstrated precision, reproducibility, accuracy, lack of apparent matrix effect, linearity, and stability for the measurement of amino acids in dog serum using the Biochrom 30+ Amino Acid Analyzer. Out of 42 total compounds that are part of the assay, 31 were consistently detected in the serum from dogs and had acceptable validation parameters (phosphoserine, taurine, urea, aspartic acid, threonine, serine, asparagine, glutamic acid, glutamine, glycine, alanine, citrulline, α-aminobutyric acid, valine, methionine, cystathionine, isoleucine, leucine, tyrosine, phenylalanine, ammonia, hydroxylysine, ornithine, lysine, 1-methylhistidine, histidine, tryptophan, 3-methylhistidine, carnosine, arginine, and proline). Compounds that had a CV% above 10% for some samples for intra- or inter-assay variability tended to have very low concentrations below or near the lower limit of quantitation.

The method performance was acceptable for the majority of amino acids measured with regard to lower limits of quantification. The LLOQ of our method ranged from 5 to 30 µM, corresponding to 75–900 pmol on-column injection. This is comparatively better sensitivity than NMR-based methods of amino acid quantification, which require nanomoles of amino acids [10]. Mass spectrometry-based methods have superior LLOQ for amino acids, being able to quantify them into single-digit picomole on-column injection amounts [11,12] and sometimes the low femtomoles amounts [13]. Amino acids that were consistently close to or below the detection limit in this study could potentially be measured with these other more sensitive methods. However, the sensitivity of the assay utilized in our study was sufficient for the majority of amino acids.

It was important that a thorough stability study be performed on dog serum, because it is known that interspecies differences in enzymatic activity in plasma can affect the stability of amino acids [14]. Cystine and ethanolamine were highly unstable in dog serum, and therefore, should not be reported for clinical samples using this assay. Previous studies have also reported the instability of cystine, and together with this study suggest that it can only be measured reliably if strict attention is paid to sample collection, storage, and deproteinization conditions with deproteinization occurring within one hour of sample collection [4,15,16]. Concentrations of free cystine rapidly decline in serum due to the formation of disulfide bonds to proteins [17]. Consequently, this protein-bound half-cystine is removed from the measurable sample in the deproteinization process. Treatment of the sample with a reducing agent will reverse the binding, however, this will only allow the measurement of total cysteine and not free cystine in the sample. In this study, serum ethanolamine concentrations were highly dependent on time since collection, regardless of storage time or temperature prior to deproteinization, showing a noticeable increase 1 week or more after collection. However, a previous study has shown ethanolamine to be stable in human serum at 4 °C and 22 °C for at least 24 h [18]. While unconfirmed, one possibility for the increases seen at longer storage times may be a reaction between ammonia in the serum and ethylene oxide commonly used to sterilize plastic laboratory vessels and tubes, that yields ethanolamine [19]. Additionally, phosphoethanolamine can decompose to ethanolamine and phosphate, leading to increases in ethanolamine concentrations with increasing storage time [20]. However, phosphoethanolamine concentrations were generally low in dog serum (<10 µM), so it is unlikely that this is a significant contributing factor.

While previous studies have stressed the importance of prompt deproteinization for accuracy [21,22], the results presented here suggest that deproteinization can be delayed for up to one month if samples are stored at −80 °C within one day of collection, without significantly affecting the majority of amino acid concentrations. Long-term storage (i.e., 61 weeks) at −80 °C, even with prompt deproteinization, may lead to slight increases in concentrations of ammonia, histidine, and tryptophan. Storage of dog serum at 4 °C for up to 72 h may affect glutamic acid, cystine, and ammonia. While no significant differences of concentrations were noted for glutamic acid or cystine for these storage conditions, this is likely due to a low sample number as glutamic acid concentrations more than doubled in 3 of 4 dogs from baseline to 72 h of storage at 4 °C and cystine concentrations dropped to zero for all 4 dogs by 48 h of storage at 4 °C. The source of the increase in serum glutamic acid concentrations is from the deamidation of glutamine to glutamic acid via enzymes that retain activity in serum until it is deproteinized. This deamidation reaction also releases ammonia, contributing to an increase in ammonia concentrations with increasing storage time. Ammonia was significantly increased after 72 h of storage at 4 °C and this finding is consistent with results from previous studies [23].

While some amino acid concentration changes were seen in serum stored at −20 °C for up to 4 weeks prior to deproteinization, these changes were relatively minor compared to the range of concentrations in healthy dogs, with the exception of those for cystine, ethanolamine, and ammonia. Similar to results from previous studies [24], once the serum was deproteinized, no further changes were observed in amino acid concentrations when stored at 4 °C for 72 h (with the exception of changes in ammonia concentrations) and −20 °C for 1 week. Hydroxyproline showed a relatively high coefficient of variation under all storage conditions. However, because assay variability data was not able to be collected for this compound, it is unclear whether this is due to biological variability or assay variability.

Interestingly, serum concentrations of 3-methylhistidine and carnosine were associated with serum concentrations of creatinine and blood urea nitrogen in healthy dogs, both common indices used for assessment of kidney function. 3-methylhistidine is used as an index of muscle turnover in humans, but has a reduced renal clearance in patients with kidney disease [16,25,26]. Symmetric dimethylarginine (SDMA) is another methylated amino acid that has recently gained recognition as an early marker for loss of kidney function in veterinary medicine [27]. Carnosine is predominantly present in muscle tissue, is metabolized in the kidneys in humans, and is an indicator of animal protein consumption [28,29]. This study suggests that 3-methylhistidine and carnosine have potential to be useful in evaluating kidney function, but further studies are needed to confirm this hypothesis.

Although hemolysis was found to correlate with the concentrations for several amino acids in plasma and serum, most were weak correlations. However, aspartic acid showed a moderate positive correlation with increased hemolysis in serum and plasma (q = 0.002, ρ = 0.410; q < 0.001, ρ = 0.490, respectively). This correlation was expected because aspartic acid is found in much higher concentrations in erythrocytes than in plasma or serum [30,31]. This suggests that aspartic acid concentrations should be interpreted with caution in hemolyzed samples. Further studies using spiked concentrations of hemolyzed red blood cells are necessary to determine the effect of hemolysis on amino acid quantification.

### 4.2. Whole Blood, Plasma, Serum

Typically, plasma is more commonly used for amino acid analysis. It is generally believed to be a more stable sample type [4]. To collect serum, blood needs to clot at room temperature, during which time enzymatic reactions can occur (conversion of asparagine to aspartic acid and glutamine to glutamic acid) as well as loss of free cystine due to disulfide binding to proteins [20,32]. However, one of the benefits to using serum is the ability to use plain red top tubes for collection, mitigating any variation from ratio of lithium heparin to sample. This study showed that the majority of amino acids measured using this method were stable in serum. It is difficult to compare results from different studies that use different sample types, but this study has provided results for whole blood, plasma, and serum in the same samples, which will allow general inferences to be made when comparing the sample types in the future.

Similar to results from previous studies, we found that concentrations of amino acids in serum were generally higher than in plasma [33,34]. This is due, in part, to the ex vivo release of amino acids from erythrocytes, leukocytes, and platelets during the clotting process. Additionally, three amino acids that had large differences in concentration between serum and plasma in dogs (taurine, arginine, and glutamic acid) also had large differences in concentration between serum and plasma in humans [33,34]. Concentrations of aspartic acid were highest in whole blood due to the majority of aspartic acid being contained within blood cells [30,31]. Our results suggest that specific reference intervals for plasma and serum (and whole blood) should not be used interchangeably.

The vast majority of amino acids (34/39) were strongly (0.61 < ρ < 0.80) to very strongly (0.81 < ρ < 1.00) positively correlated between plasma and serum measurements. Our results of taurine concentrations correlating between whole blood and plasma measurements resembles results shown by Ontiveros et al. in Golden Retriever dogs [35]. While this correlation may suggest that only one of the two sample types needs to be measured to determine taurine levels, the plasma taurine concentrations had a much narrower range compared to the whole blood measurements. In addition, previous studies have suggested that whole blood may be a better indicator of taurine due to differences in sample handling of plasma potentially affecting taurine levels [35]. Although this study did not aim to determine biological relevance of amino acids in each sample type, this information may help to do so in future studies.

Antibiotic administration can interfere with AA measurement with IEC, causing additional peaks that coelute with AAs of interest, increasing their apparent concentration [36]. Therefore, care was taken to ensure that the healthy dogs utilized for this study were not on any antibiotics or other medications other than monthly flea and heartworm preventatives. Additionally, food was withheld from all dogs for 8–12 h prior to blood collection because some amino acids, including BCAA have been shown to increase postprandially [37]. Dogs in this study were consuming a variety of commercially available maintenance diets. While previous studies have shown that diet can affect circulating levels of amino acids in dogs, our study population is representative of the clinical population of healthy dogs that would also be consuming a variety of diets [9,38].

Limitations of this study include the lack of comparison of different types of deproteinization agents. Deproteinization with sulfosalicylic acid may cause the loss of free tryptophan and it is thus preferred to use trichloroacetic acid for the determination of tryptophan [39]. However, in this study, it was of more interest to measure the amino acid profiles as a whole rather than optimizing the method for the measurement of individual amino acids. Additionally, while we did not examine the effect of freeze thaw cycles on AA stability in serum, previous studies have done so, showing that up to 3 freeze thaw cycles on deproteinized serum will have no effect on AA concentrations [40]. However, freeze thaw cycles have been shown to have an effect on serum that has not yet been deproteinized, significantly changing the concentrations of up to 15 different amino acids [18]. Another limitation of our study is the small sample size used for the short-term stability testing. While the results of this portion of the study may be considered preliminary, they do correspond well to what has been shown in the literature in regard to amino acid stability [21,22,23,24].

## 5. Conclusions

In conclusion, we analytically validated this assay for amino acid measurement in dog serum. For the most accurate measurement of serum amino acids, samples should be allowed to clot at room temperature for 15–30 min, centrifuged, and serum immediately frozen. Cystine and ethanolamine concentrations should not be reported under typical clinical collection conditions utilizing this method of sample preparation and analysis. Additionally, while amino acid profiles of dog serum and plasma are more similar to each other than whole blood, reference intervals for these sample types should not be used interchangeably.

## Figures and Tables

**Table 1:** The components used to make the spiked matrix samples and diluted matrix samples.

	Matrix Spike Level		
	Low	Medium	High
components	220 µL serum	190 µL serum	160 µL serum
	10 µL acidics/neutrals	20 µL acidics/neutrals	30 µL acidics/neutrals
	10 µL basics	20 µL basics	30 µL basics
	10 µL glutamine	20 µL glutamine	30 µL glutamine
	**Dilution Factor**		
	**0.88**	**0.76**	**0.64**
components	220 µL serum	190 µL serum	160 µL serum
	30 µL lithium loading buffer	60 µL lithium loading buffer	90 µL lithium loading buffer

Acidics/neutrals and basics physiological standards contain all amino acids noted in the assay at a concentration of 2500 µM with the exception of cystine at 1250 µM, in 0.1 N HCl. Glutamine was added separately after making solution of 2500 µM glutamine in lithium loading buffer (Biochrom Ltd., Cambridge, UK).

**Table 2:** Short-term stability study design.

Serum Volume (µL)	ID	Day 0—Collect	Day 1	Day 2	Day 3	Day 7	Day 14	Day 21	Day 28
600	A	deprot, run A_e_							
	B	store A_e_ 4 °C 72 h			run A_e_				
	C	store A_e_ −20 °C 1 w				run A_e_			
200	D	Aliquot, store 4 °C 24 h	deprot, run						
200	E	Aliquot, store 4 °C 48 h		deprot, run					
200	F	Aliquot, store 4 °C 72 h			deprot, run				
200	G	Aliquot, store −20 °C 24 h	deprot, run						
200	H	Aliquot, store −20 °C 48 h		deprot, run					
200	I	Aliquot, store −20 °C 1 w				deprot, run			
200	J	Aliquot, store −20 °C 2 w					deprot, run		
200	K	Aliquot, store −20 °C 3 w						deprot, run	
200	L	Aliquot, store −20 °C 4 w							deprot, run

deprot = deproteinize, run = analyze the sample on the amino acid analyzer, subscript e = sample deproteinized on day 0.

**Table 3:** Comparison of amino acid concentrations (µM) in whole blood, plasma, and serum of healthy dogs (*n* = 36).

	Whole Blood		Plasma		Serum		
	Interval	Median	Interval	Median	Interval	Median	*p*-Value
phospho-serine	7–13	9 ^a^	3–10	3 ^b^	4–14	9 ^a^	<0.001
taurine	135–359	208 ^a^	50–145	85 ^b^	89–326	175 ^c^	<0.001
phosphoethanolamine	5–14	10 ^a^	0–4	2 ^b^	0–10	4 ^c^	<0.001
urea	2656–7943	4867 ^a^	2961–8632	5109 ^b^	2937–8585	5149 ^b^	<0.001
aspartic acid	143–458	295 ^a^	4–10	6 ^b^	5–12	9 ^c^	<0.001
hydroxyproline	2–106	11	2–99	10	0–106	12	0.590
threonine	115–340	178 ^a^	94–346	173 ^b^	101–354	182 ^a^	<0.001
serine	84–176	120 ^a^	66–149	105 ^b^	69–170	112 ^c^	<0.001
asparagine	16–55	35 ^a^	31–88	61 ^b^	32–93	64 ^c^	<0.001
glutamic acid	40–80	58 ^a^	16–42	25 ^b^	24–45	34 ^c^	<0.001
glutamine	479–996	658 ^a^	490–1055	704 ^a^	494–1079	704 ^b^	<0.001
α-aminoadipic acid	465–1070	685 ^a^	1–11	4 ^b^	1–12	5 ^b^	<0.001
proline	81–256	134 ^a^	74–294	129 ^a^	80–312	135 ^b^	<0.001
glycine	139–362	204 ^a^	118–371	216 ^a^	128–390	222 ^b^	<0.001
alanine	241–570	364 ^a^	192–544	371 ^b^	207–553	367 ^a^	<0.001
citrulline	27–102	55 ^a^	20–86	48 ^b^	20–88	50 ^b^	<0.001
α-aminobutyric acid	9–48	23 ^a^	10–53	26 ^b^	11–53	27 ^b^	<0.001
valine	114–220	159 ^a^	101–215	157 ^a^	106–223	164 ^b^	<0.001
cystine	1–10	5 ^a^	2–17	7 ^b^	3–14	7 ^b^	<0.001
methionine	31–68	46 ^a^	34–73	46 ^a^	37–76	49 ^b^	<0.001
cystathionine	4–24	9 ^a^	2–14	6 ^b^	2–14	6 ^b^	<0.001
isoleucine	39–88	55 ^a^	35–88	54 ^b^	38–90	58 ^a^	<0.001
leucine	69–181	113 ^a^	68–189	117 ^a^	75–196	123 ^b^	<0.001
tyrosine	51–90	63 ^a^	24–73	43 ^b^	26–76	44 ^c^	<0.001
β-alanine	0–5	0	0–5	0	0–5	0	N/A
phenylalanine	39–76	52 ^a^	38–81	54 ^a^	40–86	56 ^b^	<0.001
homocystine	0–2	0	0–4	0	0–4	0	<0.001
ethanolamine	0–23	4	0–49	0	0–34	3	0.089
ammonia	62–117	83 ^a^	37–81	51 ^b^	55–104	67 ^c^	<0.001
hydroxylysine	10–17	13	9–17	14	10–17	14	0.423
ornithine	13–35	21 ^a^	6–23	12 ^b^	7–24	12 ^b^	<0.001
lysine	157–541	272 ^a^	57–288	147 ^b^	64–305	154 ^c^	<0.001
1-methylhistidine	4–38	7 ^a^	4–31	8 ^b^	5–33	8 ^b^	<0.001
histidine	59–96	79 ^a^	50–84	70 ^b^	52–88	74 ^c^	<0.001
tryptophan	18–52	29 ^a^	31–110	55 ^b^	30–114	58 ^c^	<0.001
3-methylhistidine	4–19	8 ^a^	3–22	9 ^b^	4–22	10 ^b^	<0.001
anserine	0–10	3	2–19	3	0–16	3	0.144
carnosine	11–25	16 ^a^	11–45	25 ^b^	11–42	26 ^b^	<0.001
arginine	121–242	182 ^a^	62–157	109 ^b^	96–197	147 ^c^	<0.001

Values listed are in µM. Interval represents the middle 95% of the data. Values not sharing a common letter are significantly different (*p* < 0.05) according to Friedman testing with Dunn’s multiple comparison tests. Sarcosine, beta-aminoisobutyric acid, and gamma-aminobutyric acid were not detected in any samples and are therefore not listed in the table.

## Data Availability

The data presented in this study are available in the article and supplementary material.

## Supplementary Materials

The following supporting information can be downloaded at: https://www.mdpi.com/article/10.3390/metabo12100891/s1, Table S1: Standard dilution table. Preparation of standards ranging in concentration from 2.5 to 750 µM.; Table S2: Signal to noise ratios for 4 replicates of low concentration standards. Signal to noise ratios above 3 were determined acceptable.; Table S3: Intra-assay variability (precision). Concentrations and coefficient of variation for eight replicates run consecutively from each of eight dogs.; Table S4: Inter-assay variability (reproducibility). Concentrations and coefficient of variation for eight replicates run 1 to 14 days apart from each of eight dogs.; Table S5: Spiking recovery. Concentrations (µM) and percent recovery (%R) are listed for each of 5 dogs for low (100 µM), medium (200 µM), and high (300 µM) spike conditions.; Table S6: Sample matrix dilution (linearity). Percent recovery is listed for each of 5 dogs for three dilutions. The following amino acids were not detected at any dilution in any sample and therefore graphs were excluded: phosphoserine, phosphoethanolamine, α-aminoadipic acid, cystathionine, β-alanine, β-aminoisobutyric acid, homocystine, γ-aminobutyric acid, ethanolamine, hydroxylysine, and anserine. DF, dilution factor; Obs, observed; Exp, expected; % R, percent recovery.; Table S7: Linearity of standards. Cells with a tan color under expected concentrations columns indicate typical range of expected concentrations in healthy dog serum. Exp, expected; Obs, observed; OE%, observed to expected ratio.; Table S8: Spearman’s correlation of serum amino acids. This table shows spearman’s rank and q-values for each correlation of individual amino acid concentrations in serum to serum biochemistry panel (blue), complete blood count (orange), clinical data (body condition score, BCS; fecal score; age of the dog; green), GI panel (purple), and hemolysis score, HS (red).; Table S9: Spearman’s correlation of plasma amino acids. This table shows spearman’s rank and q-values for each correlation of individual amino acid concentrations in plasma to serum biochemistry panel (blue), complete blood count (orange), clinical data (body condition score, BCS; fecal score; age of the dog; green), GI panel (purple), and hemolysis score, HS (red).; Table S10: Spearman’s correlation of amino acids between sample types. This table shows spearman’s rank and q-values for each correlation of individual amino acid concentrations between whole blood, plasma, and serum of 36 healthy dogs. File S1: Stability of amino acids in dog serum stored at −80 °C and their CV%. Red asterisks on graphs indicate significance (*p* < 0.05) compared to week 0 with Friedman testing and Dunn’s multiple comparisons test.; File S2: Stability of amino acids in dog serum stored at 4 °C and their CV%. Red asterisks on graphs indicate significance (*p* < 0.05) compared to baseline with Friedman testing and Dunn’s multiple comparisons test.; File S3: Stability of amino acids in dog serum stored at −20 °C and their CV%. Red asterisks on graphs indicate significance (*p* < 0.05) compared to baseline with Friedman testing and Dunn’s multiple comparisons test. File S4: Spearman’s correlation of amino acids between sample types. These graphs depict visually amino acid concentrations comparisons between whole blood, plasma, and serum of 36 healthy dogs. Although *p*-values here are not multiplicity-adjusted, the level of significance did not change after multiple comparisons adjustments were made.

## References

[1] Liu Y., Wang X., Hu C.-A.A. (2017). Therapeutic Potential of Amino Acids in Inflammatory Bowel Disease. Nutrients.

[2] Dodd D., Spitzer M.H., Van Treuren W., Merrill B.D., Hryckowian A.J., Higginbottom S.K., Le A., Cowan T.M., Nolan G.P., Fischbach M.A. (2017). A Gut Bacterial Pathway Metabolizes Aromatic Amino Acids into Nine Circulating Metabolites. Nature.

[3] Bhattarai Y., Williams B.B., Battaglioli E.J., Whitaker W.R., Till L., Grover M., Linden D.R., Akiba Y., Kandimalla K.K., Zachos N.C. (2018). Gut Microbiota-Produced Tryptamine Activates an Epithelial G-Protein-Coupled Receptor to Increase Colonic Secretion. Cell Host Microbe.

[4] Dewolfe M.S., Baskurt S., Cochrane W.A. (1967). Automatic Amino Acid Analysis of Blood Serum and Plasma. Clin. Biochem..

[5] Dossin O. (2011). Laboratory Tests for Diagnosis of Gastrointestinal and Pancreatic Diseases. Top. Companion Anim. Med..

[6] Allenspach K. (2015). Diagnosis of Small Intestinal Disorders in Dogs and Cats. Clin. Lab. Med..

[7] AlShawaqfeh M.K., Wajid B., Minamoto Y., Markel M., Lidbury J.A., Steiner J.M., Serpedin E., Suchodolski J.S. (2017). A Dysbiosis Index to Assess Microbial Changes in Fecal Samples of Dogs with Chronic Inflammatory Enteropathy. FEMS Microbiol. Ecol..

[8] Appierto V., Callari M., Cavadini E., Morelli D., Daidone M.G., Tiberio P. (2014). A Lipemia-Independent Nanodrop((R))-Based Score to Identify Hemolysis in Plasma and Serum Samples. Bioanalysis.

[9] Delaney S.J., Kass P.H., Rogers Q.R., Fascetti A.J. (2003). Plasma and Whole Blood Taurine in Normal Dogs of Varying Size Fed Commercially Prepared Food. J. Anim. Physiol. Anim. Nutr..

[10] Sakamoto T., Qiu Z., Inagaki M., Fujimoto K. (2020). Simultaneous Amino Acid Analysis Based on (19)F Nmr Using a Modified Opa-Derivatization Method. Anal. Chem..

[11] Kaspar H., Dettmer K., Gronwald W., Oefner P.J. (2008). Automated Gc–Ms Analysis of Free Amino Acids in Biological Fluids. J. Chromatogr. B.

[12] Ayon N.J., Sharma A.D., Gutheil W.G. (2019). Lc-Ms/Ms-Based Separation and Quantification of Marfey’s Reagent Derivatized Proteinogenic Amino Acid Dl-Stereoisomers. J. Am. Soc. Mass Spectrom..

[13] Le A., Ng A., Kwan T., Cusmano-Ozog K., Cowan T.M. (2014). A Rapid, Sensitive Method for Quantitative Analysis of Underivatized Amino Acids by Liquid Chromatography-Tandem Mass Spectrometry (Lc-Ms/Ms). J. Chromatogr. B Anal. Technol. Biomed. Life Sci..

[14] Van Eijk H.M., Dejong C.H., Deutz N.E., Soeters P.B. (1994). Influence of Storage Conditions on Normal Plasma Amino-Acid Concentrations. Clin. Nutr..

[15] Takehana S., Yoshida H., Ozawa S., Yamazaki J., Shimbo K., Nakayama A., Mizukoshi T., Miyano H. (2016). The Effects of Pre-Analysis Sample Handling on Human Plasma Amino Acid Concentrations. Clin. Chim. Acta.

[16] Hansen B., DiBartola S.P., Chew D.J., Brownie C., Berrie H.K. (1992). Amino Acid Profiles in Dogs with Chronic Renal Failure Fed Two Diets. Am. J. Vet. Res..

[17] Downes A.M. (1961). The Fate of Intravenous Doses of Free and Plasma Protein-Bound [^35^s]Cystine in the Sheep. Aust. J. Biol. Sci..

[18] An Z., Shi C., Li P., Liu L. (2021). Stability of Amino Acids and Related Amines in Human Serum under Different Preprocessing and Pre-Storage Conditions Based on Itraq^®^-Lc-Ms/Ms. Biol. Open.

[19] Yu B.L., Han J., Hammond M., Wang X., Zhang Q., Clausen A., Forster R., Eu M. (2017). Kinetic Modeling of the Release of Ethylene Oxide from Sterilized Plastic Containers and Its Interaction with Monoclonal Antibodies. PDA J. Pharm. Sci. Technol..

[20] Holtrop S., Abeling N.G.G., van Gennip A.H. Recommendations to Improve the Quality of Diagnostic Quantitative Analysis of Amino Acids in Plasma and Urine Using Cation-Exchange Liquid Chromatography with Post Column Ninhydrin Reaction and Detection. *ERNDIM*
**2002**. https://www.yumpu.com/en/document/read/19477578/amino-acid-analysis-recommendations-erndim.

[21] Schaefer A., Piquard F., Haberey P. (1987). Plasma Amino-Acids Analysis: Effects of Delayed Samples Preparation and of Storage. Clin. Chim. Acta.

[22] Li J., Piao C., Jin H., Wongpanit K., Manabe N. (2009). Delayed Deproteinization Causes Methodological Errors in Amino Acid Levels in Plasma Stored at Room Temperature or −20 °C. Asian-Aust. J. Anim. Sci..

[23] Hester J.R., Korzun W.J., Mabry L.U. (2015). Blood Ammonia Stability Revisited. Clin. Lab. Sci..

[24] Olek K., Uhlhaas S., Wardenbach P., Yamaguchi M. (1979). Influence of Storing Conditions on the Amino Acid Concentration in Human Serum (Author’s Transl). J. Clin. Chem. Clin. Biochem..

[25] Kochlik B., Gerbracht C., Grune T., Weber D. (2018). The Influence of Dietary Habits and Meat Consumption on Plasma 3-Methylhistidine—A Potential Marker for Muscle Protein Turnover. Mol. Nutr. Food Res..

[26] Young V.R., Munro H.N. (1978). Ntau-Methylhistidine (3-Methylhistidine) and Muscle Protein Turnover: An Overview. Fed. Proc..

[27] Jepson R.E., Syme H.M., Vallance C., Elliott J. (2008). Plasma Asymmetric Dimethylarginine, Symmetric Dimethylarginine, L-Arginine, and Nitrite/Nitrate Concentrations in Cats with Chronic Kidney Disease and Hypertension. J. Vet. Intern. Med..

[28] Peters V., Klessens C.Q.F., Baelde H.J., Singler B., Veraar K.A.M., Zutinic A., Drozak J., Zschocke J., Schmitt C.P., de Heer E. (2015). Intrinsic Carnosine Metabolism in the Human Kidney. Amino Acids.

[29] Mitry P., Wawro N., Rohrmann S., Giesbertz P., Daniel H., Linseisen J. (2019). Plasma Concentrations of Anserine, Carnosine and Pi-Methylhistidine as Biomarkers of Habitual Meat Consumption. Eur. J. Clin. Nutr..

[30] Seip M., Lindemann R., Gjesdahl P., Gjessing L.R. (1975). Amino Acid Concentrations in Plasma and Erythrocytes in Aregeneratory and Haemolytic Anaemias. Scand. J. Haematol..

[31] Fukuda K., Nishi Y., Usui T. (1984). Free Amino Acid Concentrations in Plasma, Erythrocytes, Granulocytes, and Lymphocytes in Umbilical Cord Blood, Children, and Adults. J. Pediatr. Gastroenterol. Nutr..

[32] Perry T.L., Hansen S. (1969). Technical Pitfalls Leading to Errors in the Quantitation of Plasma Amino Acids. Clin. Chim. Acta.

[33] Davids M., Peters J.H., de Jong S., Teerlink T. (2013). Measurement of Nitric Oxide-Related Amino Acids in Serum and Plasma: Effects of Blood Clotting and Type of Anticoagulant. Clin. Chim. Acta.

[34] Sotelo-Orozco J., Chen S.-Y., Hertz-Picciotto I., Slupsky C.M. (2021). A Comparison of Serum and Plasma Blood Collection Tubes for the Integration of Epidemiological and Metabolomics Data. Front. Mol. Biosci..

[35] Ontiveros E.S., Whelchel B.D., Yu J., Kaplan J.L., Sharpe A.N., Fousse S.L., Crofton A.E., Fascetti A.J., Stern J.A. (2020). Development of Plasma and Whole Blood Taurine Reference Ranges and Identification of Dietary Features Associated with Taurine Deficiency and Dilated Cardiomyopathy in Golden Retrievers: A Prospective, Observational Study. PLoS ONE.

[36] Williams A.P. (1986). General Problems Associated with the Analysis of Amino Acids by Automated Ion-Exchange Chromatography. J. Chromatogr. A.

[37] Holecek M. (2018). Branched-Chain Amino Acids in Health and Disease: Metabolism, Alterations in Blood Plasma, and as Supplements. Nutr. Metab..

[38] Donadelli R.A., Pezzali J.G., Oba P.M., Swanson K.S., Coon C., Varney J., Pendlebury C., Shoveller A.K. (2020). A Commercial Grain-Free Diet Does Not Decrease Plasma Amino Acids and Taurine Status but Increases Bile Acid Excretion When Fed to Labrador Retrievers. Transl. Anim. Sci..

[39] Parvy P., Bardet J., Gasquet M., Rabier D., Kamoun P. (1995). Stability of Free Amino Acids in Sulfosalicylic Filtrates. Clin. Chem..

[40] Kornhuber M.E., Balabanova S., Heiligensetzer G.V., Kornhuber C., Zettlmeissl H., Kornhuber A.W. (1991). Stability of Human Blood Serum Aminoacids after Storage at Different Ph and Temperature Conditions. Clin. Chim. Acta.

