# Supplementary material for: Analytical Validation of an Assay for Concurrent Measurement of Amino Acids in Dog Serum and Comparison of Amino Acid Concentrations between Whole Blood, Plasma, and Serum from Dogs
Source: Metabolites. 2022 Sep 22;12(10):891. doi: 10.3390/metabo12100891 (PMC9608751; doi:10.3390/metabo12100891)
Supplement: Supplementary file 1 [file metabolites-12-00891-s001.zip › File S1.pdf]

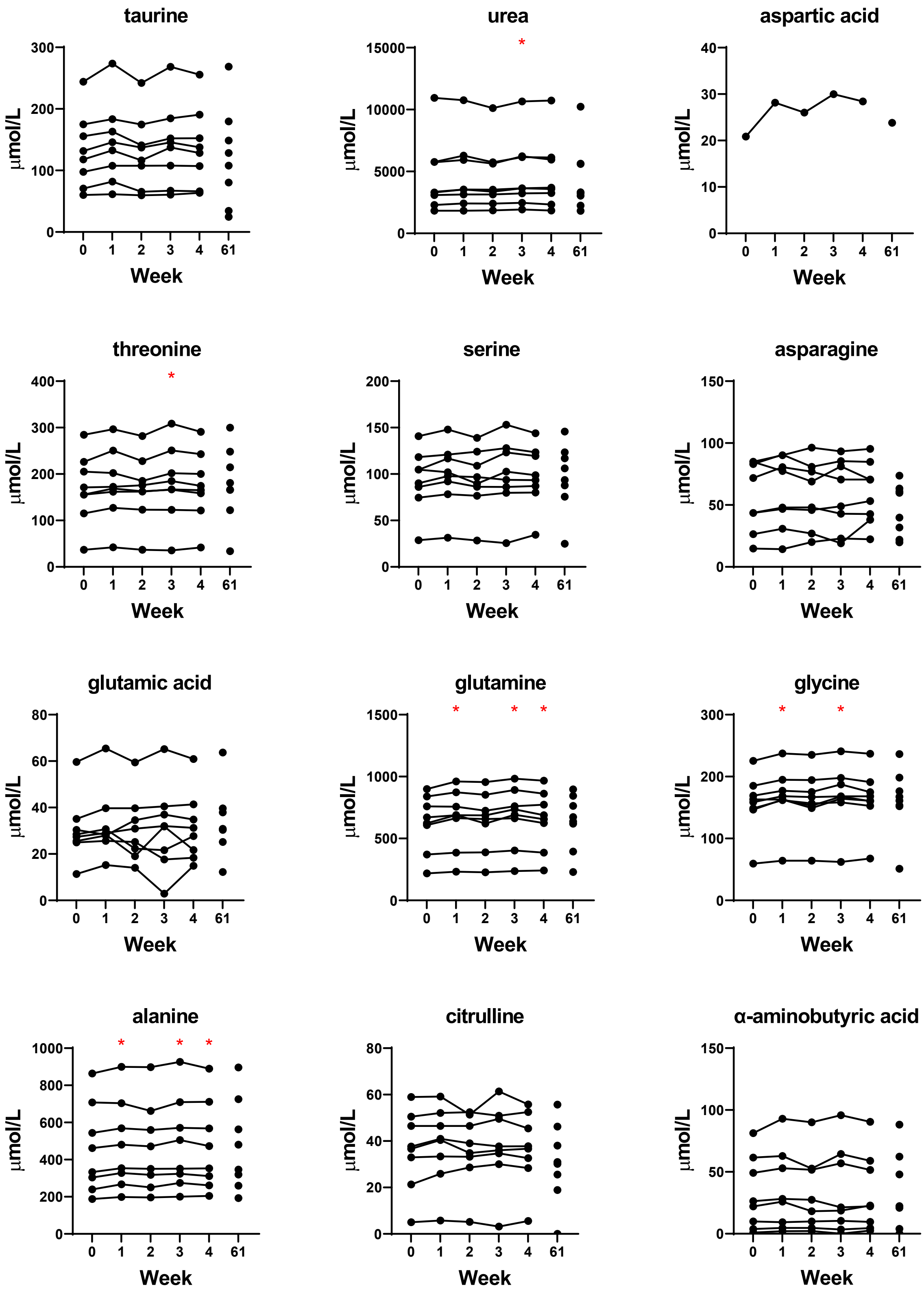
taurine
urea
aspartic acid
threonine
serine
asparagine
glutamic acid
glutamine
glycine
alanine
citrulline
α-aminobutyric acid
µmol/L
Week
0 1 2 3 4 61
*

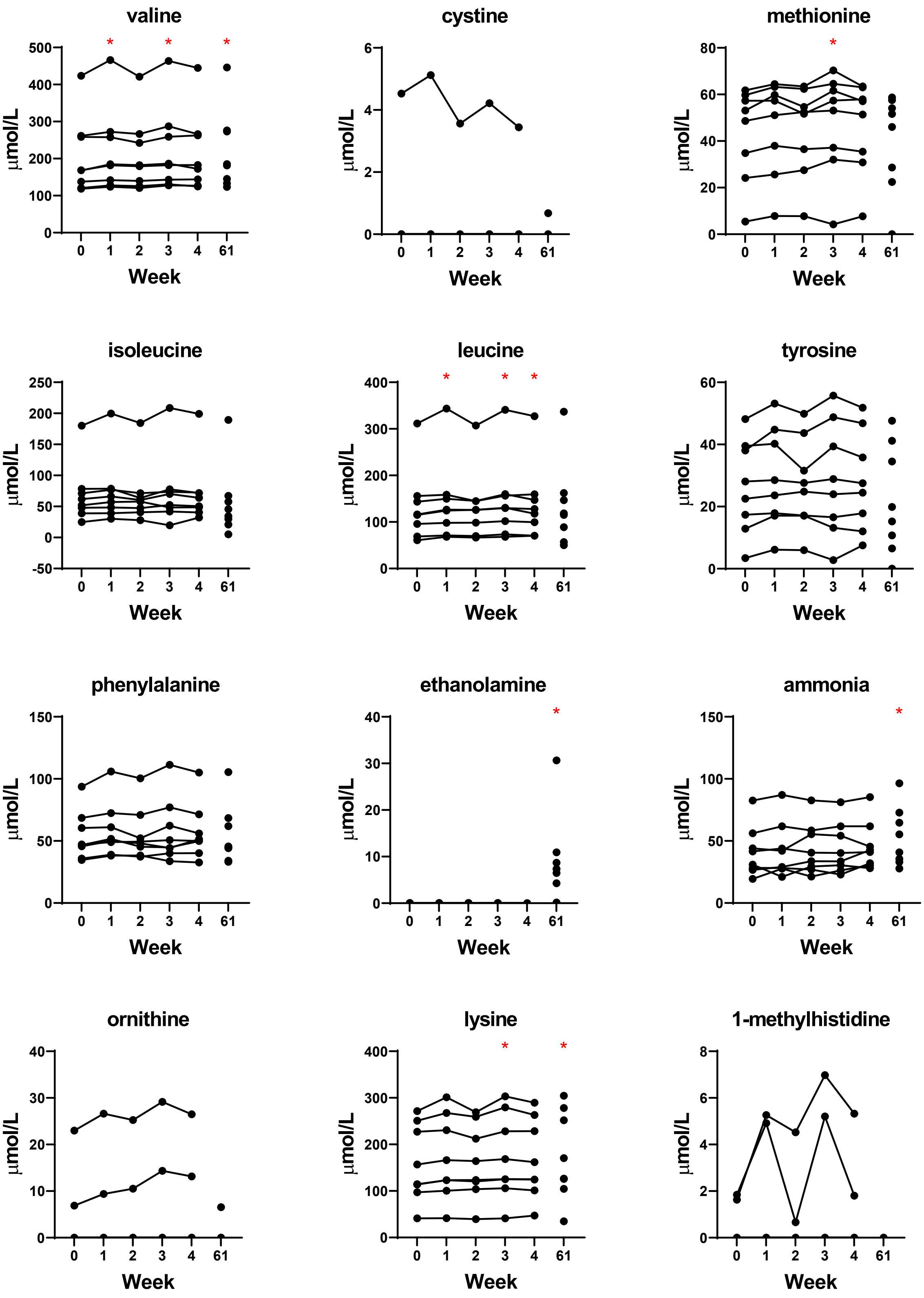
valine
cystine
methionine
isoleucine
leucine
tyrosine
phenylalanine
ethanolamine
ammonia
ornithine
lysine
1-methylhistidine
µmol/L
Week
0 1 2 3 4 61

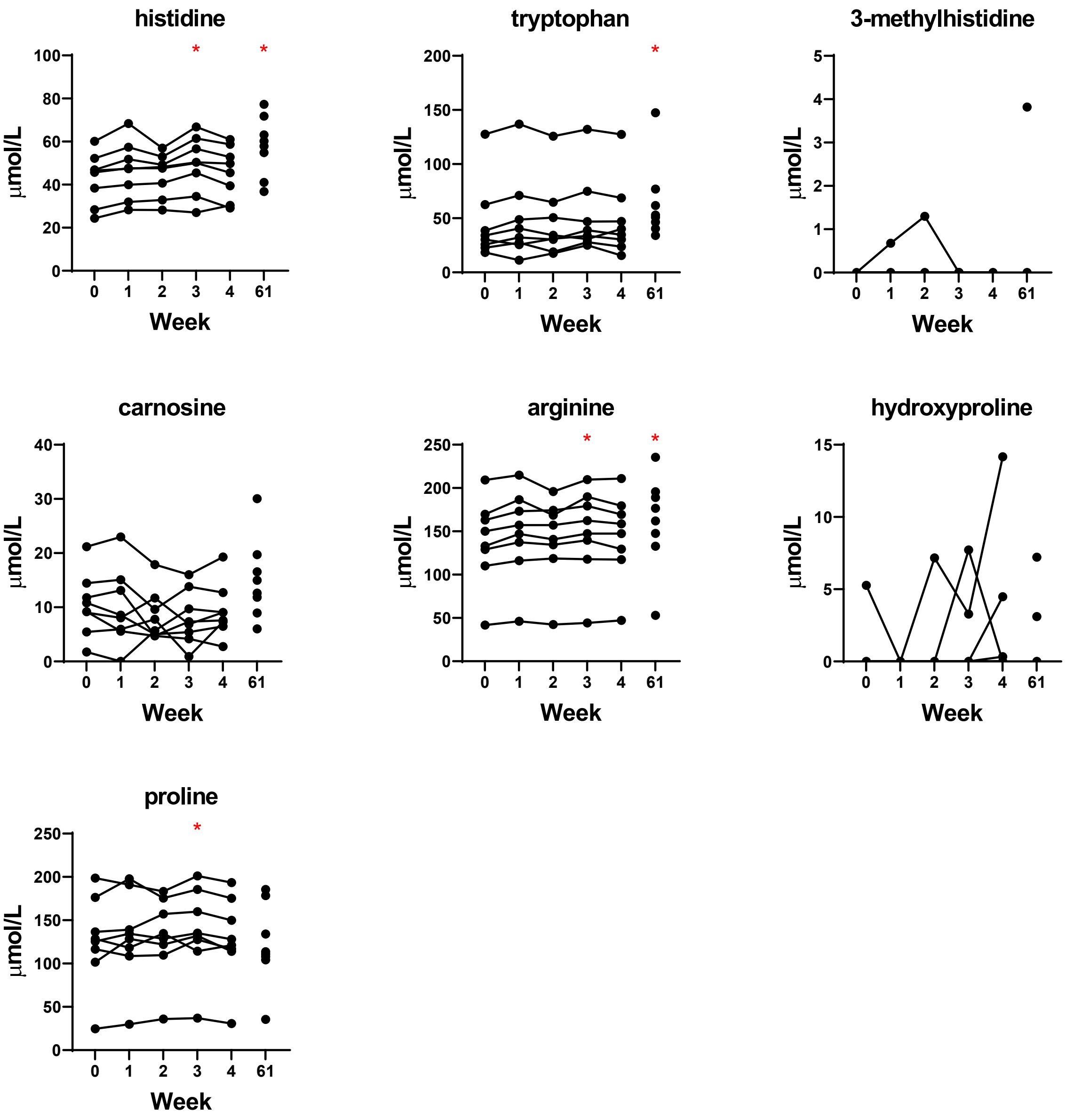

Stability of amino acids in dog serum stored at −80°C. Red asterisks indicate significance ($p<0.05$) in Dunn's *post hoc* testing when compared to week 0.

Stability of amino acids in dog serum stored at −80°C and their coefficients of variation (CV%).

Compound	median [range] µM[a]	median [range] CV%[b]
taurine	130 [60-262]	6.5 [3.1-24.8]
urea	3495 [1840-10693]	3.5 [1.9-4.2]
aspartic acid	0 [0-27]	11.8 [11.8-11.8]
threonine	170 [37-294]	3.0 [2.3-8.2]
serine	98 [29-145]	3.0 [2.4-11.4]
asparagine	59 [20-92]	10.8 [8.8-22.6]
glutamic acid	30 [13-62]	13.8 [4.0-35.7]
glutamine	667 [230-959]	3.2 [2.0-4.5]
glycine	165 [63-237]	3.0 [2.0-8.5]
alanine	414 [198-897]	2.6 [1.6-4.4]
citrulline	37 [5-57]	8.4 [4.3-49.2]
α-aminobutyric acid	23 [2-90]	11.7 [5.0-79.9]
valine	183 [124-445]	3.1 [1.8-4.0]
cystine	0 [0-4]	39.6 [39.6-39.6]
methionine	54 [7-63]	5.9 [3.9-51.1]
isoleucine	57 [26-194]	14.1 [5.1-38.7]
leucine	124 [67-332]	4.2 [3.5-10.7]
tyrosine	26 [5-51]	13.2 [5.5-58.5]
phenylalanine	48 [34-105]	5.8 [3.6-6.5]
ethanolamine	0 [0-0]	223.6 [223.6-223.6]
ammonia	38 [27-84]	12.6 [6.0-20.3]
ornithine	0 [0-26]	42.6 [32.9-52.2]
lysine	145 [41-295]	3.6 [2.8-9.0]
1-methylhistidine	0 [0-5]	68.1 [56.3-79.9]
histidine	48 [28-64]	10.7 [8.6-13.3]
tryptophan	35 [18-130]	18.3 [5.6-36.3]
3-methylhistidine	0 [0-0.3]	141.1 [141.1-141.1]
carnosine	8 [5-20]	34.4 [21.2-64.5]
arginine	153 [45-210]	5.5 [4.6-8.2]
hydroxyproline	0 [0-5]	223.6 [93.0-223.6]
proline	124 [33-192]	6.3 [3.4-13.3]

Stability at −80°C. Amino acids excluded from the table (not detected in all samples): phosphoserine, phosphoethanolamine, sarcosine, α-aminoadipic acid, cystathionine, β-alanine, β-aminoisobutyric acid, homocystine, γ-aminobutyric acid, hydroxylysine, anserine. [a]Concentrations of samples used (the median and range of the median of concentrations from eight dogs across six different timepoints of storage at −80°C). [b]Coefficient of variation, calculated from the same six timepoints: baseline, storage at −80°C for 1, 2 3, and 4 weeks prior to deproteinization, and storage at −80°C for 61 weeks following deproteinization.
