# Supplementary material for: Analytical Validation of an Assay for Concurrent Measurement of Amino Acids in Dog Serum and Comparison of Amino Acid Concentrations between Whole Blood, Plasma, and Serum from Dogs
Source: Metabolites. 2022 Sep 22;12(10):891. doi: 10.3390/metabo12100891 (PMC9608751; doi:10.3390/metabo12100891)
Supplement: Supplementary file 1 [file metabolites-12-00891-s001.zip › File S2.pdf]

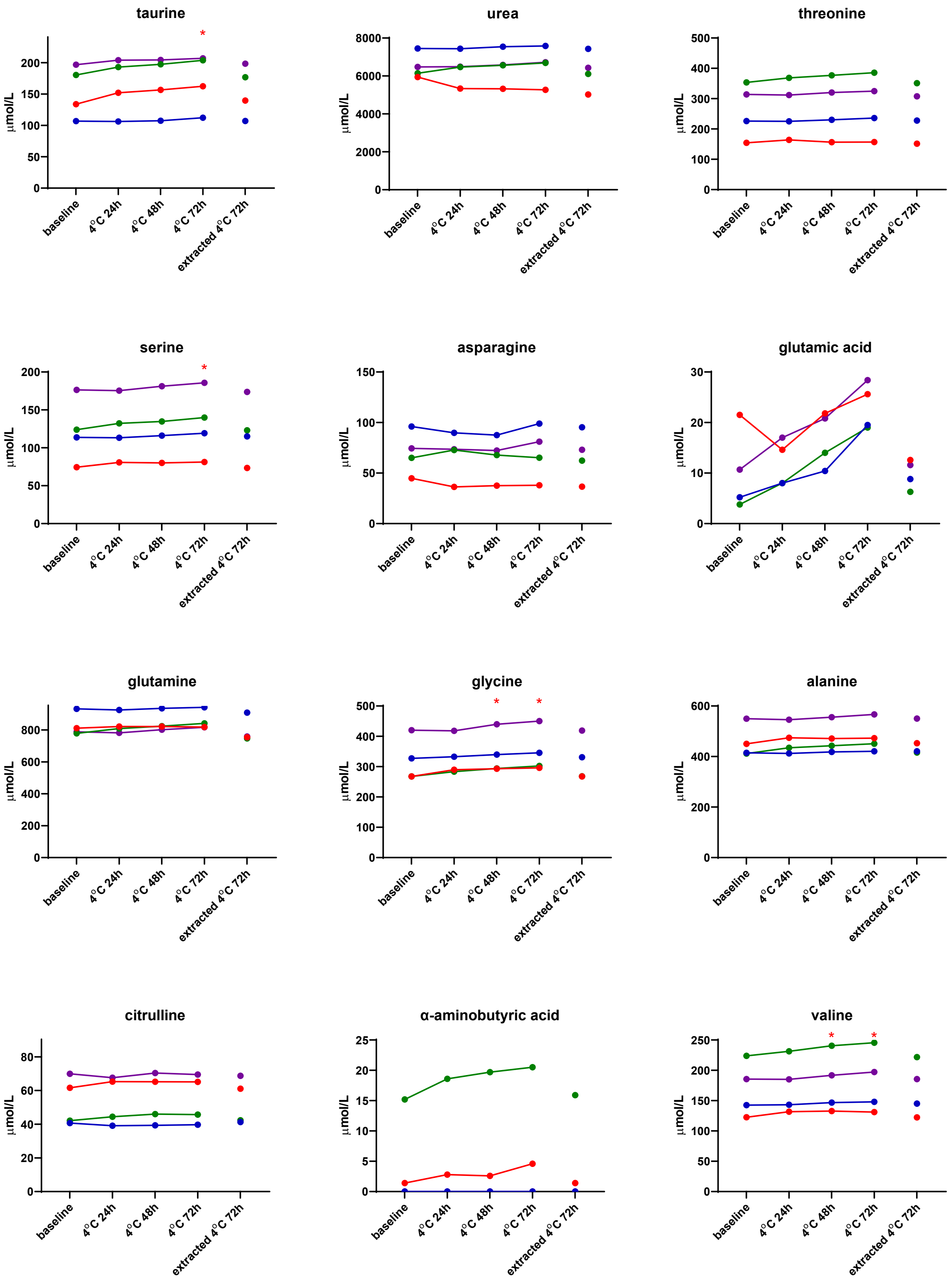

taurine
urea
threonine
serine
asparagine
glutamic acid
glutamine
glycine
alanine
citrulline
α-aminobutyric acid
valine
µmol/L
baseline
4°C 24h
4°C 48h
4°C 72h
extracted 4°C 72h
*

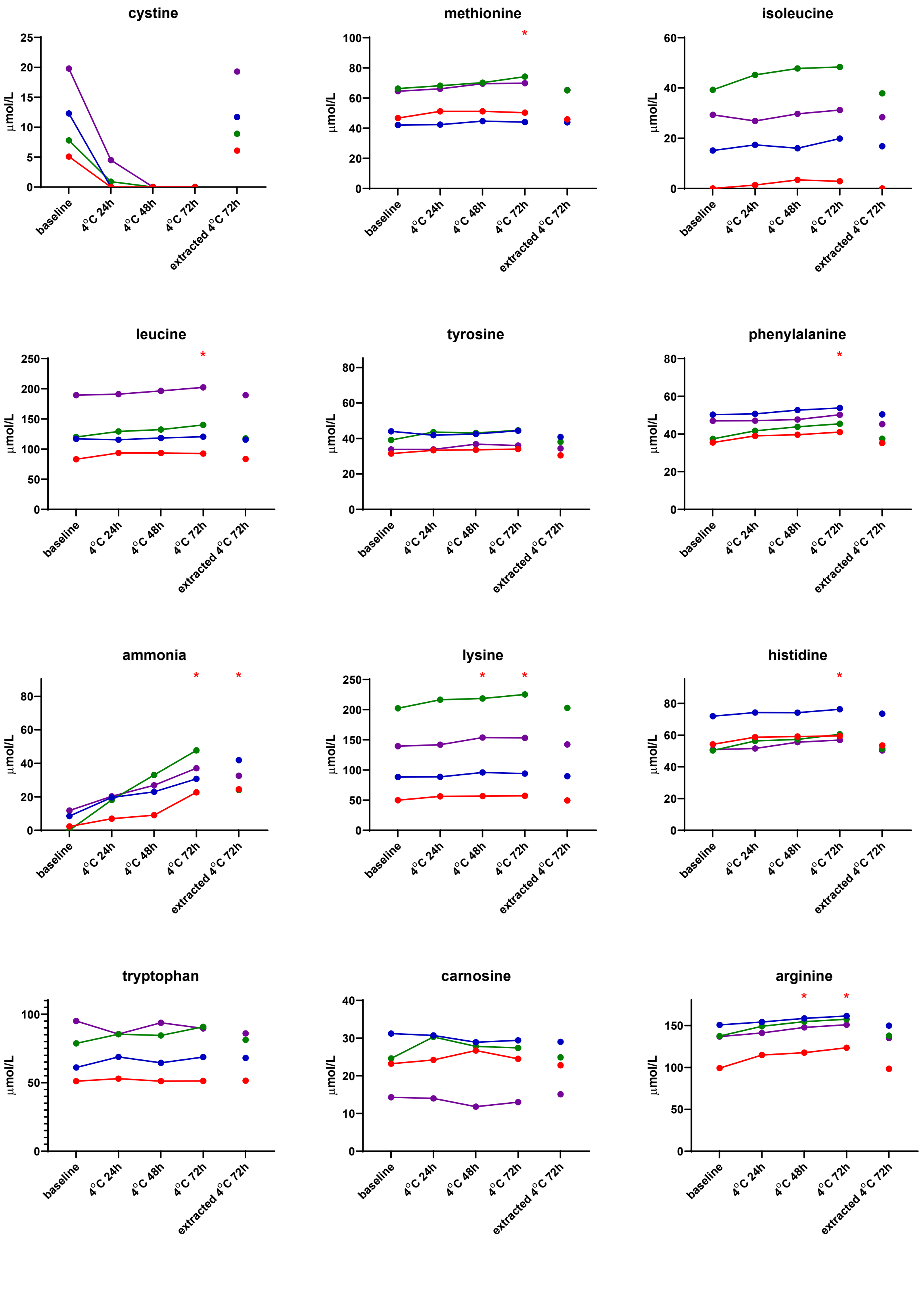
cystine
25
20
15
10
5
0
µmol/L
baseline
4°C 24h
4°C 48h
4°C 72h
extracted 4°C 72h
methionine
100
80
60
40
20
0
µmol/L
*
isoleucine
60
40
20
0
µmol/L
leucine
250
200
150
100
50
0
µmol/L
*
tyrosine
80
60
40
20
0
µmol/L
phenylalanine
80
60
40
20
0
µmol/L
*
ammonia
80
60
40
20
0
µmol/L
*
*
lysine
250
200
150
100
50
0
µmol/L
*
*
histidine
80
60
40
20
0
µmol/L
*
tryptophan
100
50
0
µmol/L
carnosine
40
30
20
10
0
µmol/L
arginine
150
100
50
0
µmol/L
*
*

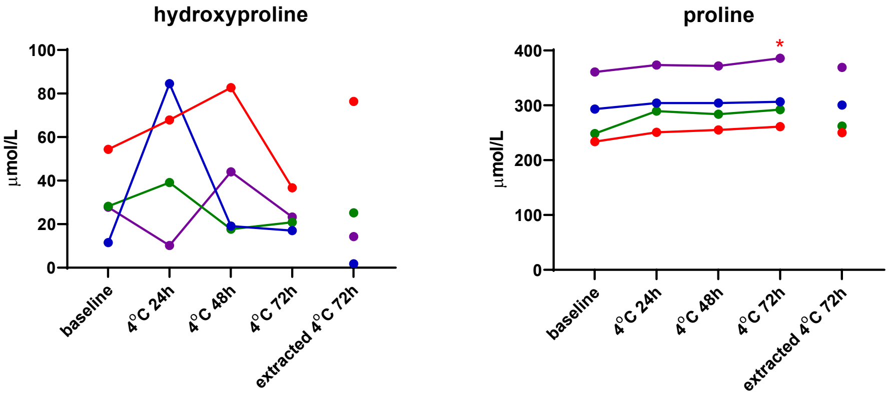

Stability of amino acids in dog serum stored at 4°C. Red asterisks indicate significance ($p<0.05$) compared to baseline.

Stability of amino acids in dog serum stored at 4°C and their coefficients of variation (CV%).

Compound	Median [range] µM[a]	Median [range] CV%[b]
taurine	173 [107-204]	3.7 [1.9-7.1]
urea	6479 [5323-7450]	2.6 [0.8-5.7]
threonine	271 [157-369]	2.3 [1.7-3.6]
serine	124 [80-176]	3.4 [1.9-4.9]
asparagine	69 [38-95]	4.9 [4.2-8.1]
glutamic acid	13 [8-22]	41.7 [25.2-54.1]
glutamine	813 [788-932]	2.8 [1.2-4.2]
glycine	311 [284-420]	3.8 [2.0-4.9]
alanine	453 [418-550]	1.8 [0.8-3.5]
citrulline	55 [40-70]	2.6 [1.4-3.8]
α-aminobutyric acid	1 [0-19]	5.7 [0.0-45.9]
valine	165 [131-231]	3.1 [1.4-3.9]
cystine	0.4 [0-5]	117.8 [103.2-123.2]
methionine	58 [44-68]	4.0 [2.3-4.7]
isoleucine	23 [1-45]	9.7 [4.9-92.9]
leucine	123 [93-191]	4.0 [1.6-6.3]
tyrosine	38 [33-43]	3.8 [3.2-6.3]
phenylalanine	44 [39-51]	4.8 [2.7-7.9]
ammonia	24 [9-27]	54.8 [34.8-67.9]
lysine	116 [56-217]	4.2 [3.4-6.5]
histidine	58 [52-74]	4.8 [1.9-7.0]
tryptophan	76 [51-90]	4.5 [1.3-4.9]
carnosine	26 [14-29]	6.7 [3.1-8.3]
arginine	145 [115-154]	5.0 [2.9-9.1]
hydroxyproline	24 [17-68]	38.8 [25.9-110.1]
proline	294 [251-372]	2.9 [1.5-6.2]

Stability of the assay at 4°C. Compounds excluded from the table because they were not detected in any sample: phosphoserine, phosphoethanolamine, aspartic acid, sarcosine, α-aminoadipic acid, cystathionine, β-alanine, β-aminoisobutyric acid, homocystine, γ-aminobutyric acid, ethanolamine, hydroxylysine, ornithine, 1-methyhistidine, 3-methylhistidine, and anserine. [a]Concentrations of samples used (the median and range of the median of concentrations from four dogs across five different timepoints of storage at 4°C). [b]Coefficient of variation, calculated from the same five timepoints: baseline, storage at 4°C for 24, 48, and 72 hours prior to deproteinization, and storage at 4°C for 72 hours following deproteinization.
