# Supplementary material for: Analytical Validation of an Assay for Concurrent Measurement of Amino Acids in Dog Serum and Comparison of Amino Acid Concentrations between Whole Blood, Plasma, and Serum from Dogs
Source: Metabolites. 2022 Sep 22;12(10):891. doi: 10.3390/metabo12100891 (PMC9608751; doi:10.3390/metabo12100891)
Supplement: Supplementary file 1 [file metabolites-12-00891-s001.zip › File S3.pdf]

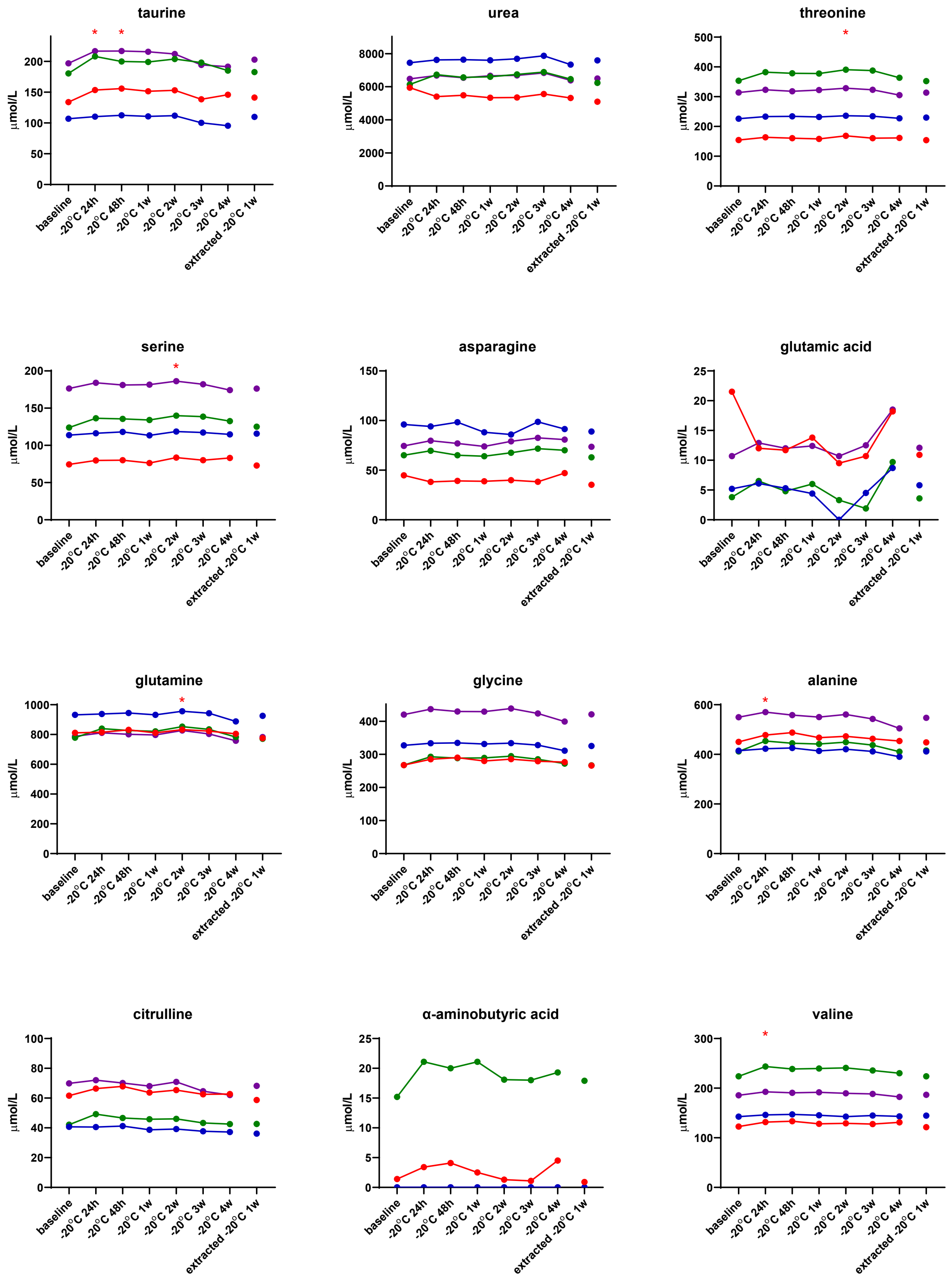
taurine
urea
threonine
serine
asparagine
glutamic acid
glutamine
glycine
alanine
citrulline
α-aminobutyric acid
valine
µmol/L
baseline
-20°C 24h
-20°C 48h
-20°C 1w
-20°C 2w
-20°C 3w
-20°C 4w
extracted -20°C 1w
*

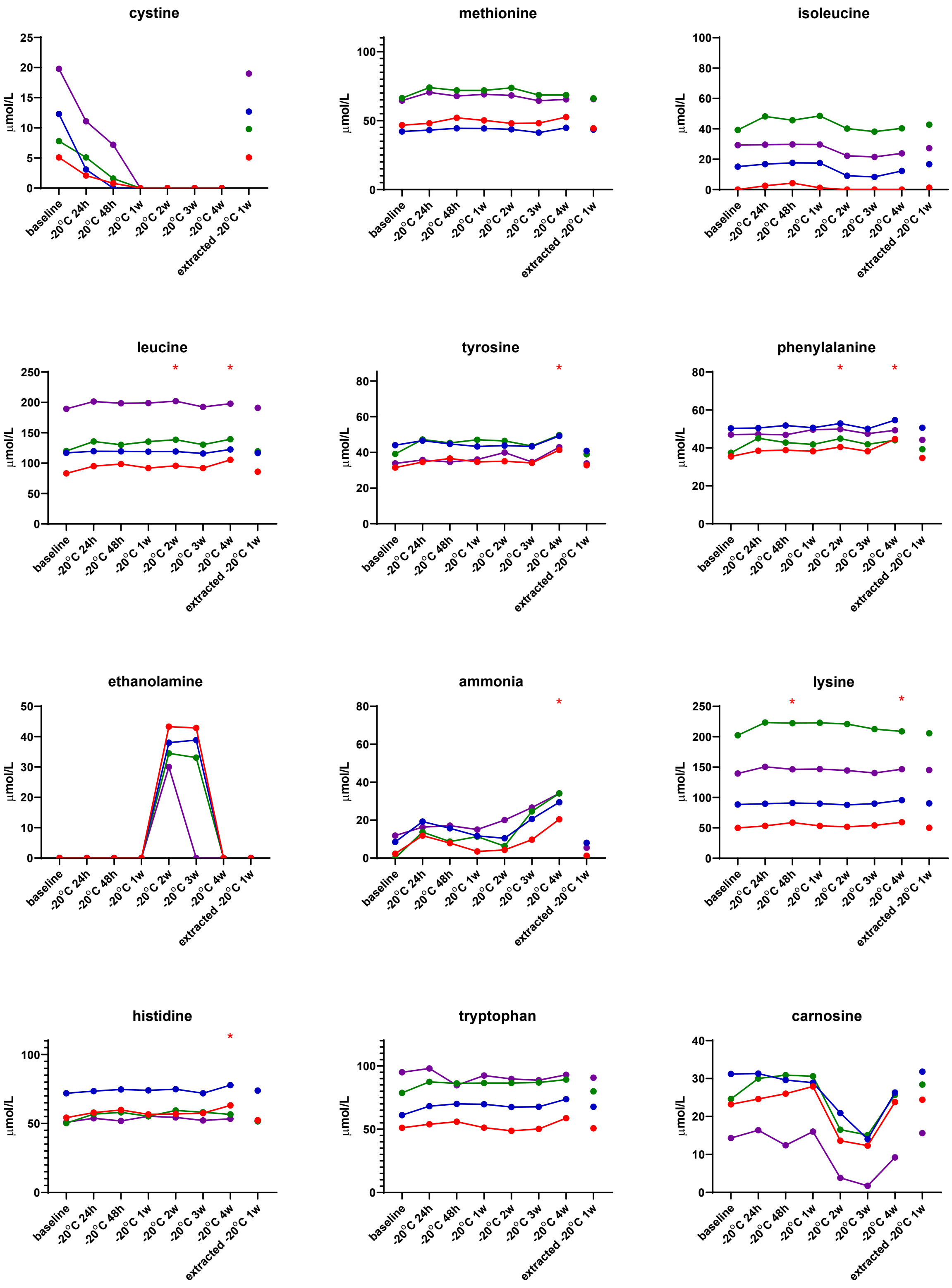

cystine
methionine
isoleucine
leucine
tyrosine
phenylalanine
ethanolamine
ammonia
lysine
histidine
tryptophan
carnosine
μmol/L
baseline
-20°C 24h
-20°C 48h
-20°C 1w
-20°C 2w
-20°C 3w
-20°C 4w
extracted -20°C 1w

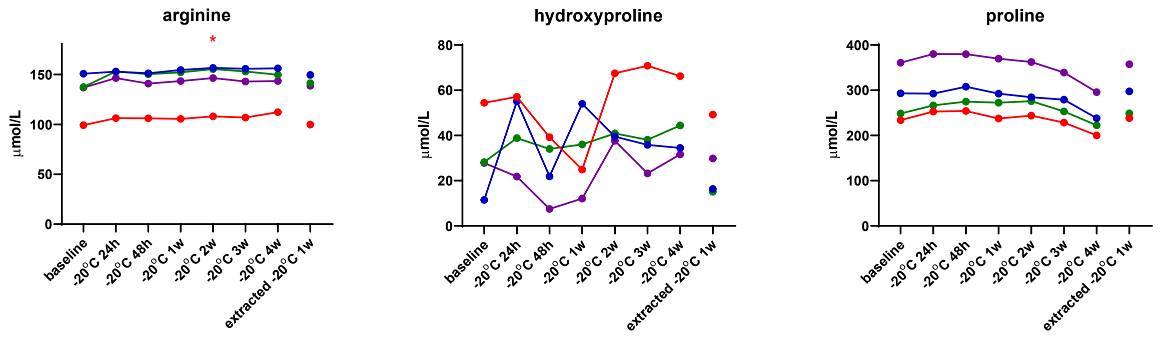

Stability of amino acids in dog serum stored at −20°C. Red asterisks indicate significance ($p<0.05$) compared to baseline.

Stability of amino acids in dog serum stored at −20°C and their coefficients of variation (CV%).

Compound	Median [range] μM[a]	Median [range] CV%[b]
taurine	174 [110-207]	5.1 [4.9-5.4]
urea	6594 [5379-7613]	2.9 [1.9-4.2]
threonine	276 [161-378]	2.5 [1.4-3.8]
serine	125 [80-181]	3.2 [1.5-4.6]
asparagine	72 [39-93]	4.7 [4.1-8.9]
glutamic acid	9[4-12]	37.2 [18.2-45.8]
glutamine	819 [799-935]	2.3 [2.1-3.6]
glycine	308 [280-426]	2.8 [2.2-3.8]
alanine	452 [414-550]	3.1 [2.5-3.8]
citrulline	54 [39-69]	4.5 [4.3-5.3]
α-aminobutyric acid	1 [0-19]	4.9 [0.0-55.9]
valine	167 [129-237]	2.4 [1.1-3.1]
cystine	0.6 [0-4]	126.1 [112.9-150.3]
methionine	57 [44-70]	3.7 [2.5-5.3]
isoleucine	22 [0.6-42]	18.7 [8.9-126.0]
leucine	126 [94-198]	3.9 [1.7-7.0]
tyrosine	40 [35-46]	8.0 [5.3-8.4]
phenylalanine	45 [38-51]	4.8 [2.9-7.4]
ethanolamine	0 [0-0]	173.2 [173.2-264.6]
ammonia	12 [6-17]	61.5 [44.6-89.7]
lysine	118 [53-217]	3.1 [2.3-6.2]
histidine	57 [53-74]	4.0 [2.4-5.4]
tryptophan	77 [51-92]	4.5 [4.1-5.9]
carnosine	26 [13-29]	23.9 [21.9-48.2]
arginine	147 [106-154]	3.0 [1.6-4.0]
hydroxyproline	36 [26-56]	33.3 [24.9-45.1]
proline	276 [238-362]	6.8 [6.6-7.2]

Stability of the assay at −20°C. Compounds excluded from the table because they were not detected in any sample: phosphoserine, phosphoethanolamine, aspartic acid, sarcosine, α-aminoadipic acid, cystathionine, β-alanine, β-aminoisobutyric acid, homocystine, γ-aminobutyric acid, hydroxylysine, ornithine, 1-methyhistidine, 3-methylhistidine, and anserine. [a]Concentrations of samples used (the median and range of the median of concentrations from four dogs across eight different timepoints of storage at −20°C). [b]Coefficient of variation, calculated from the same eight timepoints: baseline, storage at −20°C for 24 and 48 hours, and 1, 2, 3, and 4 weeks prior to deproteinization, and storage at −20°C for 1 week following deproteinization.
