# Supplementary material for: Analytical Validation of an Assay for Concurrent Measurement of Amino Acids in Dog Serum and Comparison of Amino Acid Concentrations between Whole Blood, Plasma, and Serum from Dogs
Source: Metabolites. 2022 Sep 22;12(10):891. doi: 10.3390/metabo12100891 (PMC9608751; doi:10.3390/metabo12100891)
Supplement: Supplementary file 1 [file metabolites-12-00891-s001.zip › File S4.pdf]

Multivariate Column 1=1-methylhistidine

Correlations

	whole blood	plasma	serum
whole blood	1.0000	0.9666	0.9812
plasma	0.9666	1.0000	0.9826
serum	0.9812	0.9826	1.0000

The correlations are estimated by Row-wise method.

Scatterplot Matrix

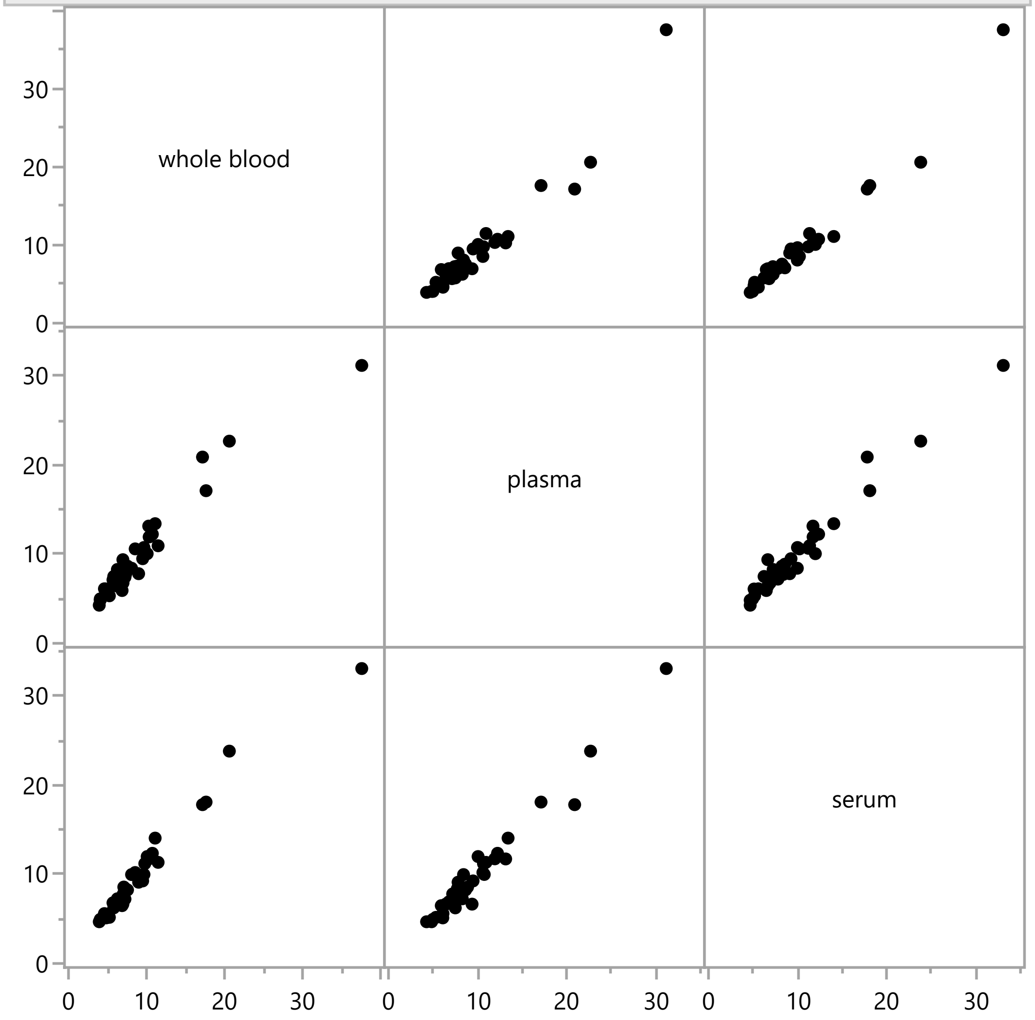

Nonparametric: Spearman's ρ

Variable	by Variable	Spearman ρ	Prob>|ρ|	-.8 -.6 -.4 -.2 0 .2 .4 .6 .8
plasma	whole blood	0.9418	<.0001*	
serum	whole blood	0.9763	<.0001*	
serum	plasma	0.9500	<.0001*	

Multivariate Column 1=3-methylhistidine

Correlations

	whole blood	plasma	serum
whole blood	1.0000	0.9786	0.9818
plasma	0.9786	1.0000	0.9876
serum	0.9818	0.9876	1.0000

The correlations are estimated by Row-wise method.

Scatterplot Matrix

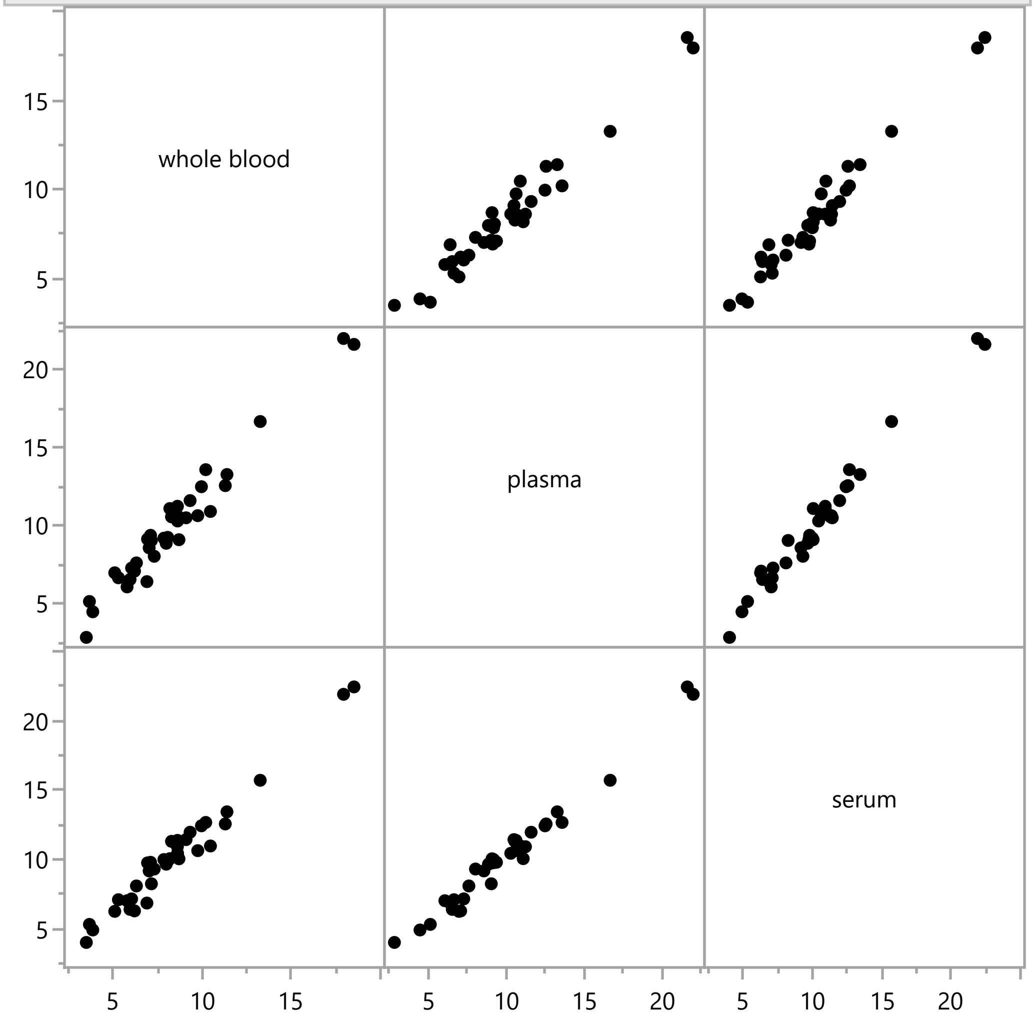

Nonparametric: Spearman's ρ

Variable	by Variable	Spearman ρ	Prob>|ρ|	-.8 -.6 -.4 -.2 0 .2 .4 .6 .8
plasma	whole blood	0.9472	<.0001*	
serum	whole blood	0.9663	<.0001*	
serum	plasma	0.9725	<.0001*	

Multivariate Column 1=alanine

Correlations

	whole blood	plasma	serum
whole blood	1.0000	0.9148	0.9312
plasma	0.9148	1.0000	0.9896
serum	0.9312	0.9896	1.0000

The correlations are estimated by Row-wise method.

Scatterplot Matrix

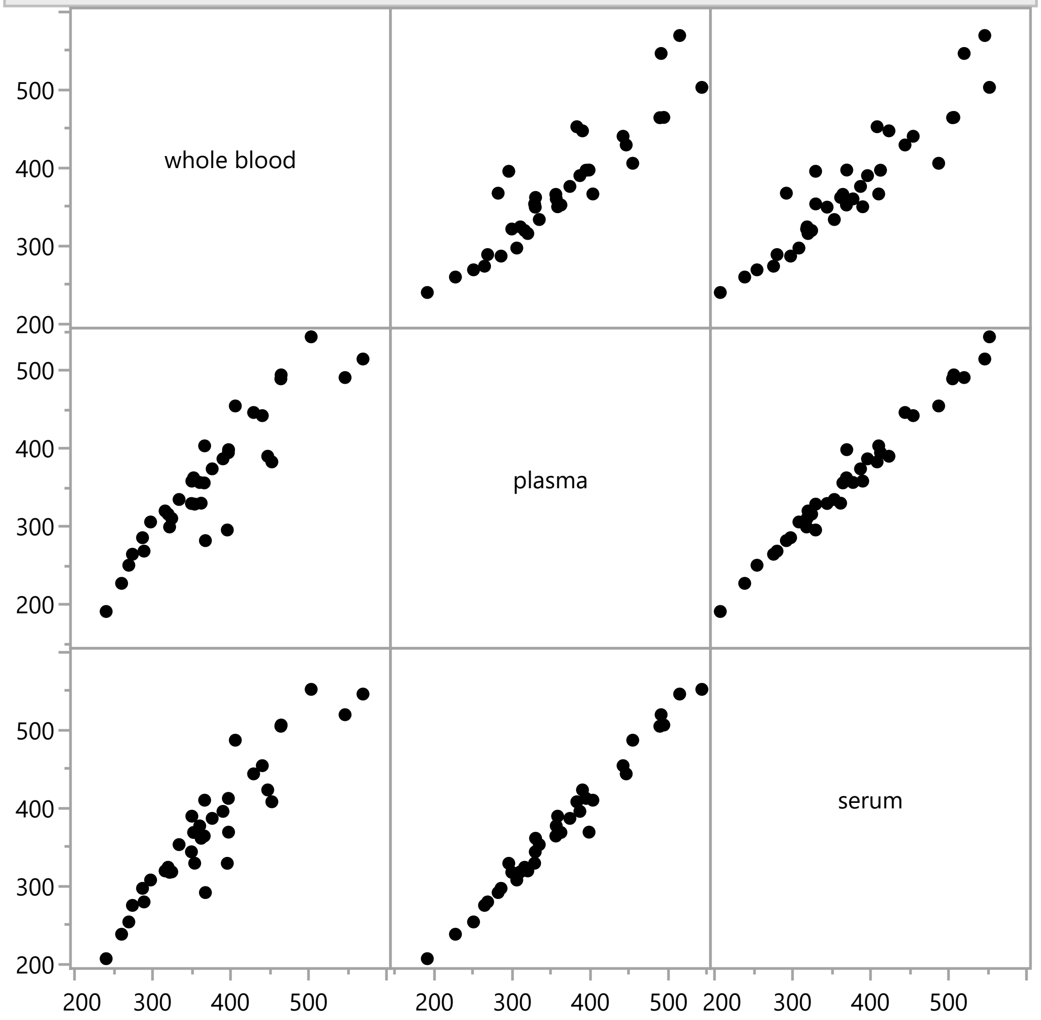

Nonparametric: Spearman's ρ

Variable	by Variable	Spearman ρ	Prob>|ρ|	-.8 -.6 -.4 -.2 0 .2 .4 .6 .8
plasma	whole blood	0.8932	<.0001*	
serum	whole blood	0.9161	<.0001*	
serum	plasma	0.9840	<.0001*	

Multivariate Column 1=alpha-aminoadipic acid

Correlations

	whole blood	plasma	serum
whole blood	1.0000	0.2917	0.2778
plasma	0.2917	1.0000	0.9103
serum	0.2778	0.9103	1.0000

The correlations are estimated by Row-wise method.

Scatterplot Matrix

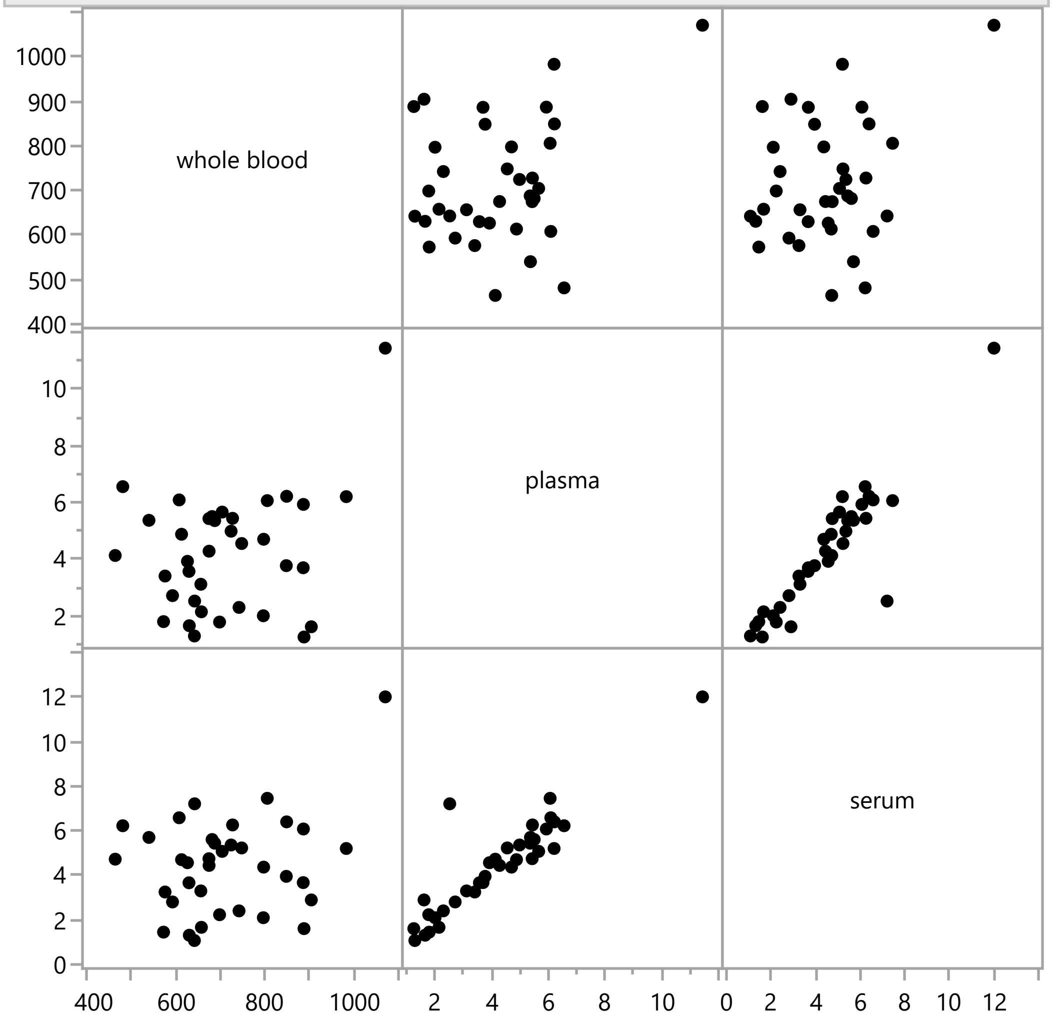

Nonparametric: Spearman's ρ

Variable	by Variable	Spearman ρ	Prob>|ρ|	-.8 -.6 -.4 -.2 0 .2 .4 .6 .8
plasma	whole blood	0.1387	0.4197	
serum	whole blood	0.1333	0.4382	
serum	plasma	0.8728	<.0001*	

Multivariate Column 1=alpha-aminobutyric acid

Correlations

	whole blood	plasma	serum
whole blood	1.0000	0.9894	0.9915
plasma	0.9894	1.0000	0.9977
serum	0.9915	0.9977	1.0000

The correlations are estimated by Row-wise method.

Scatterplot Matrix

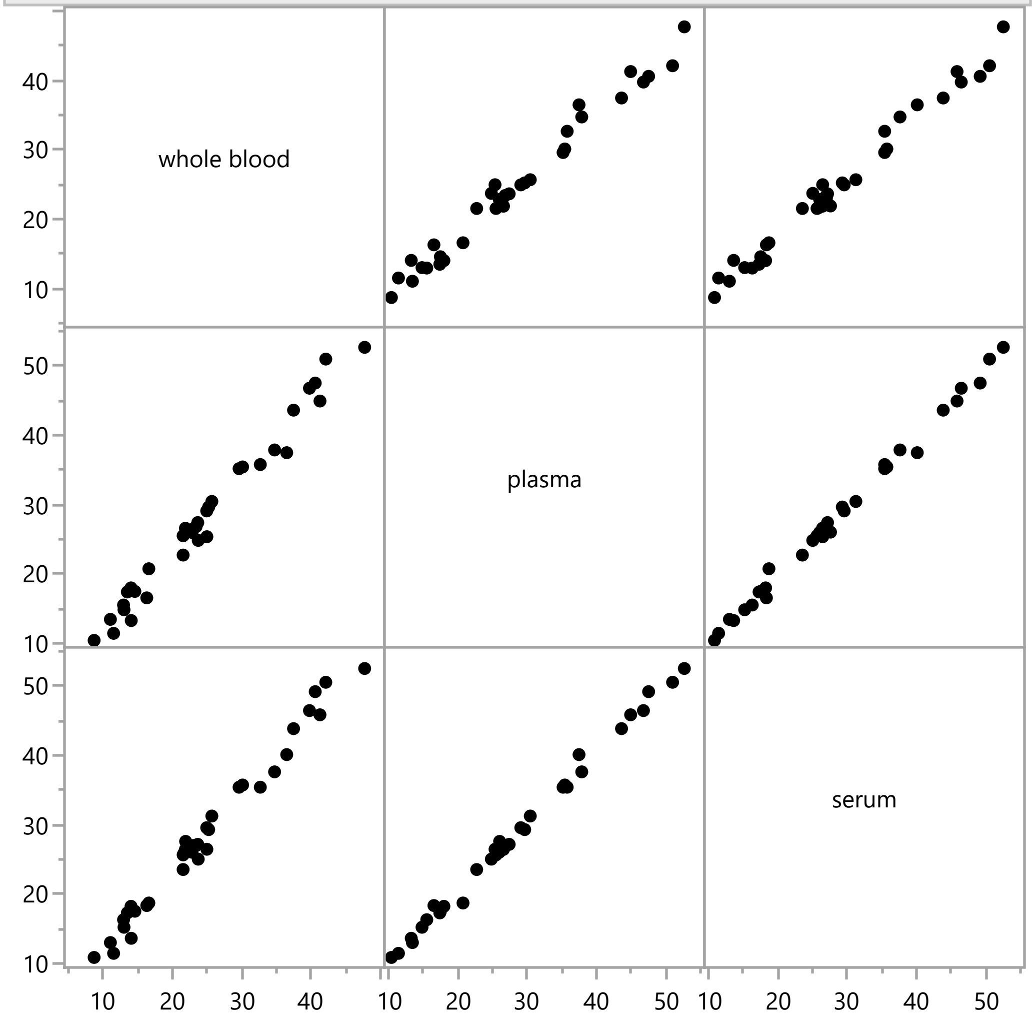

Nonparametric: Spearman's ρ

Variable	by Variable	Spearman ρ	Prob>|ρ|	-.8 -.6 -.4 -.2 0 .2 .4 .6 .8
plasma	whole blood	0.9671	<.0001*	
serum	whole blood	0.9773	<.0001*	
serum	plasma	0.9913	<.0001*	

Multivariate Column 1=ammonia

Correlations

	whole blood	plasma	serum
whole blood	1.0000	0.4371	0.5308
plasma	0.4371	1.0000	0.6422
serum	0.5308	0.6422	1.0000

The correlations are estimated by Row-wise method.

Scatterplot Matrix

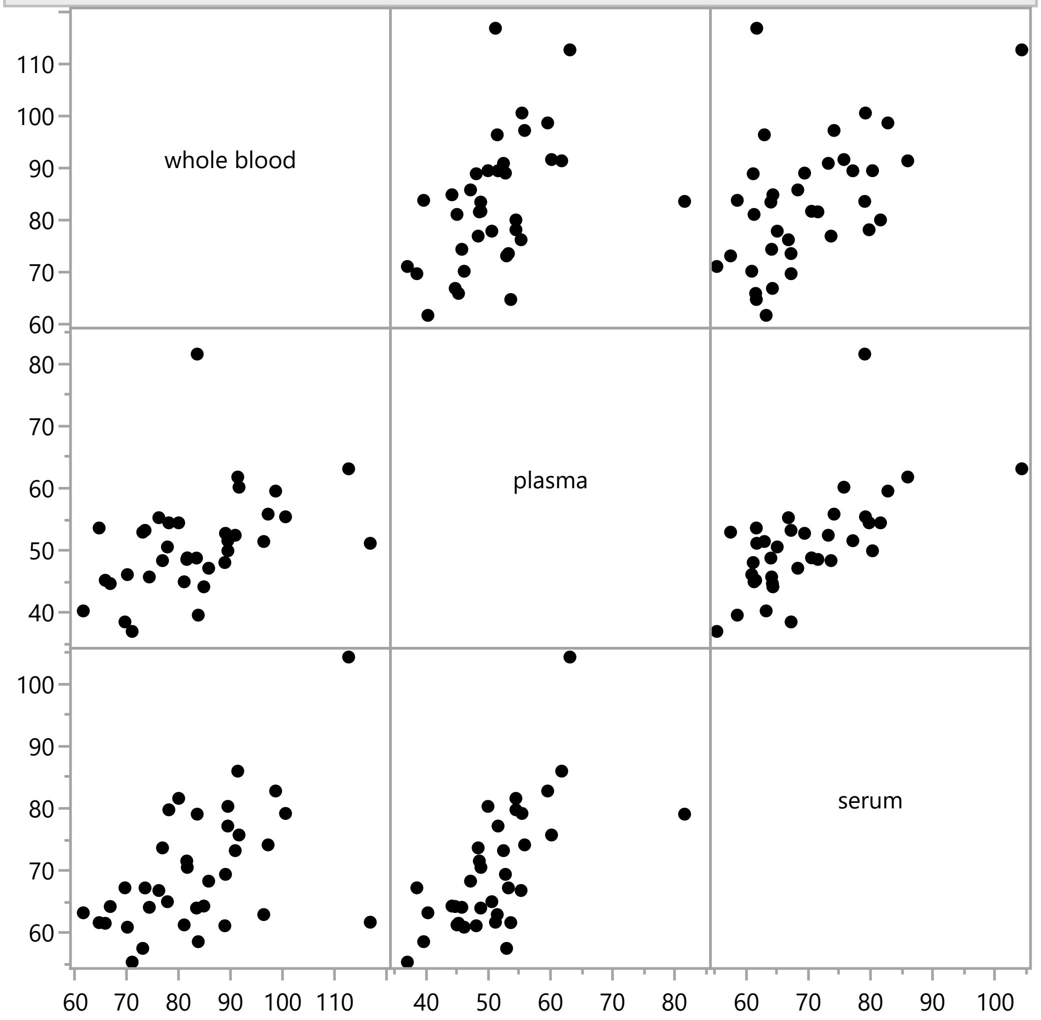

Nonparametric: Spearman's ρ

Variable	by Variable	Spearman ρ	Prob>|ρ|	-.8 -.6 -.4 -.2 0 .2 .4 .6 .8
plasma	whole blood	0.5145	0.0013*	
serum	whole blood	0.5115	0.0014*	
serum	plasma	0.6649	<.0001*	

Multivariate Column 1=anserine

Correlations

	whole blood	plasma	serum
whole blood	1.0000	0.7705	0.8136
plasma	0.7705	1.0000	0.9341
serum	0.8136	0.9341	1.0000

The correlations are estimated by Row-wise method.

Scatterplot Matrix

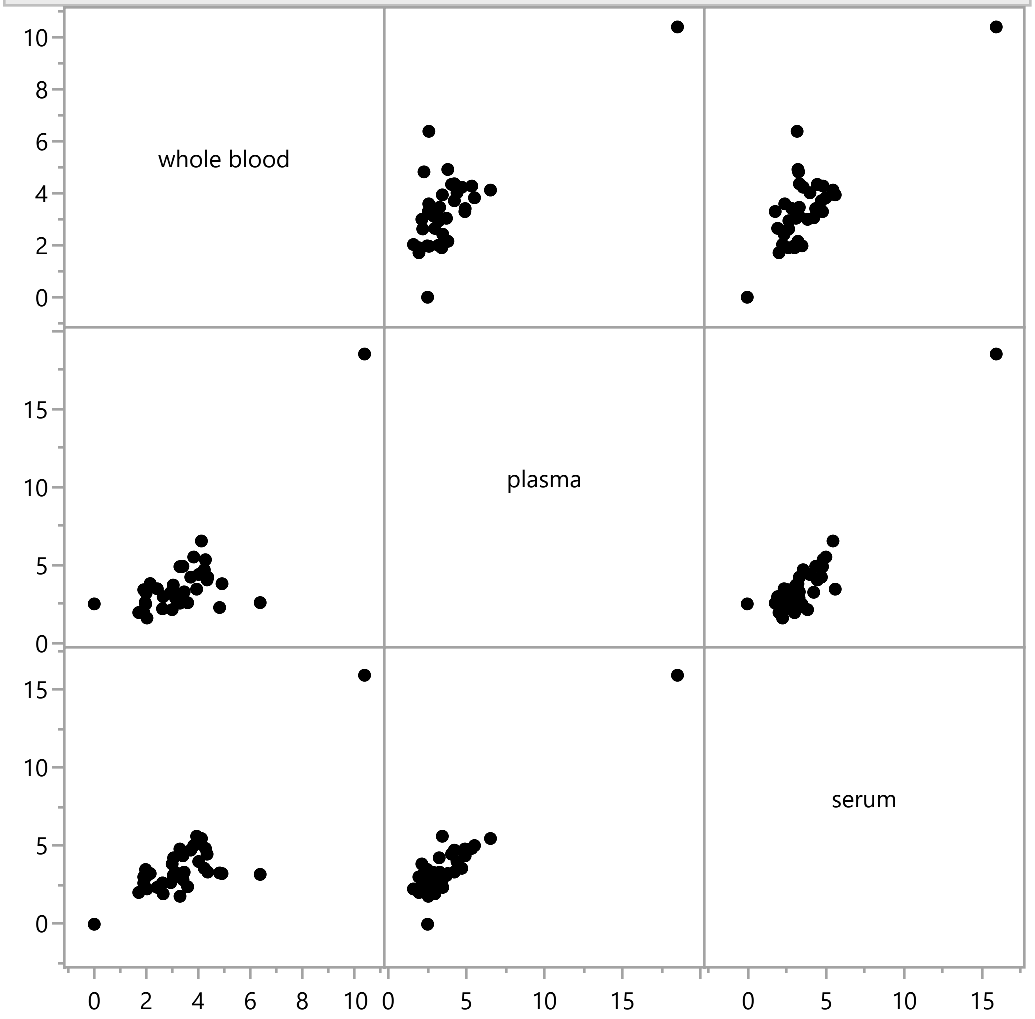

Nonparametric: Spearman's ρ

Variable	by Variable	Spearman ρ	Prob>|ρ|	-.8 -.6 -.4 -.2 0 .2 .4 .6 .8
plasma	whole blood	0.5576	0.0004*	
serum	whole blood	0.6108	<.0001*	
serum	plasma	0.6982	<.0001*	

Multivariate Column 1=arginine

Correlations

	whole blood	plasma	serum
whole blood	1.0000	0.8308	0.7828
plasma	0.8308	1.0000	0.9178
serum	0.7828	0.9178	1.0000

The correlations are estimated by Row-wise method.

Scatterplot Matrix

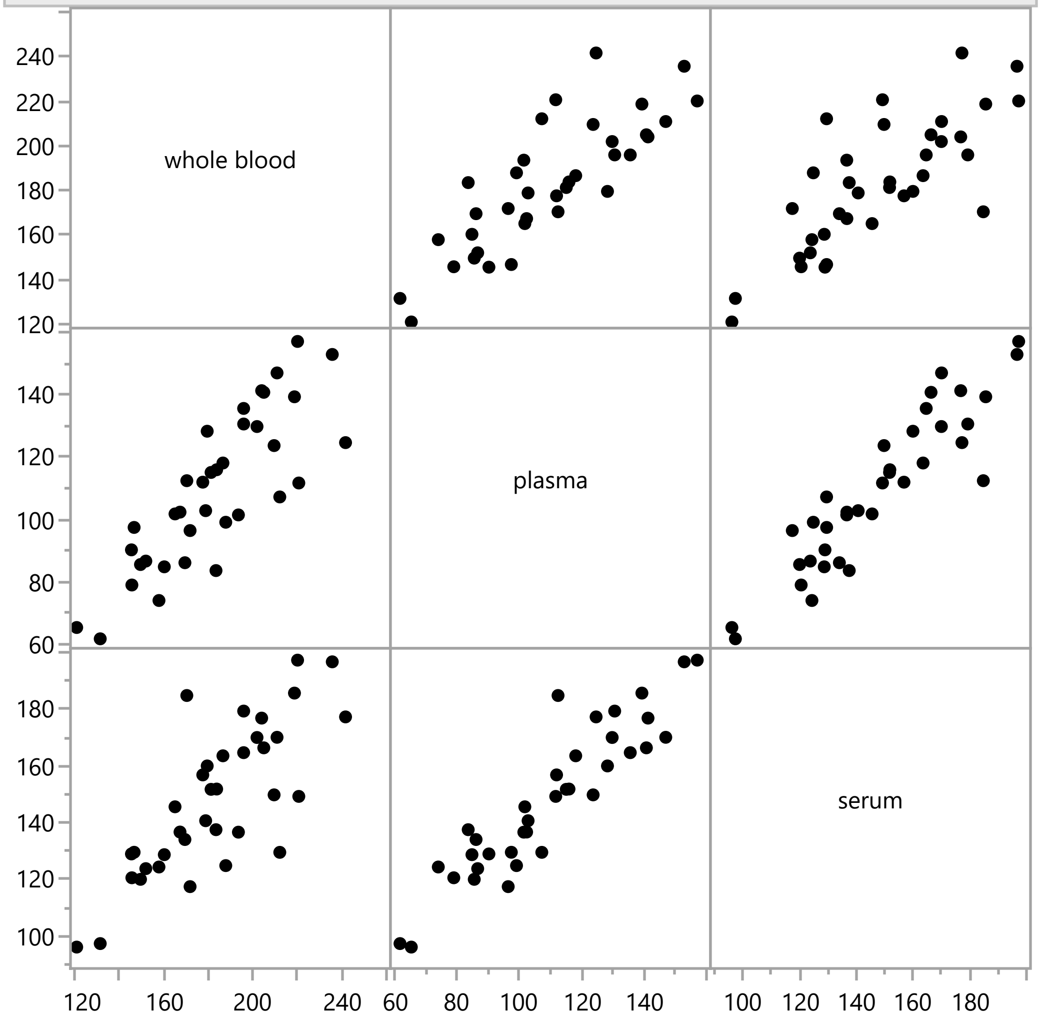

Nonparametric: Spearman's ρ

Variable	by Variable	Spearman ρ	Prob>|ρ|	-.8 -.6 -.4 -.2 0 .2 .4 .6 .8
plasma	whole blood	0.8147	<.0001*	
serum	whole blood	0.7493	<.0001*	
serum	plasma	0.9166	<.0001*	

Multivariate Column 1=asparagine

Correlations

	whole blood	plasma	serum
whole blood	1.0000	0.7926	0.8522
plasma	0.7926	1.0000	0.9700
serum	0.8522	0.9700	1.0000

The correlations are estimated by Row-wise method.

Scatterplot Matrix

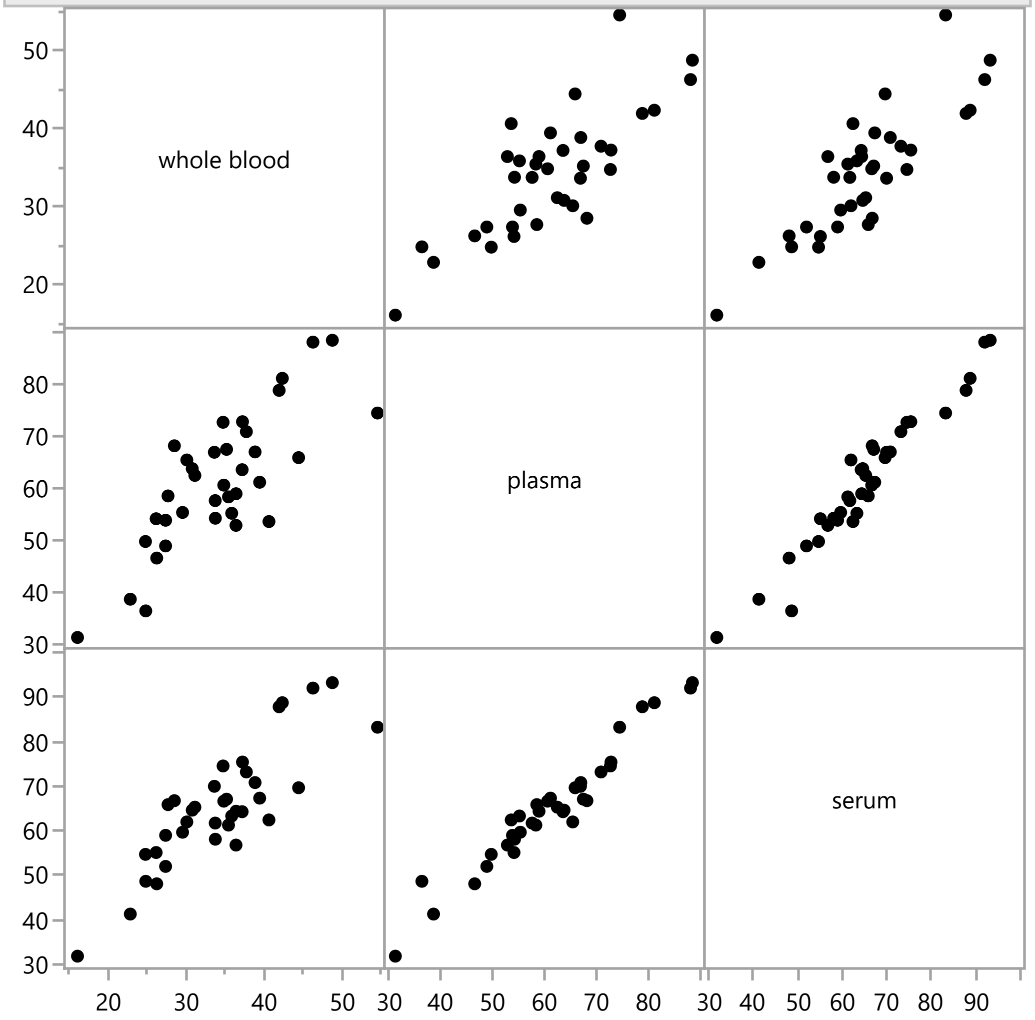

Nonparametric: Spearman's ρ

Variable	by Variable	Spearman ρ	Prob>|ρ|	-.8 -.6 -.4 -.2 0 .2 .4 .6 .8
plasma	whole blood	0.7197	<.0001*	
serum	whole blood	0.7951	<.0001*	
serum	plasma	0.9575	<.0001*	

Multivariate Column 1=aspartic acid

Correlations

	whole blood	plasma	serum
whole blood	1.0000	0.1509	0.0398
plasma	0.1509	1.0000	0.7560
serum	0.0398	0.7560	1.0000

The correlations are estimated by Row-wise method.

Scatterplot Matrix

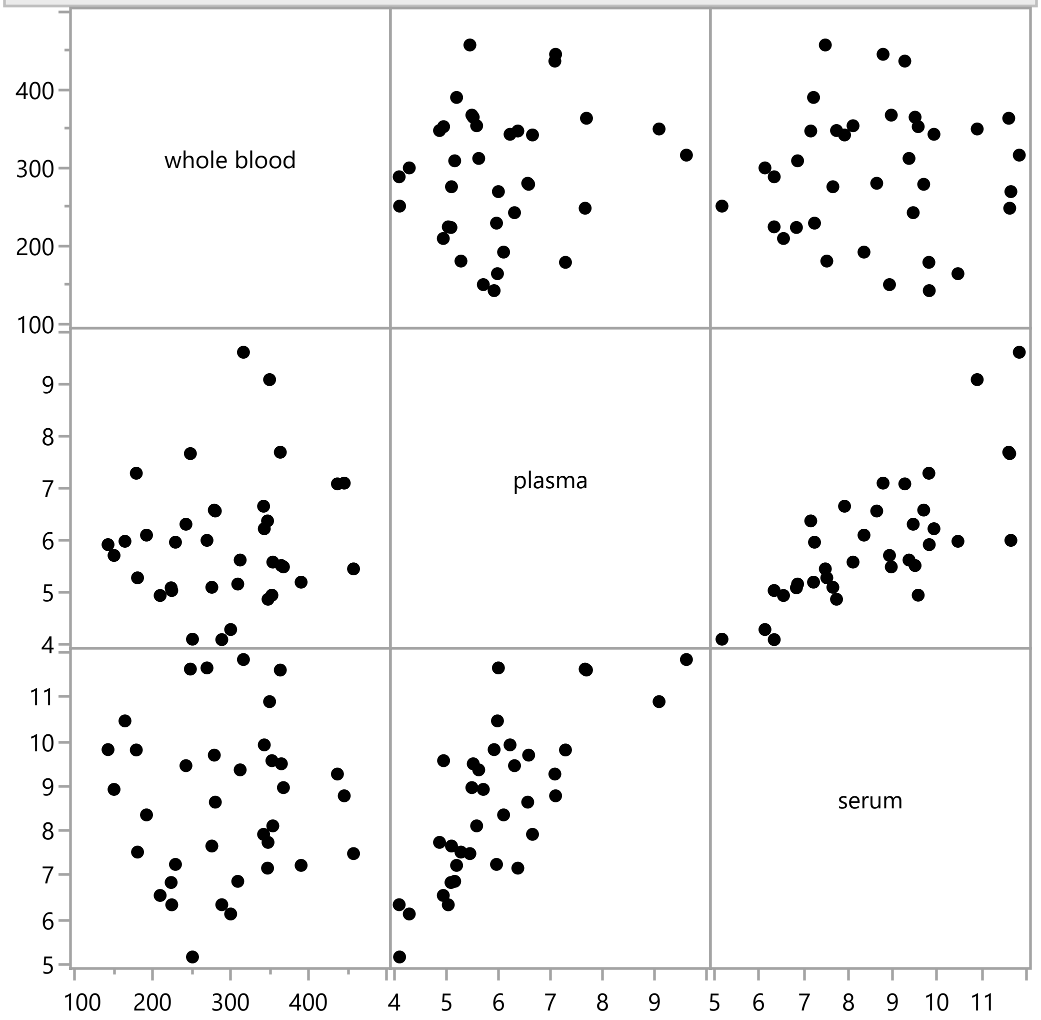

Nonparametric: Spearman's ρ

Variable	by Variable	Spearman ρ	Prob>|ρ|	-.8 -.6 -.4 -.2 0 .2 .4 .6 .8
plasma	whole blood	0.1027	0.5511	
serum	whole blood	0.0479	0.7816	
serum	plasma	0.7256	<.0001*	

Multivariate Column 1=beta-alanine

Correlations

	whole blood	plasma	serum
whole blood	1.0000	0.3815	0.3690
plasma	0.3815	1.0000	0.5344
serum	0.3690	0.5344	1.0000

The correlations are estimated by Row-wise method.

Scatterplot Matrix

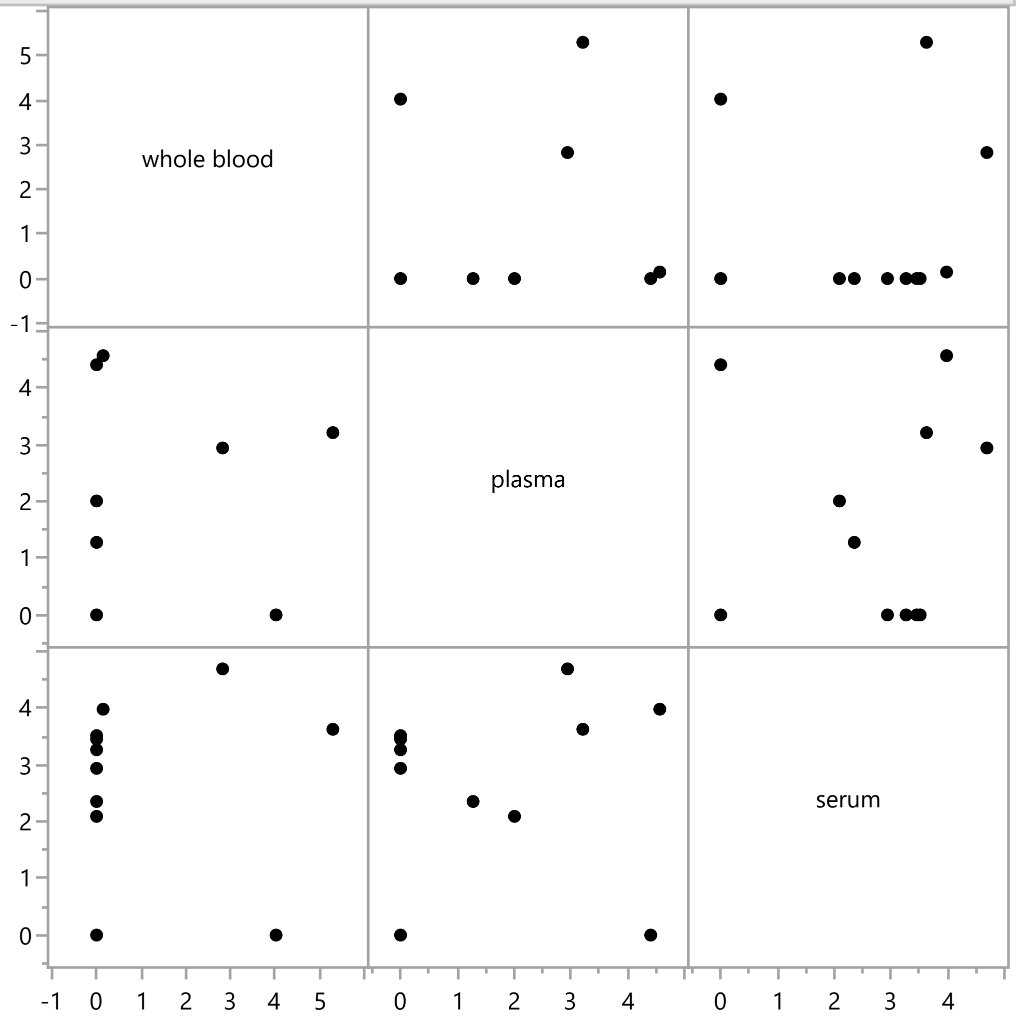

Nonparametric: Spearman's ρ

Variable	by Variable	Spearman ρ	Prob>|ρ|	-.8 -.6 -.4 -.2 0 .2 .4 .6 .8
plasma	whole blood	0.5734	0.0003*	
serum	whole blood	0.4946	0.0022*	
serum	plasma	0.6063	<.0001*	

Multivariate Column 1=carnosine

Correlations

	whole blood	plasma	serum
whole blood	1.0000	0.7896	0.8249
plasma	0.7896	1.0000	0.9558
serum	0.8249	0.9558	1.0000

The correlations are estimated by Row-wise method.

Scatterplot Matrix

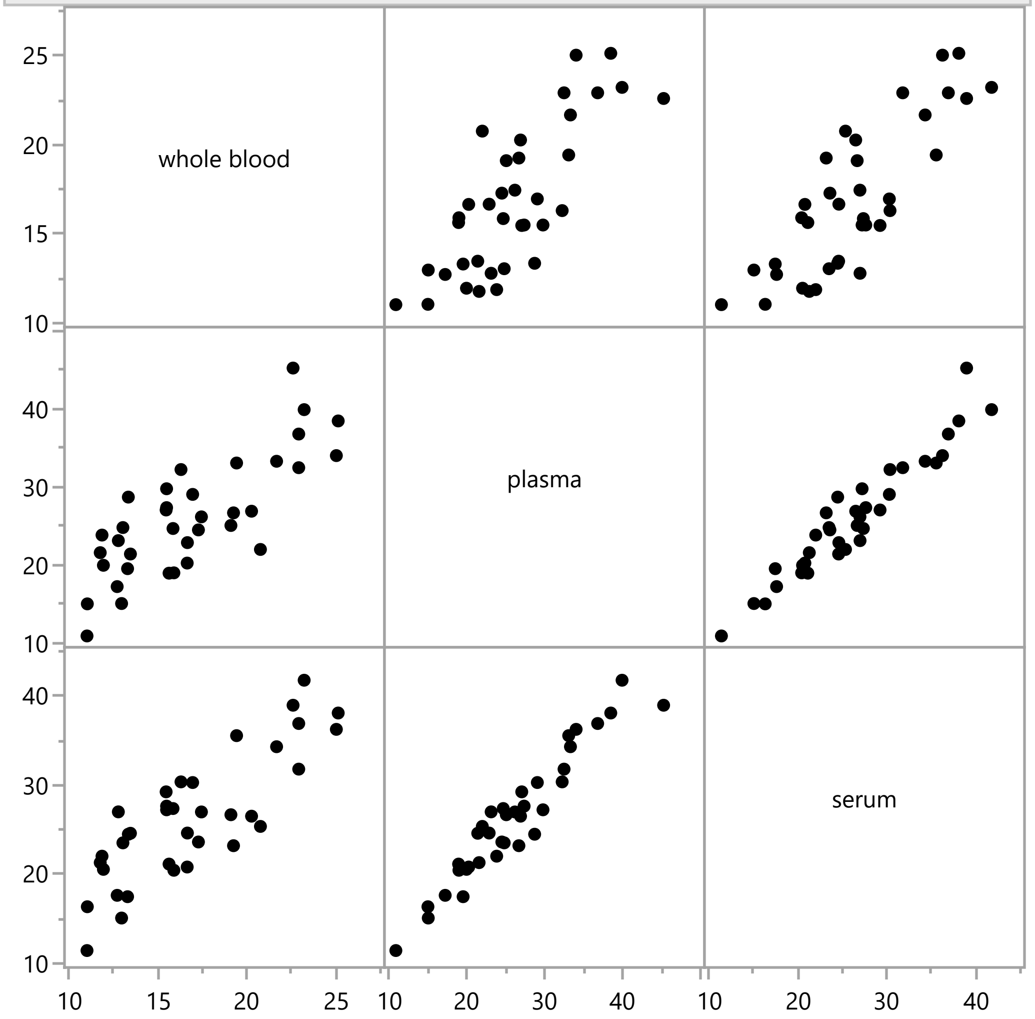

Nonparametric: Spearman's ρ

Variable	by Variable	Spearman ρ	Prob>|ρ|	-.8 -.6 -.4 -.2 0 .2 .4 .6 .8
plasma	whole blood	0.7467	<.0001*	
serum	whole blood	0.7576	<.0001*	
serum	plasma	0.9356	<.0001*	

Multivariate Column 1=citrulline

Correlations

	whole blood	plasma	serum
whole blood	1.0000	0.9299	0.9474
plasma	0.9299	1.0000	0.9929
serum	0.9474	0.9929	1.0000

The correlations are estimated by Row-wise method.

Scatterplot Matrix

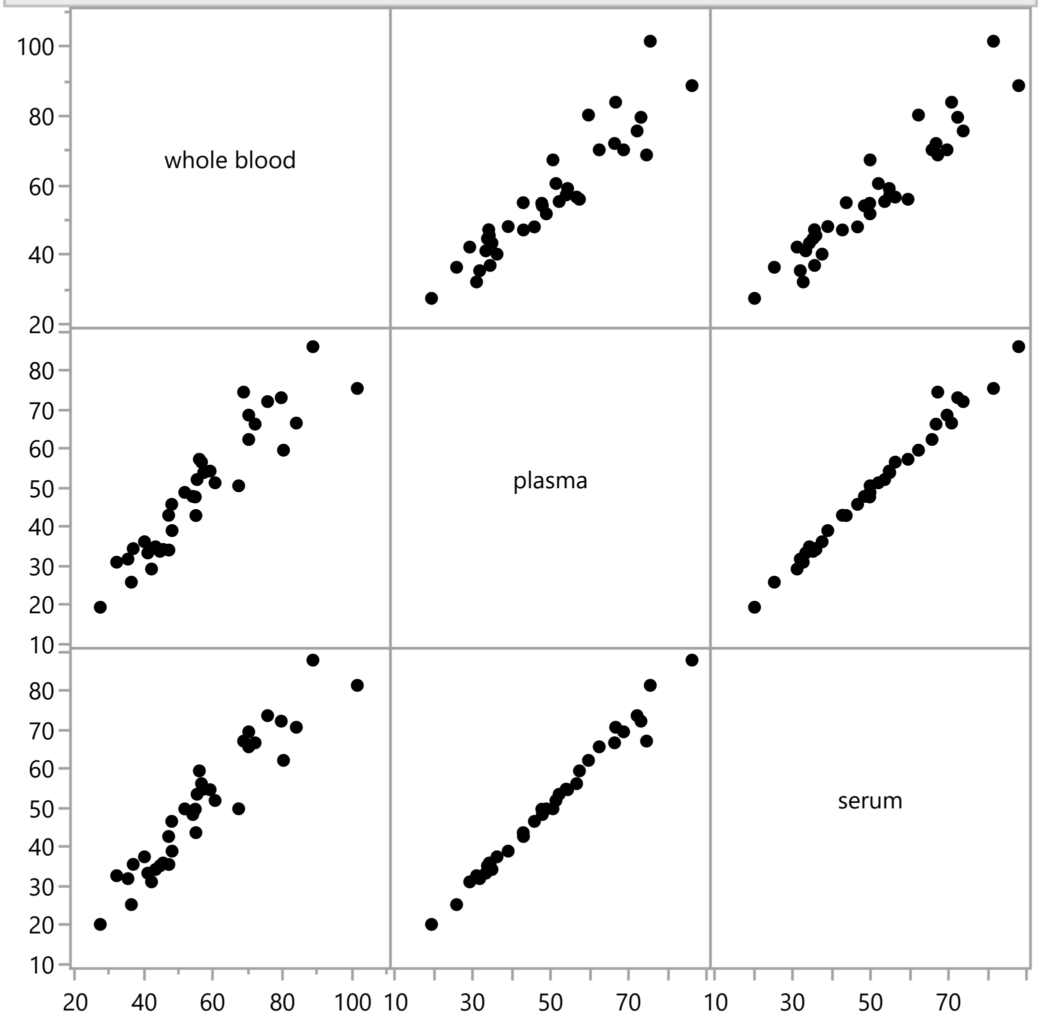

Nonparametric: Spearman's ρ

Variable	by Variable	Spearman ρ	Prob>|ρ|	-.8 -.6 -.4 -.2 0 .2 .4 .6 .8
plasma	whole blood	0.9493	<.0001*	
serum	whole blood	0.9588	<.0001*	
serum	plasma	0.9931	<.0001*	

Multivariate Column 1=cystathionine

Correlations

	whole blood	plasma	serum
whole blood	1.0000	0.7643	0.7263
plasma	0.7643	1.0000	0.9814
serum	0.7263	0.9814	1.0000

The correlations are estimated by Row-wise method.

Scatterplot Matrix

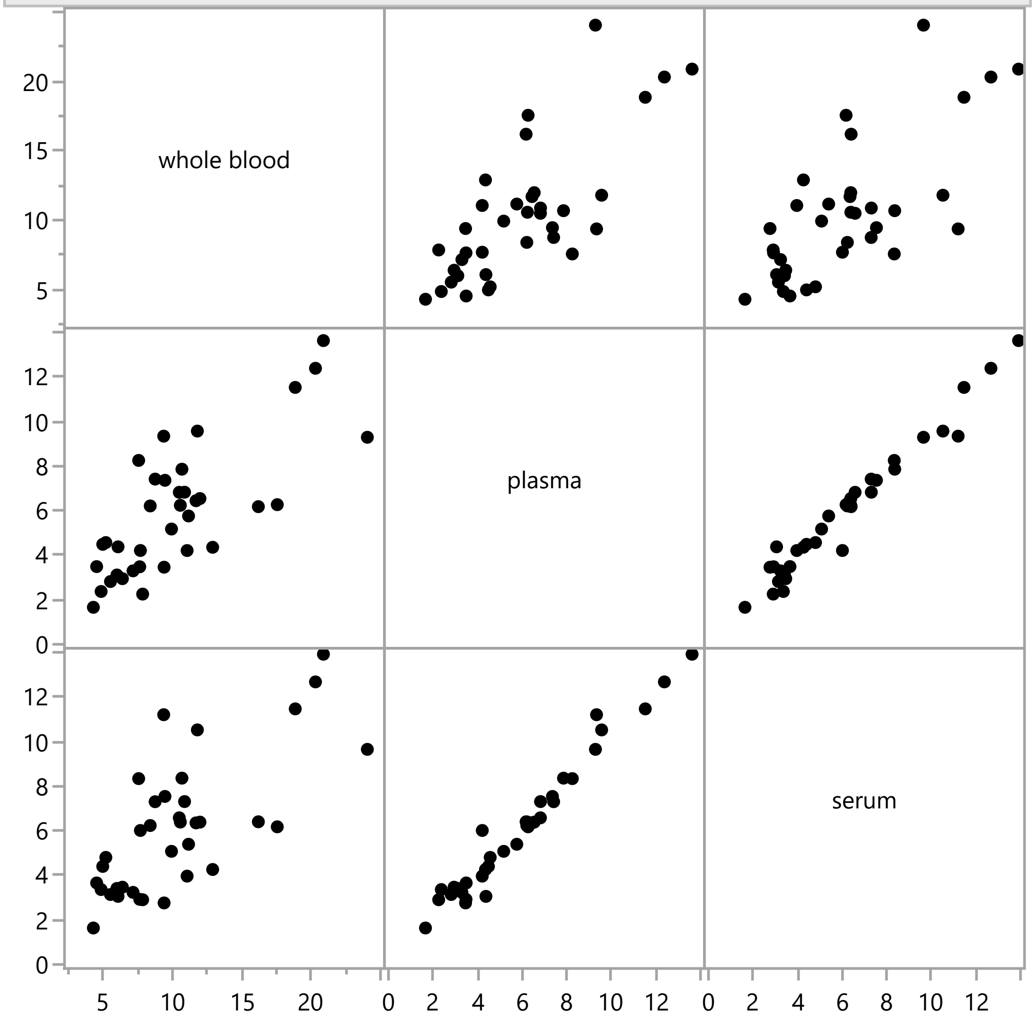

Nonparametric: Spearman's ρ

Variable	by Variable	Spearman ρ	Prob>|ρ|	-.8 -.6 -.4 -.2 0 .2 .4 .6 .8
plasma	whole blood	0.6914	<.0001*	
serum	whole blood	0.6708	<.0001*	
serum	plasma	0.9609	<.0001*	

Multivariate Column 1=cystine

Correlations

	whole blood	plasma	serum
whole blood	1.0000	0.2886	0.1384
plasma	0.2886	1.0000	0.8466
serum	0.1384	0.8466	1.0000

The correlations are estimated by Row-wise method.

Scatterplot Matrix

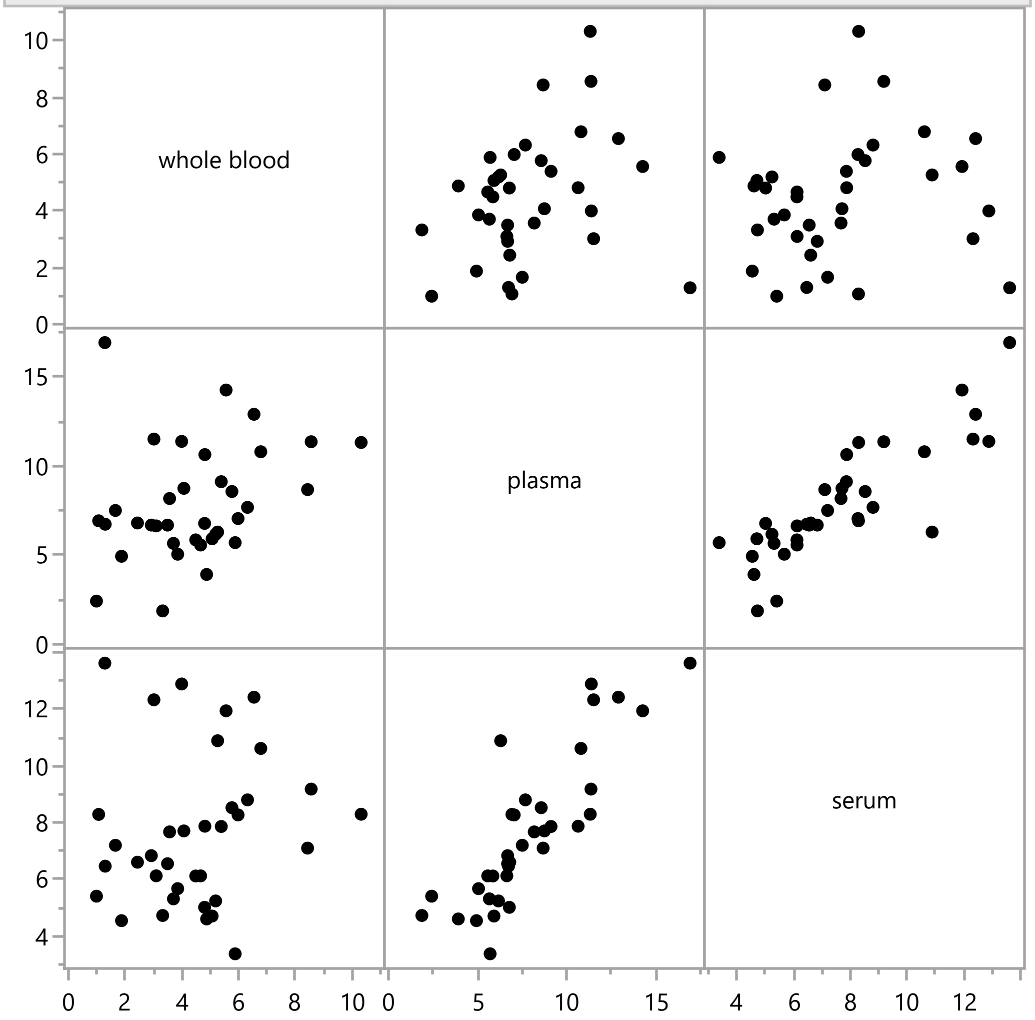

Nonparametric: Spearman's ρ

Variable	by Variable	Spearman ρ	Prob>|ρ|	-.8 -.6 -.4 -.2 0 .2 .4 .6 .8
plasma	whole blood	0.3230	0.0546	
serum	whole blood	0.2414	0.1560	
serum	plasma	0.8708	<.0001*	

Multivariate Column 1=ethanolamine

Correlations

	whole blood	plasma	serum
whole blood	1.0000	0.3227	0.3652
plasma	0.3227	1.0000	0.8376
serum	0.3652	0.8376	1.0000

The correlations are estimated by Row-wise method.

Scatterplot Matrix

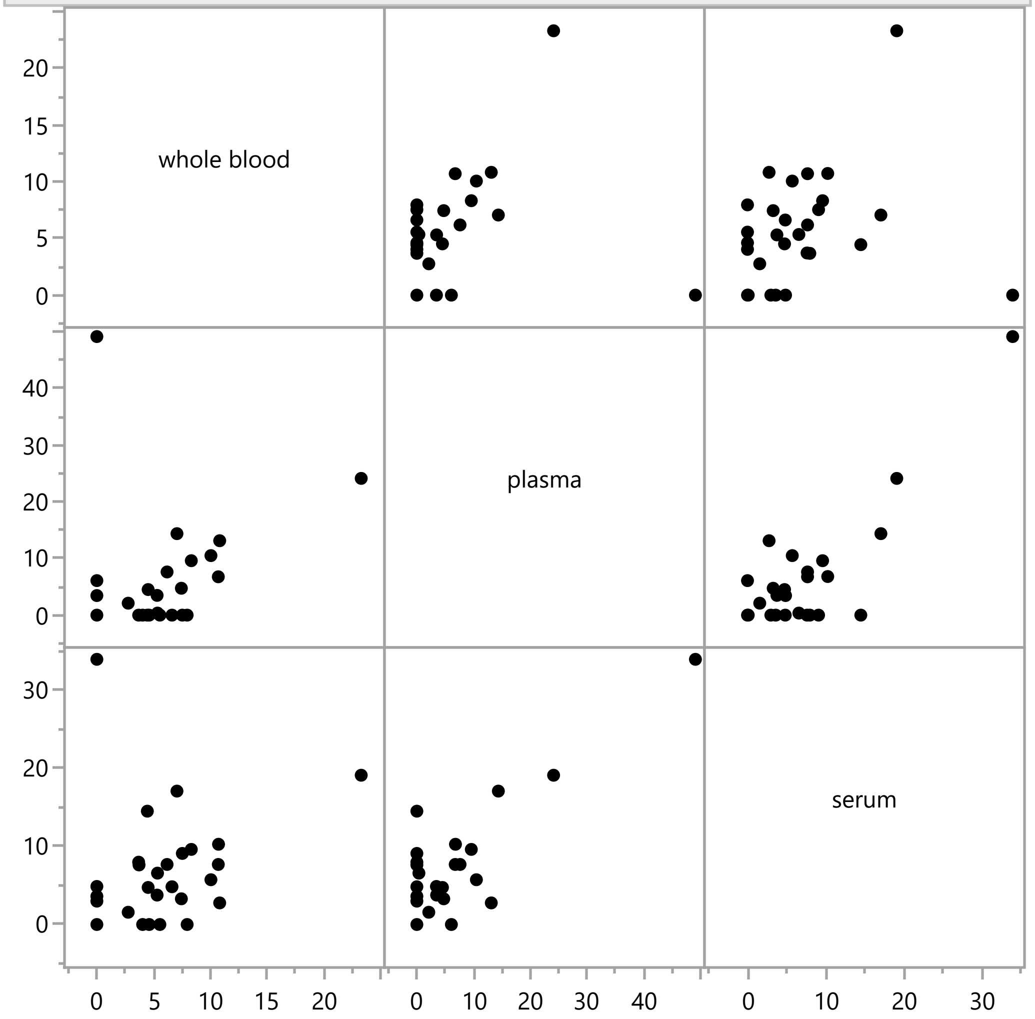

Nonparametric: Spearman's ρ

Variable	by Variable	Spearman ρ	Prob>|ρ|	-.8 -.6 -.4 -.2 0 .2 .4 .6 .8
plasma	whole blood	0.5354	0.0008*	
serum	whole blood	0.4981	0.0020*	
serum	plasma	0.5810	0.0002*	

Multivariate Column 1=glutamic acid

Correlations

	whole blood	plasma	serum
whole blood	1.0000	0.4822	0.4733
plasma	0.4822	1.0000	0.7615
serum	0.4733	0.7615	1.0000

The correlations are estimated by Row-wise method.

Scatterplot Matrix

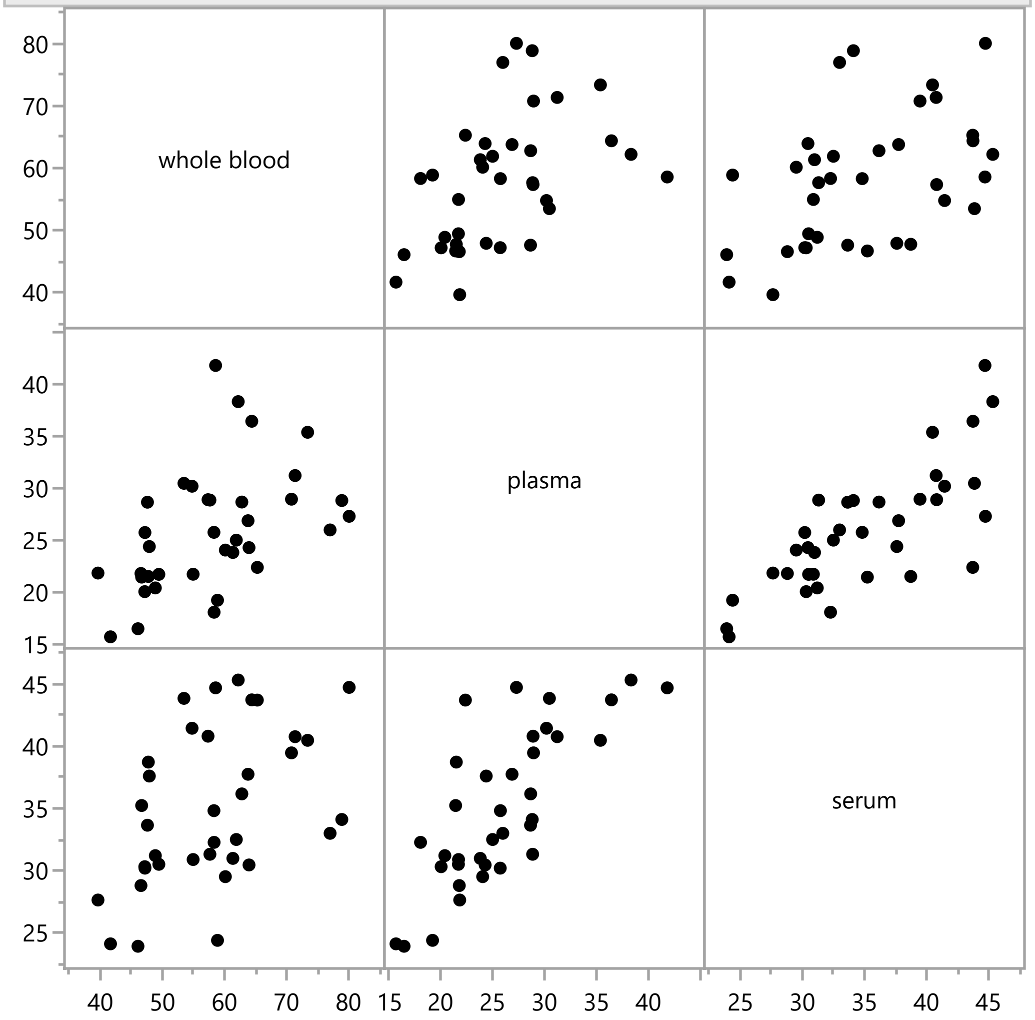

Nonparametric: Spearman's ρ

Variable	by Variable	Spearman ρ	Prob>|ρ|	-.8 -.6 -.4 -.2 0 .2 .4 .6 .8
plasma	whole blood	0.5511	0.0005*	
serum	whole blood	0.5138	0.0014*	
serum	plasma	0.7508	<.0001*	

Multivariate Column 1=glutamine

Correlations

	whole blood	plasma	serum
whole blood	1.0000	0.9665	0.9727
plasma	0.9665	1.0000	0.9916
serum	0.9727	0.9916	1.0000

The correlations are estimated by Row-wise method.

Scatterplot Matrix

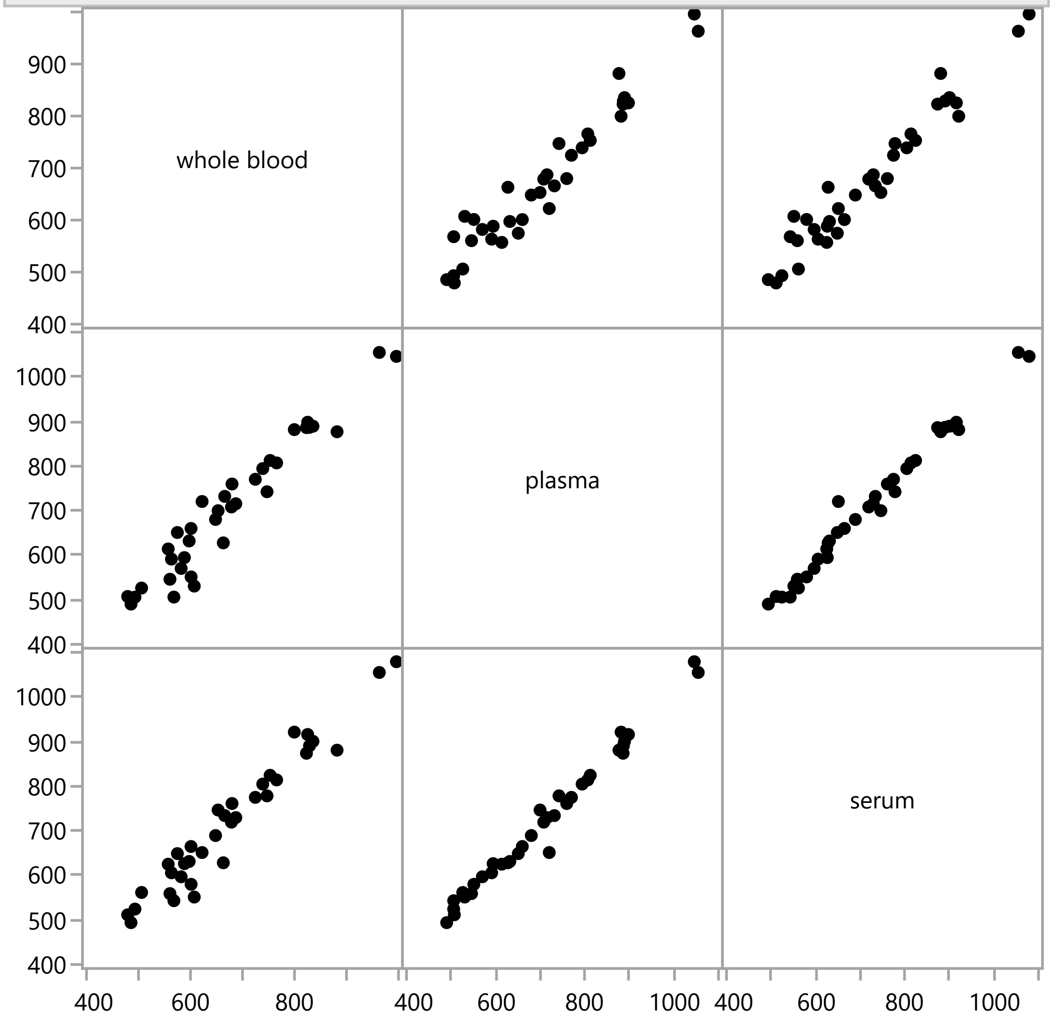

Nonparametric: Spearman's ρ

Variable	by Variable	Spearman ρ	Prob>|ρ|	-.8 -.6 -.4 -.2 0 .2 .4 .6 .8
plasma	whole blood	0.9490	<.0001*	
serum	whole blood	0.9503	<.0001*	
serum	plasma	0.9884	<.0001*	

Multivariate Column 1=glycine

Correlations

	whole blood	plasma	serum
whole blood	1.0000	0.9631	0.9709
plasma	0.9631	1.0000	0.9926
serum	0.9709	0.9926	1.0000

The correlations are estimated by Row-wise method.

Scatterplot Matrix

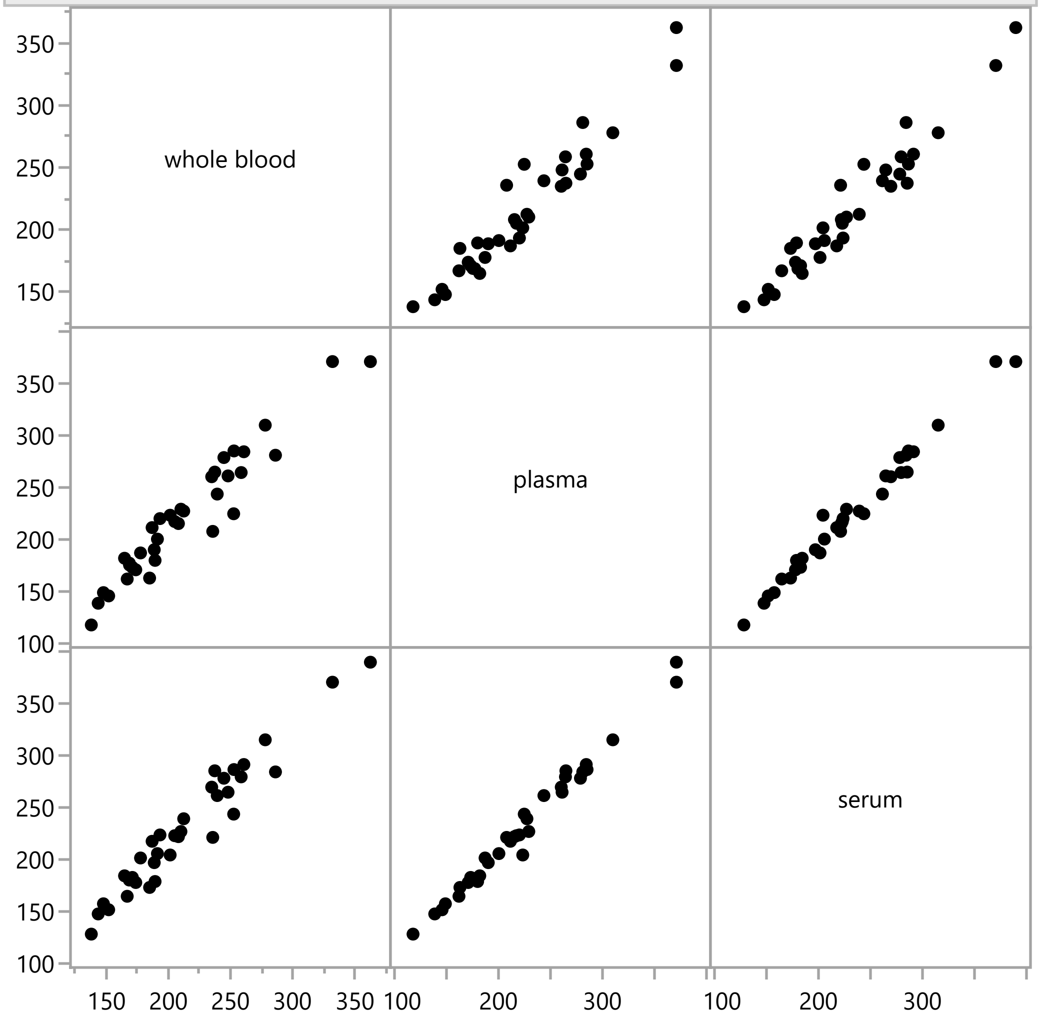

Nonparametric: Spearman's ρ

Variable	by Variable	Spearman ρ	Prob>|ρ|	-.8 -.6 -.4 -.2 0 .2 .4 .6 .8
plasma	whole blood	0.9542	<.0001*	
serum	whole blood	0.9539	<.0001*	
serum	plasma	0.9884	<.0001*	

Multivariate Column 1=histidine

Correlations

	whole blood	plasma	serum
whole blood	1.0000	0.7926	0.8249
plasma	0.7926	1.0000	0.9277
serum	0.8249	0.9277	1.0000

The correlations are estimated by Row-wise method.

Scatterplot Matrix

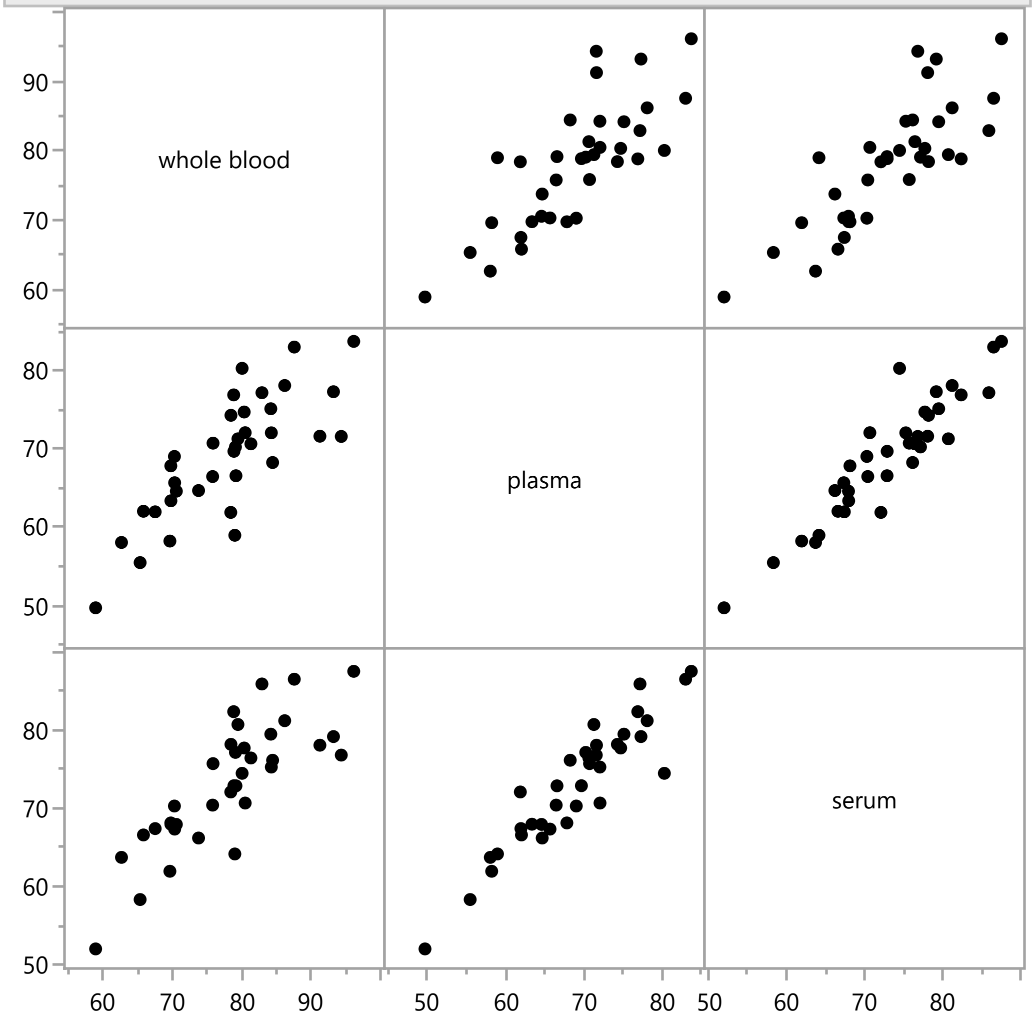

Nonparametric: Spearman's ρ

Variable	by Variable	Spearman ρ	Prob>|ρ|
plasma	whole blood	0.8106	<.0001*
serum	whole blood	0.8175	<.0001*
serum	plasma	0.9017	<.0001*

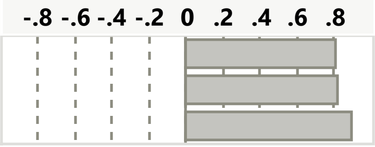

Multivariate Column 1=homocystine

Correlations

	whole blood	plasma	serum
whole blood	1.0000	0.7968	0.7450
plasma	0.7968	1.0000	0.9720
serum	0.7450	0.9720	1.0000

The correlations are estimated by Row-wise method.

Scatterplot Matrix

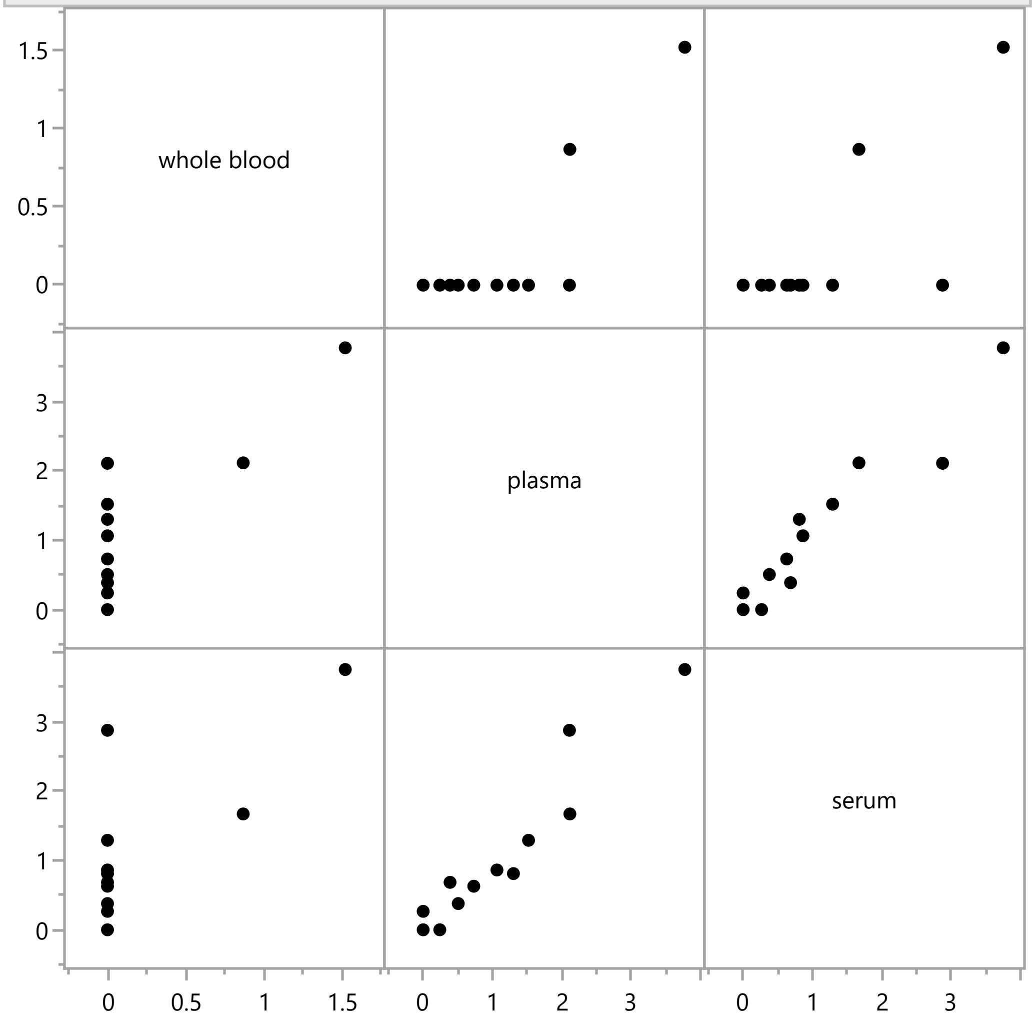

Nonparametric: Spearman's ρ

Variable	by Variable	Spearman ρ	Prob>|ρ|
plasma	whole blood	0.5028	0.0018*
serum	whole blood	0.4885	0.0025*
serum	plasma	0.9227	<.0001*

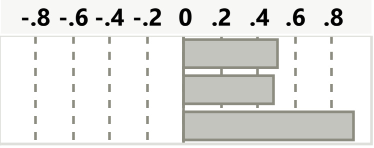

Multivariate Column 1=hydroxylysine

Correlations

	whole blood	plasma	serum
whole blood	1.0000	0.1831	-0.1396
plasma	0.1831	1.0000	0.5103
serum	-0.1396	0.5103	1.0000

The correlations are estimated by Row-wise method.

Scatterplot Matrix

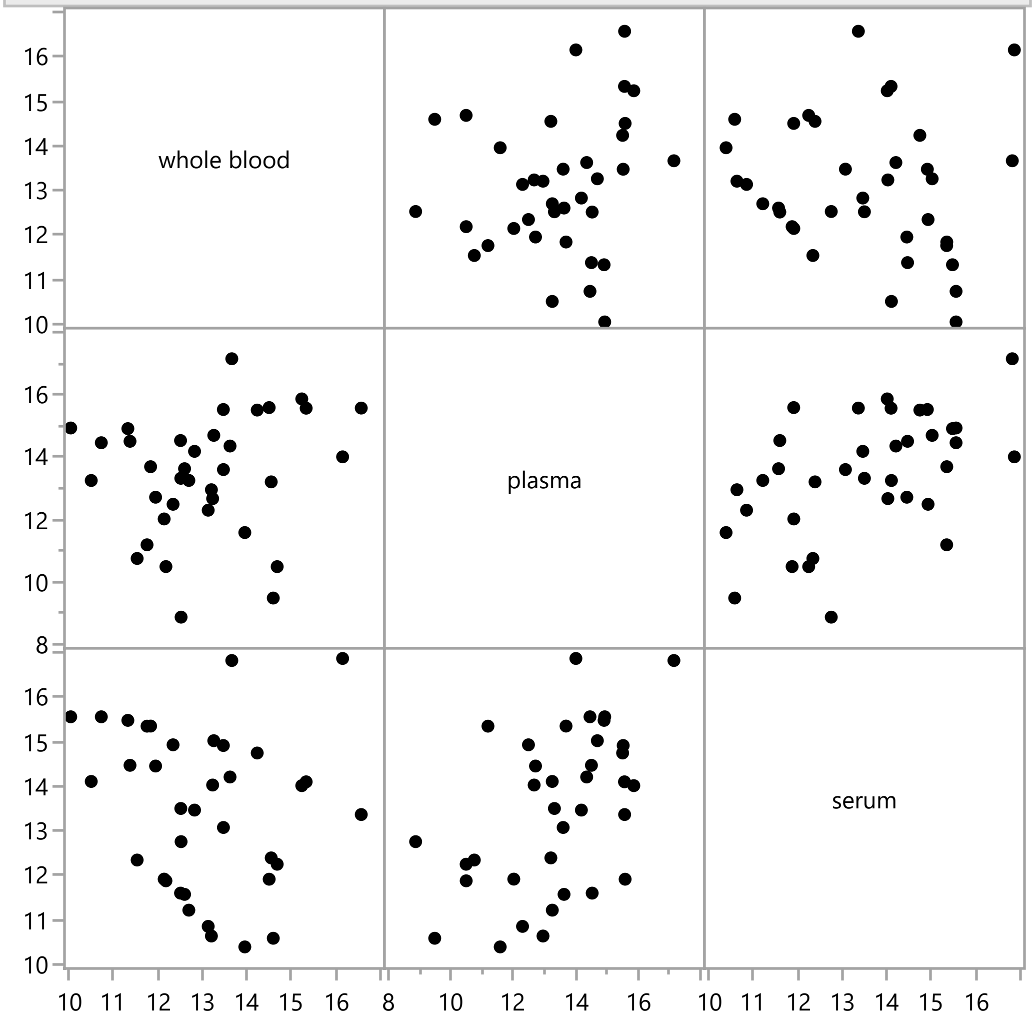

Nonparametric: Spearman's ρ

Variable	by Variable	Spearman ρ	Prob>|ρ|	-.8 -.6 -.4 -.2 0 .2 .4 .6 .8
plasma	whole blood	0.2227	0.1918	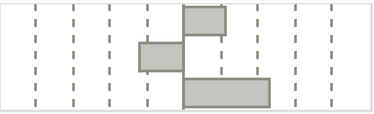
serum	whole blood	-0.2376	0.1629	
serum	plasma	0.4691	0.0039*	

Multivariate Column 1=hydroxyproline

Correlations

	whole blood	plasma	serum
whole blood	1.0000	0.9788	0.9857
plasma	0.9788	1.0000	0.9932
serum	0.9857	0.9932	1.0000

The correlations are estimated by Row-wise method.

Scatterplot Matrix

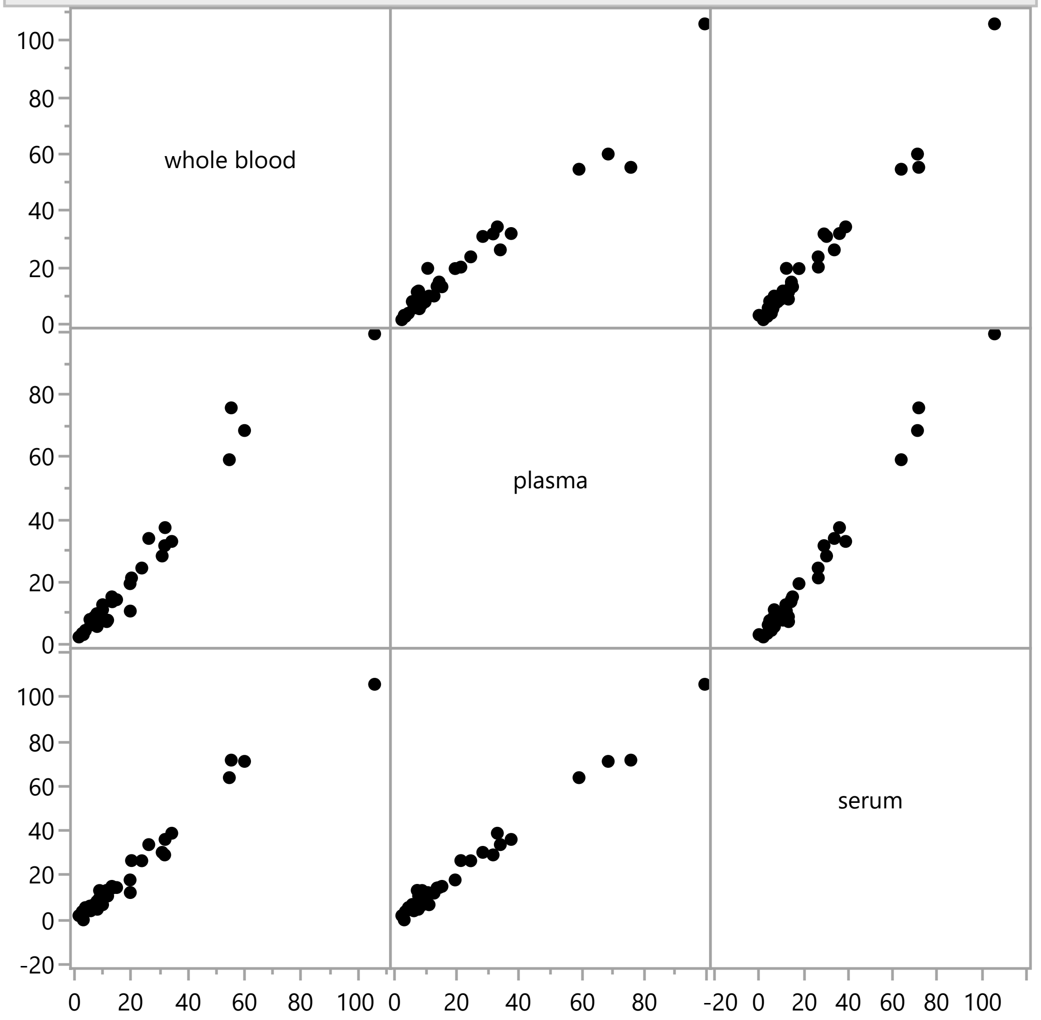

Nonparametric: Spearman's ρ

Variable	by Variable	Spearman ρ	Prob>|ρ|	-.8 -.6 -.4 -.2 0 .2 .4 .6 .8
plasma	whole blood	0.9315	<.0001*	
serum	whole blood	0.9676	<.0001*	
serum	plasma	0.9284	<.0001*	

Multivariate Column 1=isoleucine

Correlations

	whole blood	plasma	serum
whole blood	1.0000	0.9411	0.9408
plasma	0.9411	1.0000	0.9820
serum	0.9408	0.9820	1.0000

The correlations are estimated by Row-wise method.

Scatterplot Matrix

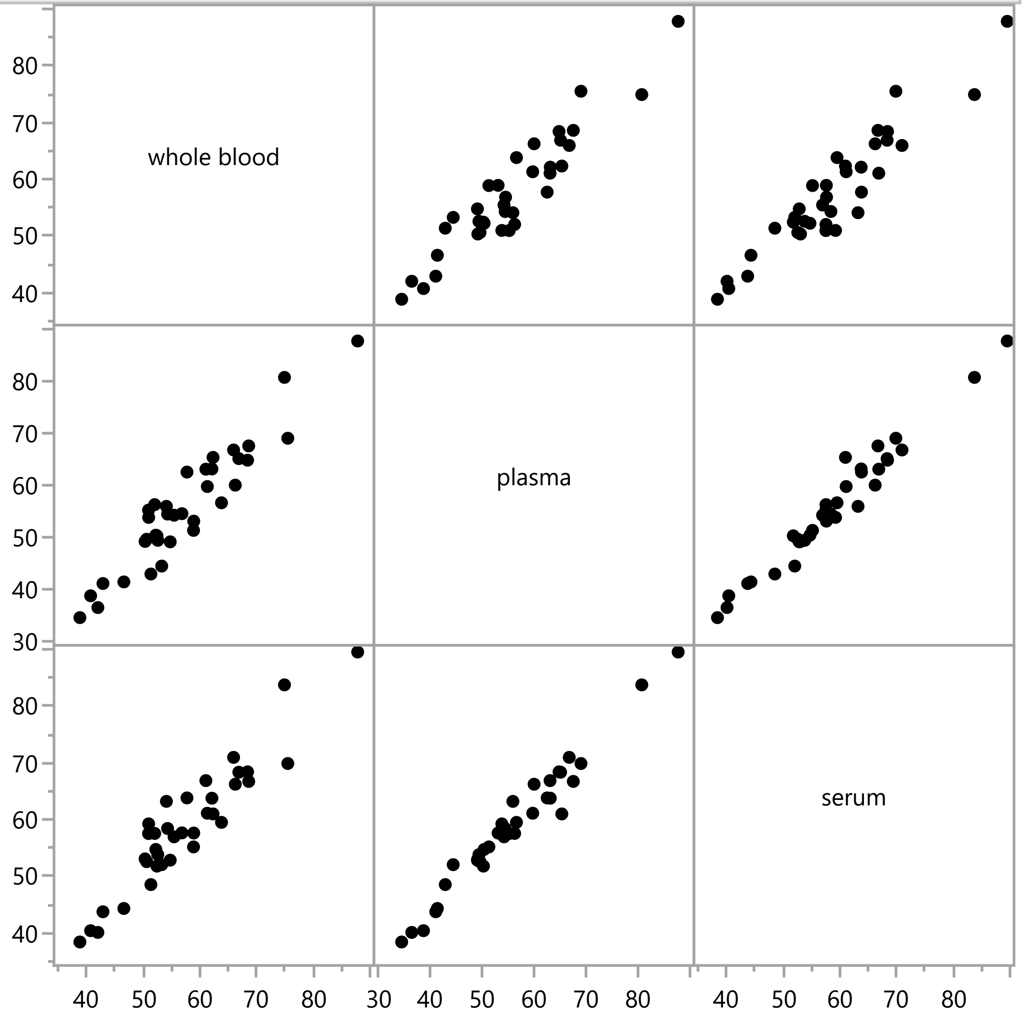

Nonparametric: Spearman's ρ

Variable	by Variable	Spearman ρ	Prob>|ρ|	-.8 -.6 -.4 -.2 0 .2 .4 .6 .8
plasma	whole blood	0.8940	<.0001*	
serum	whole blood	0.8950	<.0001*	
serum	plasma	0.9665	<.0001*	

Multivariate Column 1=leucine

Correlations

	whole blood	plasma	serum
whole blood	1.0000	0.9306	0.9374
plasma	0.9306	1.0000	0.9831
serum	0.9374	0.9831	1.0000

The correlations are estimated by Row-wise method.

Scatterplot Matrix

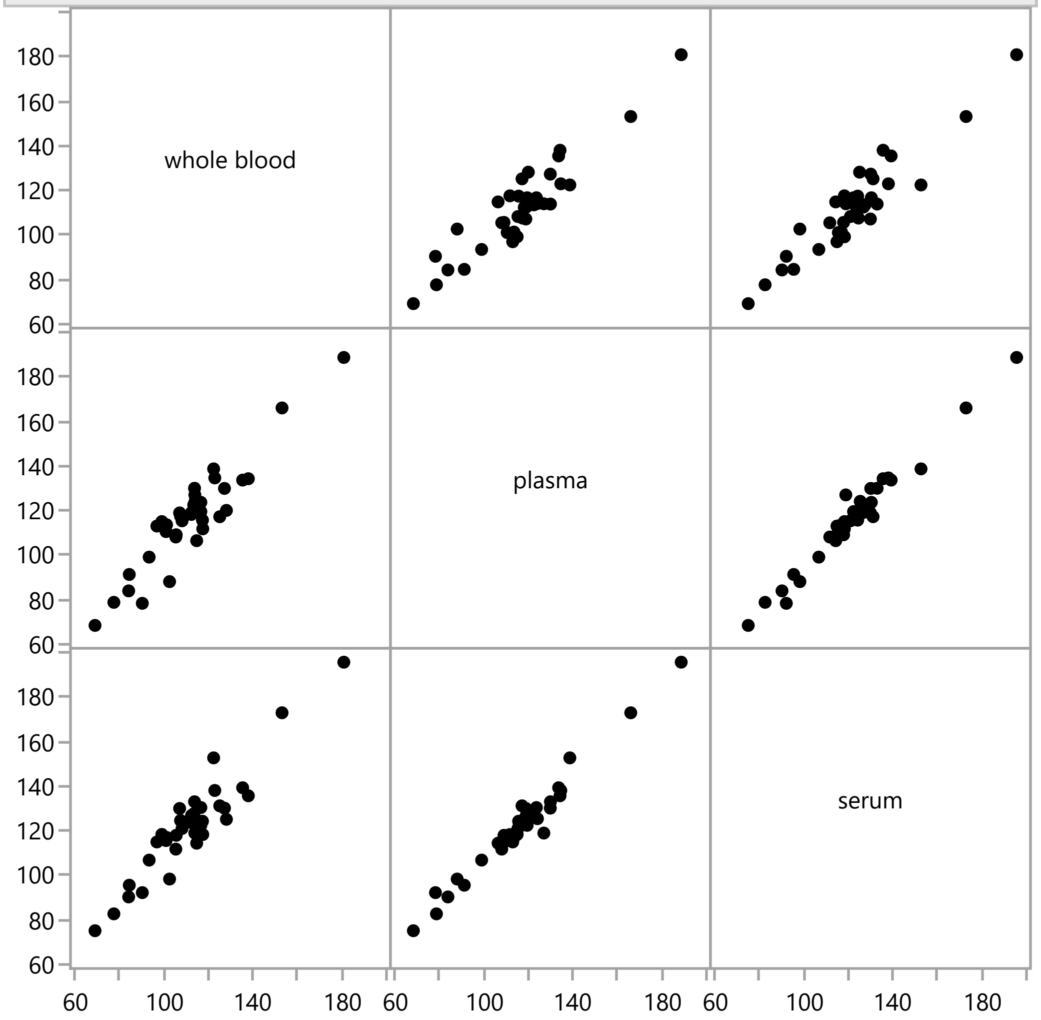

Nonparametric: Spearman's ρ

Variable	by Variable	Spearman ρ	Prob>|ρ|	-.8 -.6 -.4 -.2 0 .2 .4 .6 .8
plasma	whole blood	0.8345	<.0001*	
serum	whole blood	0.8546	<.0001*	
serum	plasma	0.9480	<.0001*	

Multivariate Column 1=lysine

Correlations

	whole blood	plasma	serum
whole blood	1.0000	0.8842	0.8984
plasma	0.8842	1.0000	0.9949
serum	0.8984	0.9949	1.0000

The correlations are estimated by Row-wise method.

Scatterplot Matrix

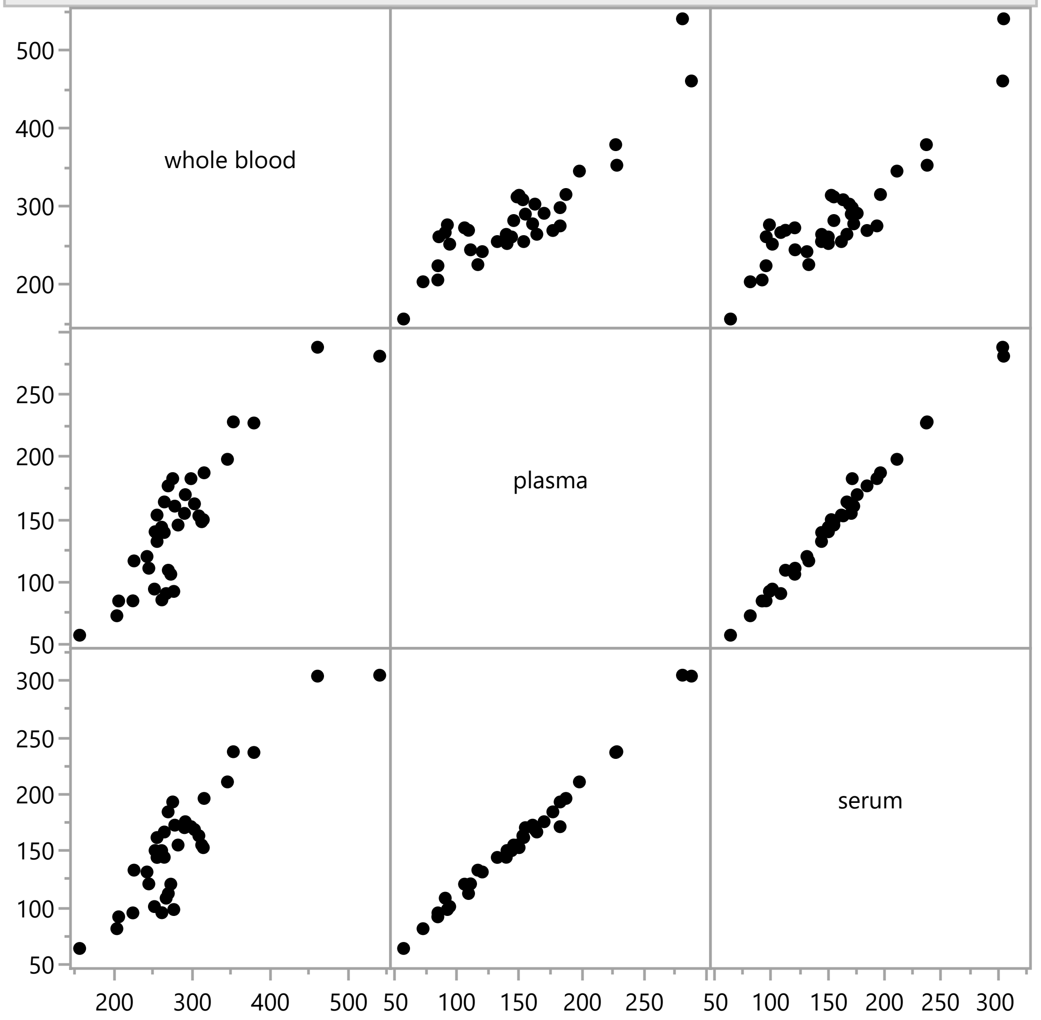

Nonparametric: Spearman's ρ

Variable	by Variable	Spearman ρ	Prob>|ρ|	-.8 -.6 -.4 -.2 0 .2 .4 .6 .8
plasma	whole blood	0.8059	<.0001*	
serum	whole blood	0.8088	<.0001*	
serum	plasma	0.9925	<.0001*	

Multivariate Column 1=methionine

Correlations

	whole blood	plasma	serum
whole blood	1.0000	0.8392	0.8693
plasma	0.8392	1.0000	0.9559
serum	0.8693	0.9559	1.0000

The correlations are estimated by Row-wise method.

Scatterplot Matrix

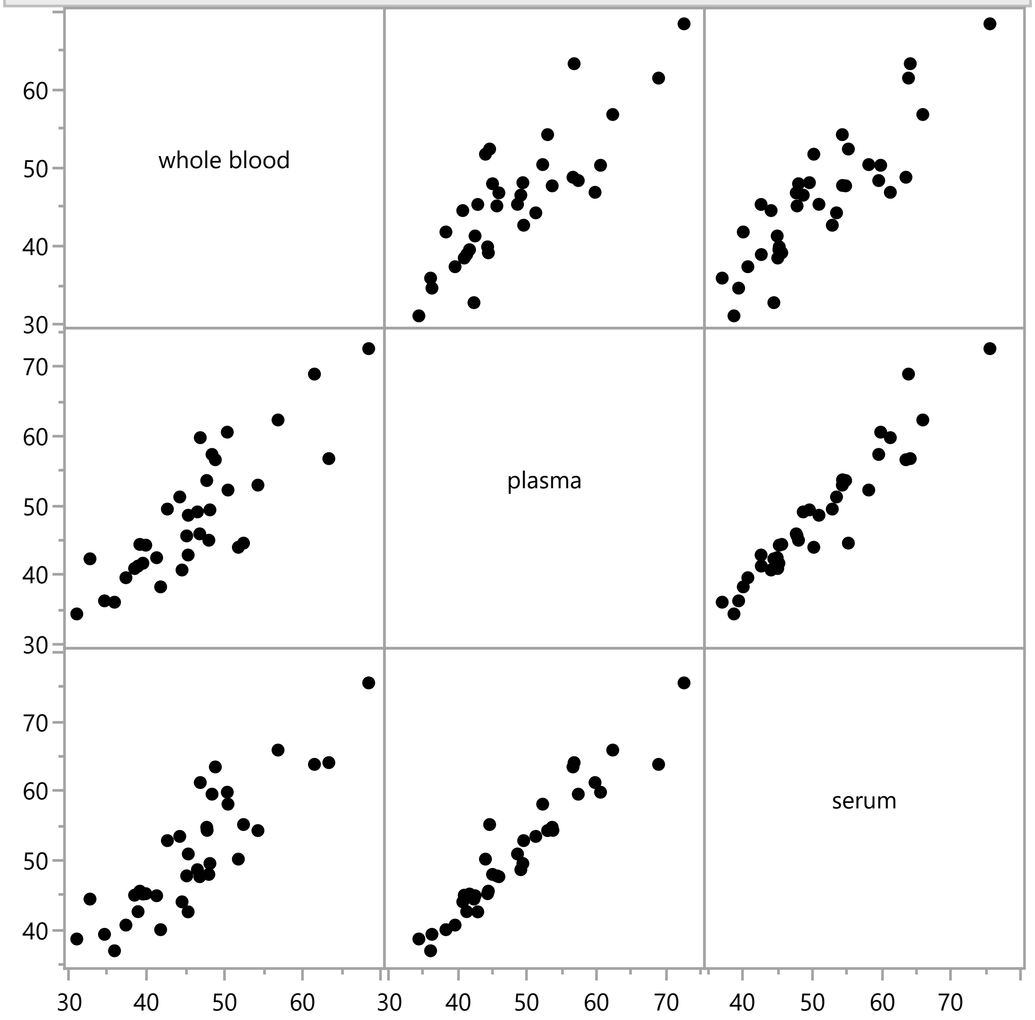

Nonparametric: Spearman's ρ

Variable	by Variable	Spearman ρ	Prob>|ρ|	-.8 -.6 -.4 -.2 0 .2 .4 .6 .8
plasma	whole blood	0.8206	<.0001*	
serum	whole blood	0.8713	<.0001*	
serum	plasma	0.9550	<.0001*	

Multivariate Column 1=ornithine

Correlations

	whole blood	plasma	serum
whole blood	1.0000	0.8504	0.8325
plasma	0.8504	1.0000	0.9849
serum	0.8325	0.9849	1.0000

The correlations are estimated by Row-wise method.

Scatterplot Matrix

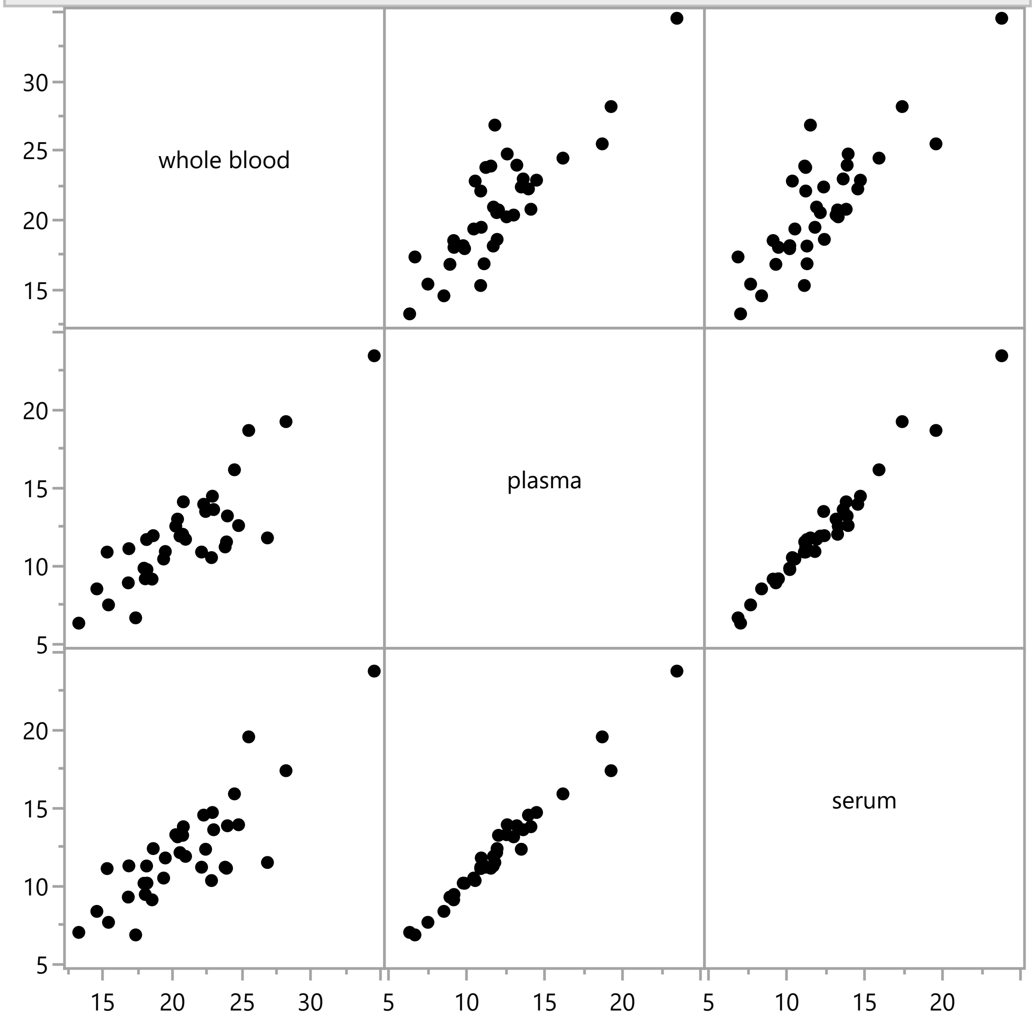

Nonparametric: Spearman's ρ

Variable	by Variable	Spearman ρ	Prob>|ρ|
plasma	whole blood	0.7905	<.0001*
serum	whole blood	0.7663	<.0001*
serum	plasma	0.9793	<.0001*

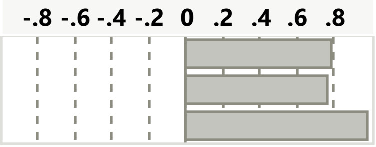

Multivariate Column 1=phenylalanine

Correlations

	whole blood	plasma	serum
whole blood	1.0000	0.9224	0.9443
plasma	0.9224	1.0000	0.9651
serum	0.9443	0.9651	1.0000

The correlations are estimated by Row-wise method.

Scatterplot Matrix

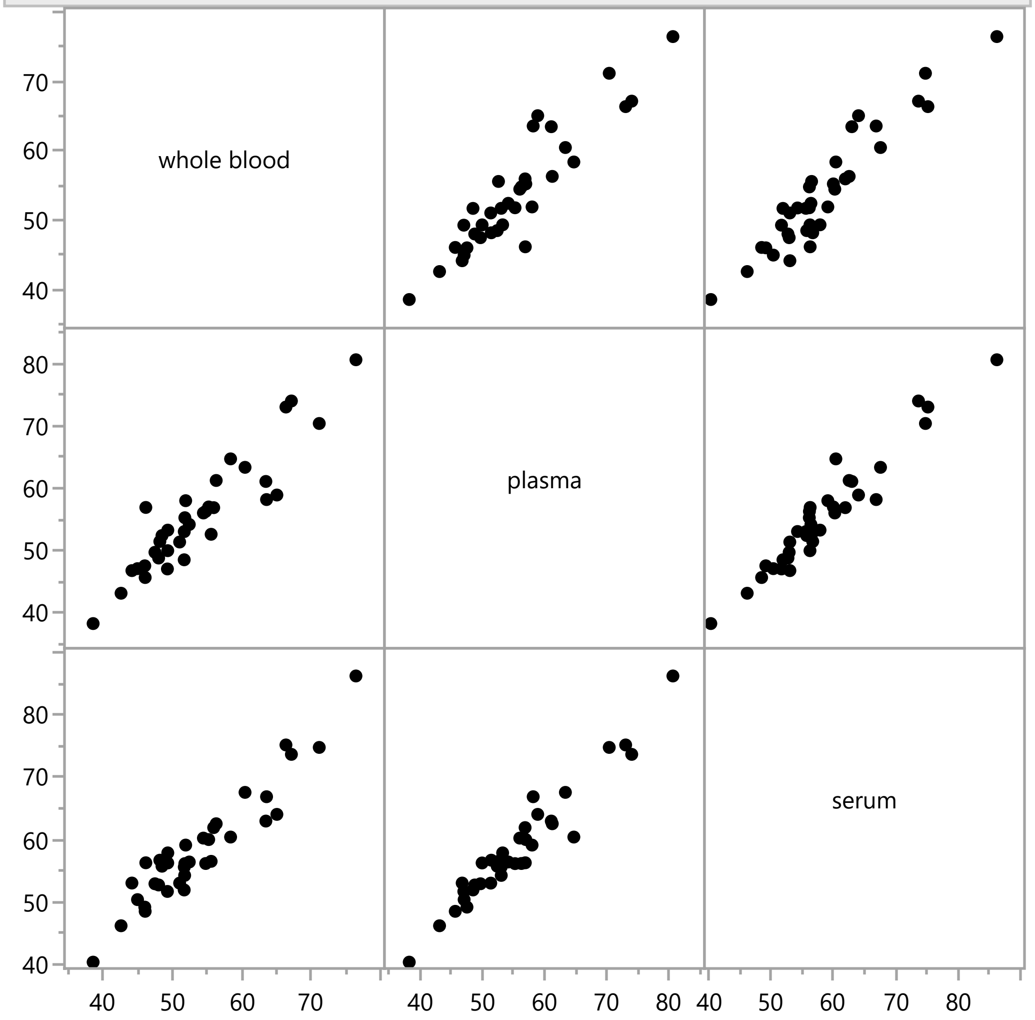

Nonparametric: Spearman's ρ

Variable	by Variable	Spearman ρ	Prob>|ρ|
plasma	whole blood	0.9107	<.0001*
serum	whole blood	0.9048	<.0001*
serum	plasma	0.9423	<.0001*

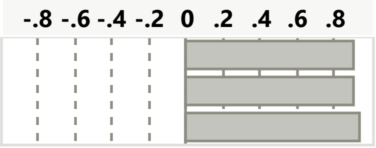

Multivariate Column 1=phosphoethanolamine

Correlations

	whole blood	plasma	serum
whole blood	1.0000	0.2399	0.1461
plasma	0.2399	1.0000	0.6654
serum	0.1461	0.6654	1.0000

The correlations are estimated by Row-wise method.

Scatterplot Matrix

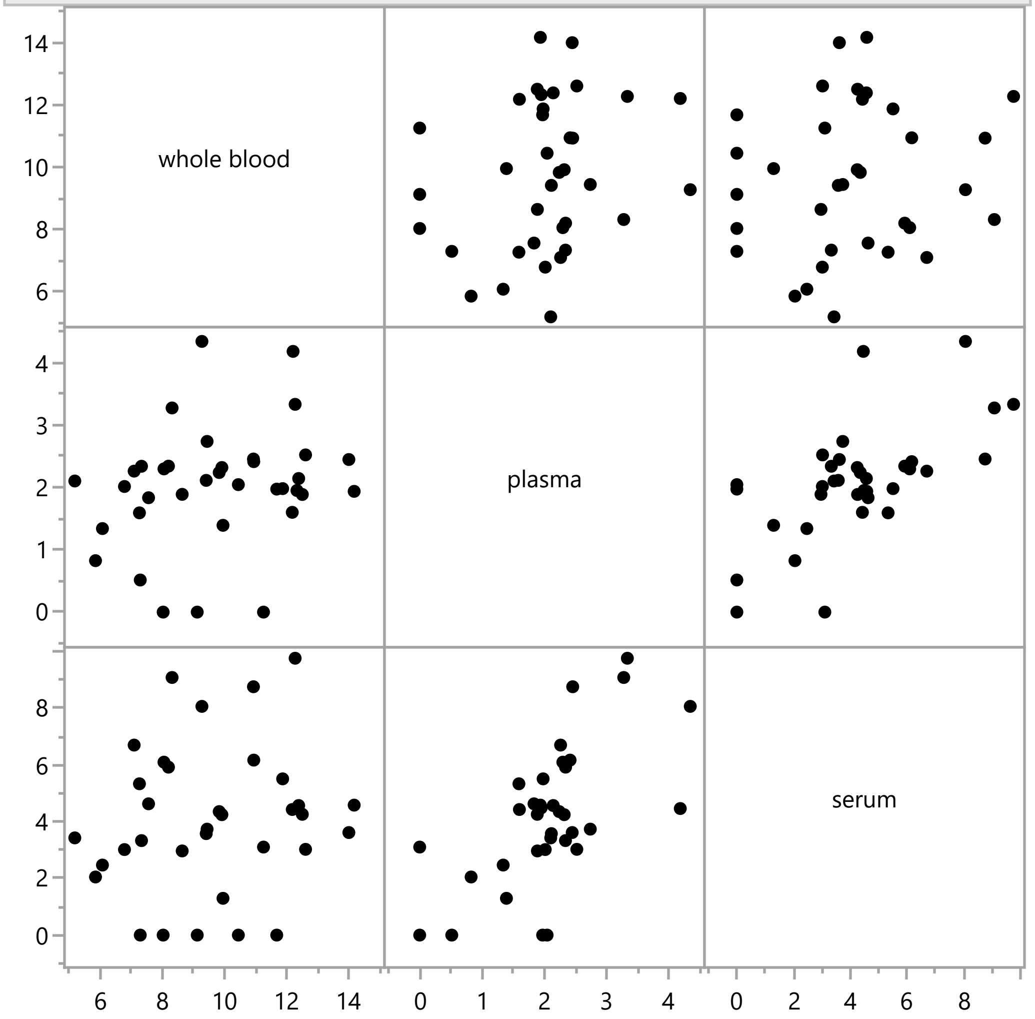

Nonparametric: Spearman's ρ

Variable	by Variable	Spearman ρ	Prob>|ρ|	-.8 -.6 -.4 -.2 0 .2 .4 .6 .8
plasma	whole blood	0.2570	0.1303	
serum	whole blood	0.1843	0.2820	
serum	plasma	0.5953	0.0001*	

Multivariate Column 1=phosphoserine

Correlations

	whole blood	plasma	serum
whole blood	1.0000	0.7118	0.3931
plasma	0.7118	1.0000	0.5428
serum	0.3931	0.5428	1.0000

The correlations are estimated by Row-wise method.

Scatterplot Matrix

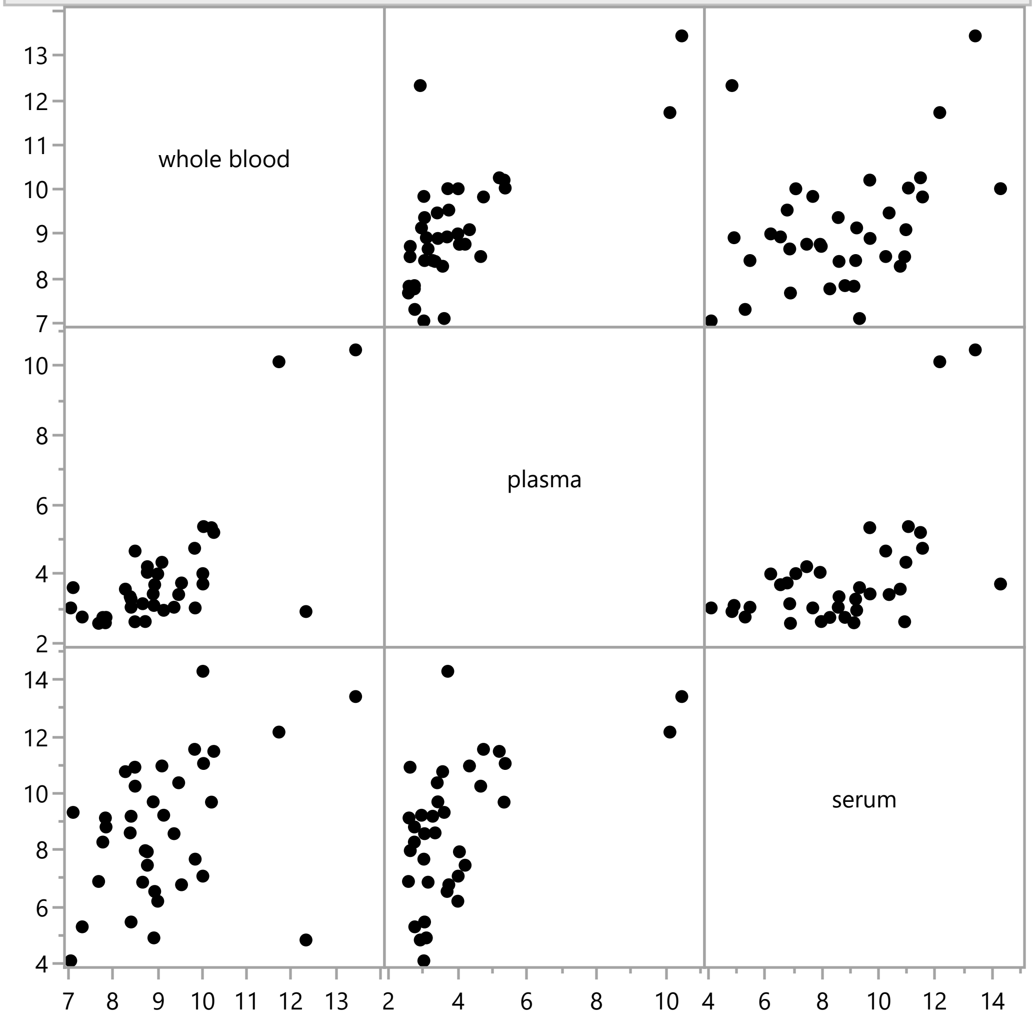

Nonparametric: Spearman's ρ

Variable	by Variable	Spearman ρ	Prob>|ρ|	-.8 -.6 -.4 -.2 0 .2 .4 .6 .8
plasma	whole blood	0.6052	<.0001*	
serum	whole blood	0.3578	0.0321*	
serum	plasma	0.4824	0.0029*	

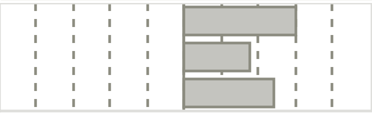

Multivariate Column 1=proline

Correlations

	whole blood	plasma	serum
whole blood	1.0000	0.9660	0.9667
plasma	0.9660	1.0000	0.9942
serum	0.9667	0.9942	1.0000

The correlations are estimated by Row-wise method.

Scatterplot Matrix

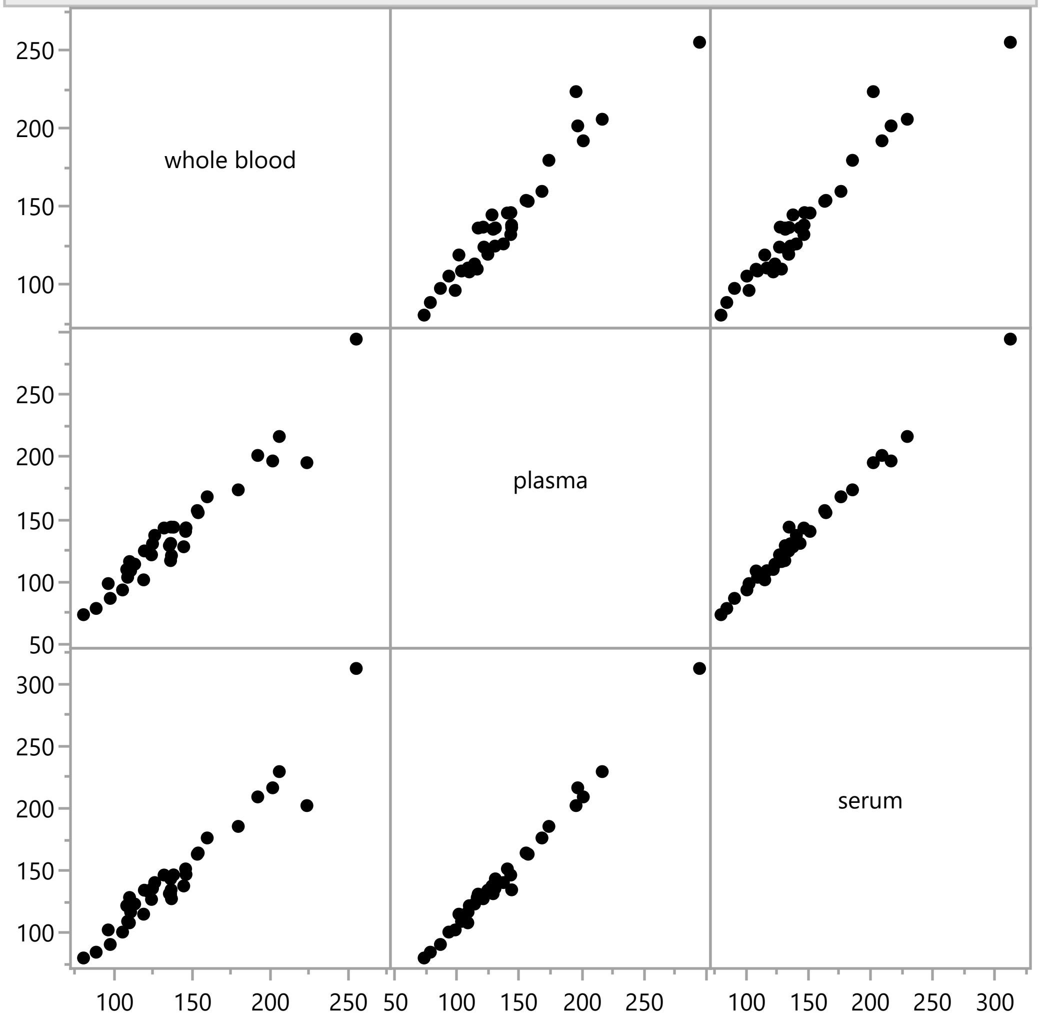

Nonparametric: Spearman's ρ

Variable	by Variable	Spearman ρ	Prob>|ρ|	-.8 -.6 -.4 -.2 0 .2 .4 .6 .8
plasma	whole blood	0.9483	<.0001*	
serum	whole blood	0.9529	<.0001*	
serum	plasma	0.9825	<.0001*	

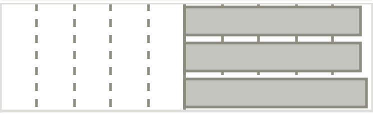

Multivariate Column 1=serine

Correlations

	whole blood	plasma	serum
whole blood	1.0000	0.9168	0.9314
plasma	0.9168	1.0000	0.9821
serum	0.9314	0.9821	1.0000

The correlations are estimated by Row-wise method.

Scatterplot Matrix

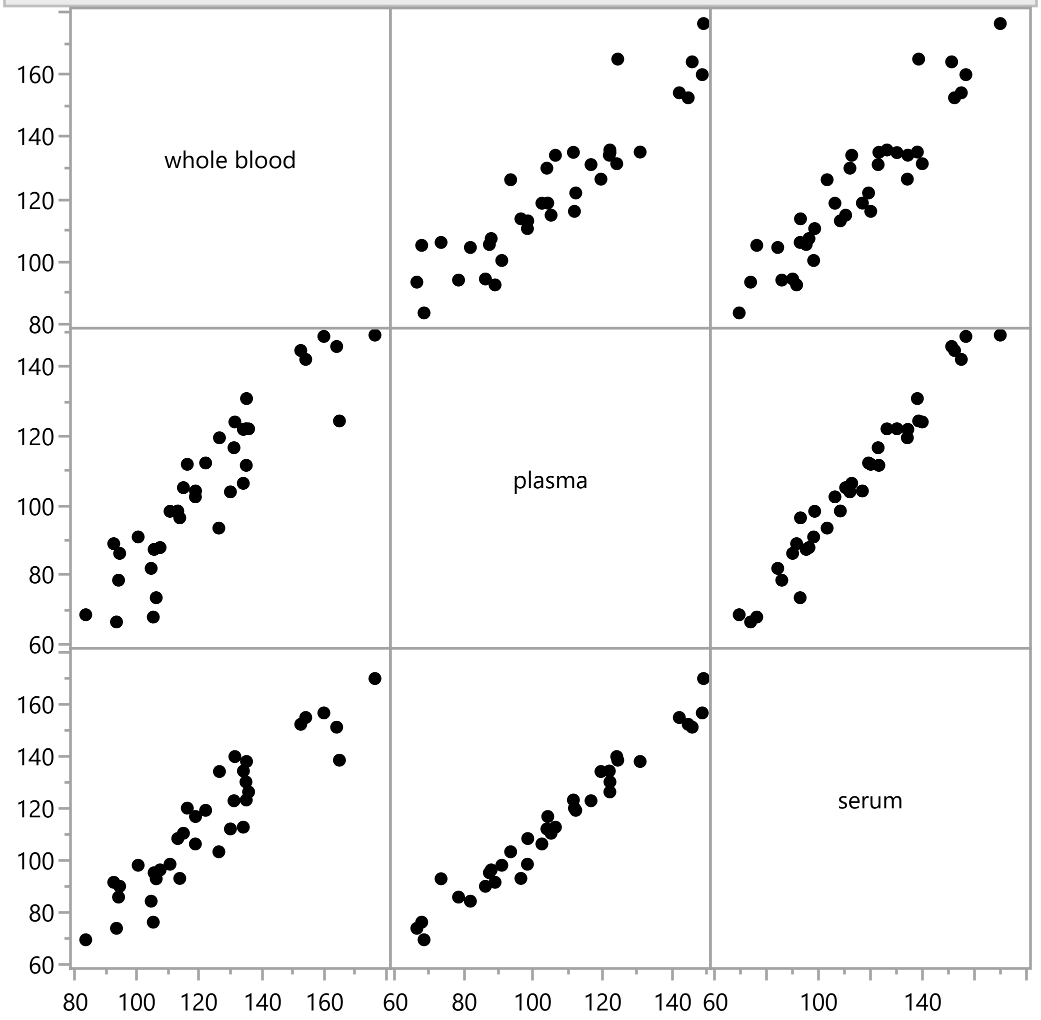

Nonparametric: Spearman's ρ

Variable	by Variable	Spearman ρ	Prob>|ρ|
plasma	whole blood	0.9344	<.0001*
serum	whole blood	0.9421	<.0001*
serum	plasma	0.9840	<.0001*

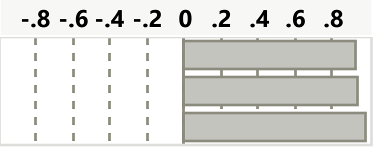

Multivariate Column 1=taurine

Correlations

	whole blood	plasma	serum
whole blood	1.0000	0.6258	0.6990
plasma	0.6258	1.0000	0.4639
serum	0.6990	0.4639	1.0000

The correlations are estimated by Row-wise method.

Scatterplot Matrix

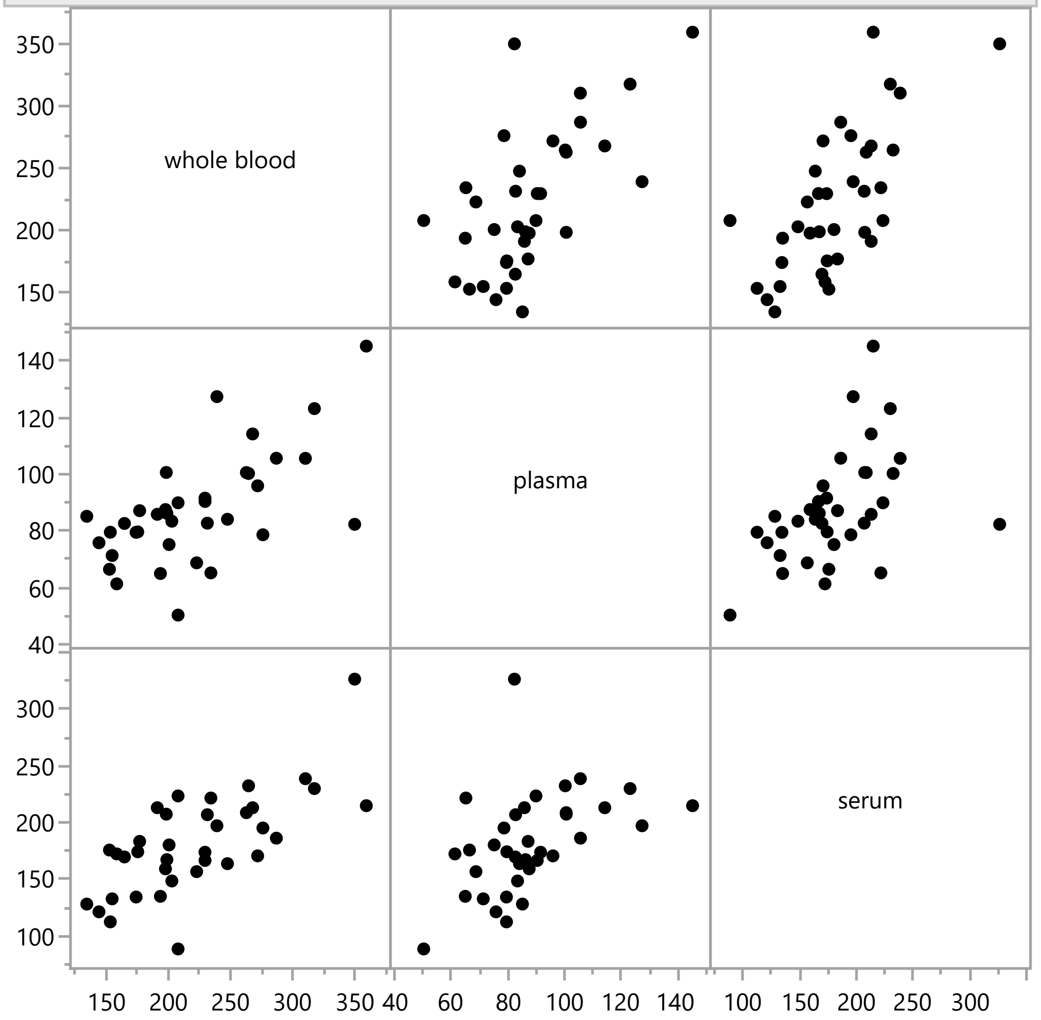

Nonparametric: Spearman's ρ

Variable	by Variable	Spearman ρ	Prob>|ρ|	-.8 -.6 -.4 -.2 0 .2 .4 .6 .8
plasma	whole blood	0.5586	0.0004*	
serum	whole blood	0.6484	<.0001*	
serum	plasma	0.5241	0.0010*	

Multivariate Column 1=threonine

Correlations

	whole blood	plasma	serum
whole blood	1.0000	0.9629	0.9701
plasma	0.9629	1.0000	0.9934
serum	0.9701	0.9934	1.0000

The correlations are estimated by Row-wise method.

Scatterplot Matrix

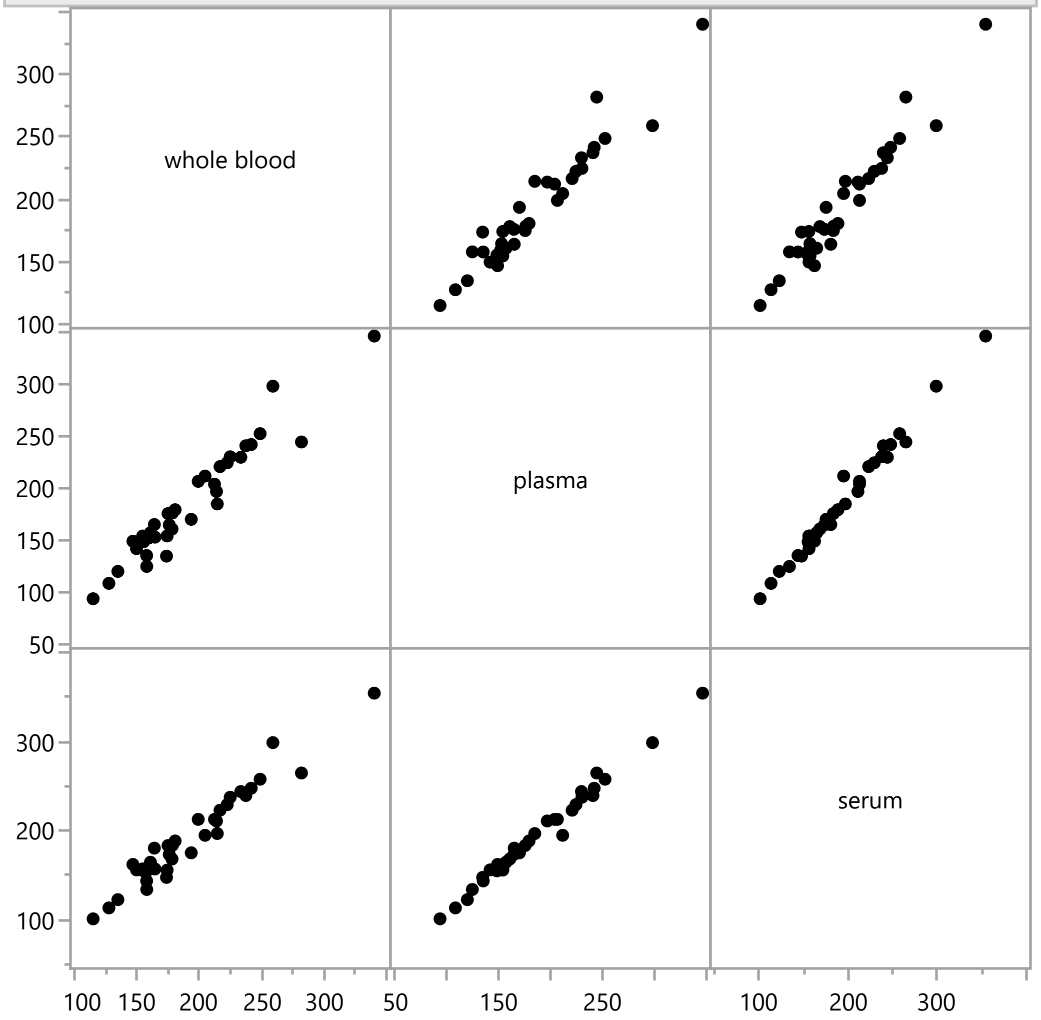

Nonparametric: Spearman's ρ

Variable	by Variable	Spearman ρ	Prob>|ρ|
plasma	whole blood	0.9622	<.0001*
serum	whole blood	0.9491	<.0001*
serum	plasma	0.9899	<.0001*

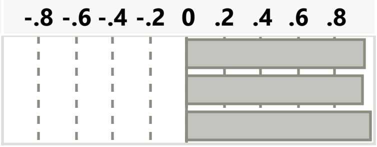

Multivariate Column 1=tryptophan

Correlations

	whole blood	plasma	serum
whole blood	1.0000	0.9404	0.9340
plasma	0.9404	1.0000	0.9916
serum	0.9340	0.9916	1.0000

The correlations are estimated by Row-wise method.

Scatterplot Matrix

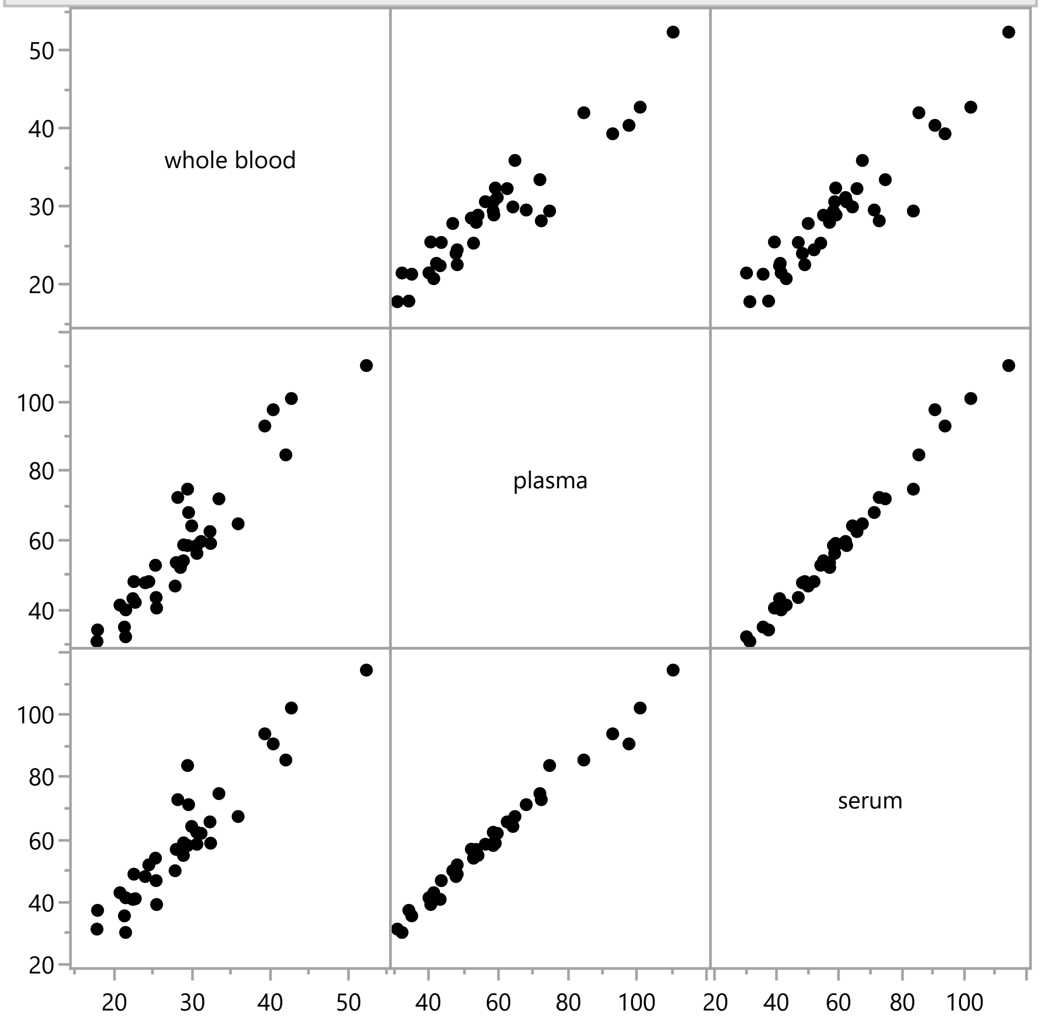

Nonparametric: Spearman's ρ

Variable	by Variable	Spearman ρ	Prob>|ρ|	-.8 -.6 -.4 -.2 0 .2 .4 .6 .8
plasma	whole blood	0.9176	<.0001*	
serum	whole blood	0.9184	<.0001*	
serum	plasma	0.9905	<.0001*	

Multivariate Column 1=tyrosine

Correlations

	whole blood	plasma	serum
whole blood	1.0000	0.8018	0.8051
plasma	0.8018	1.0000	0.9815
serum	0.8051	0.9815	1.0000

The correlations are estimated by Row-wise method.

Scatterplot Matrix

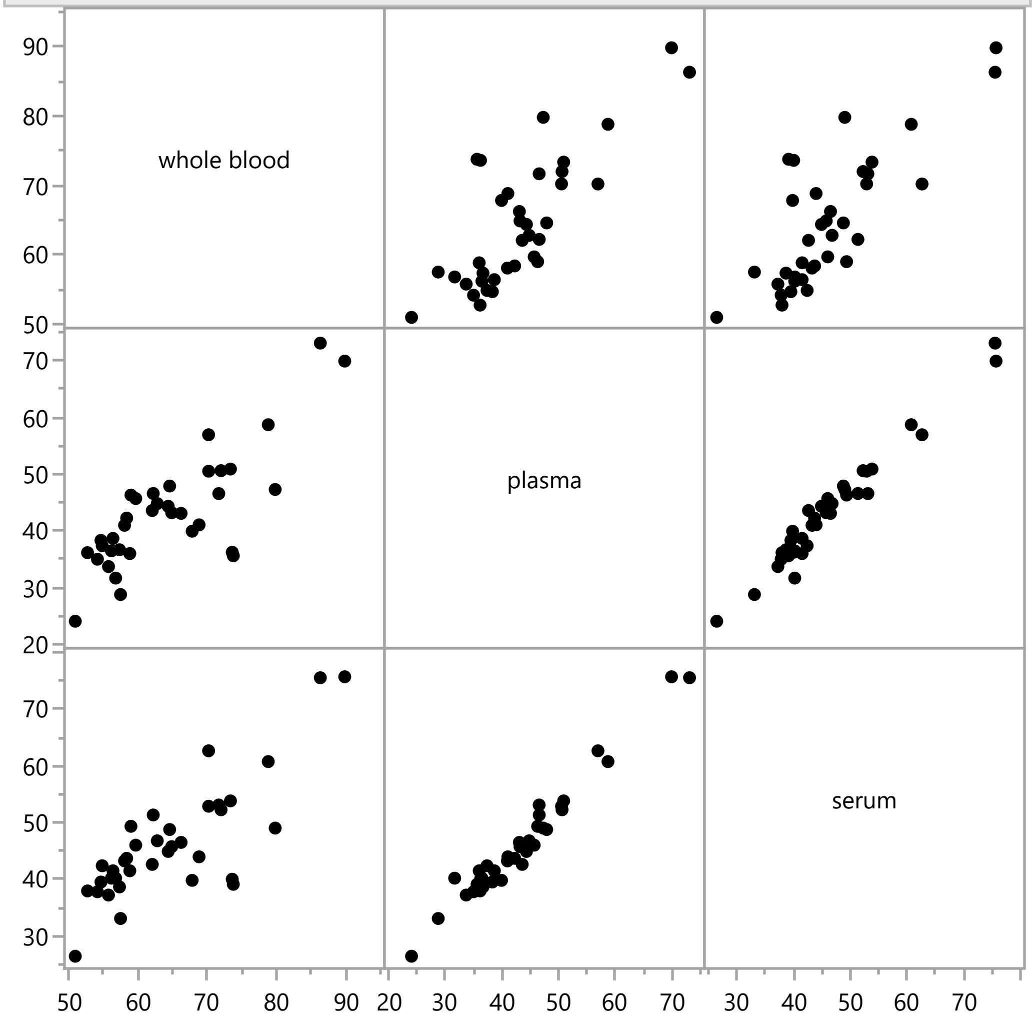

Nonparametric: Spearman's ρ

Variable	by Variable	Spearman ρ	Prob>|ρ|	-.8 -.6 -.4 -.2 0 .2 .4 .6 .8
plasma	whole blood	0.7161	<.0001*	
serum	whole blood	0.7249	<.0001*	
serum	plasma	0.9554	<.0001*	

Multivariate Column 1=urea

Correlations

	whole blood	plasma	serum
whole blood	1.0000	0.9706	0.9790
plasma	0.9706	1.0000	0.9924
serum	0.9790	0.9924	1.0000

The correlations are estimated by Row-wise method.

Scatterplot Matrix

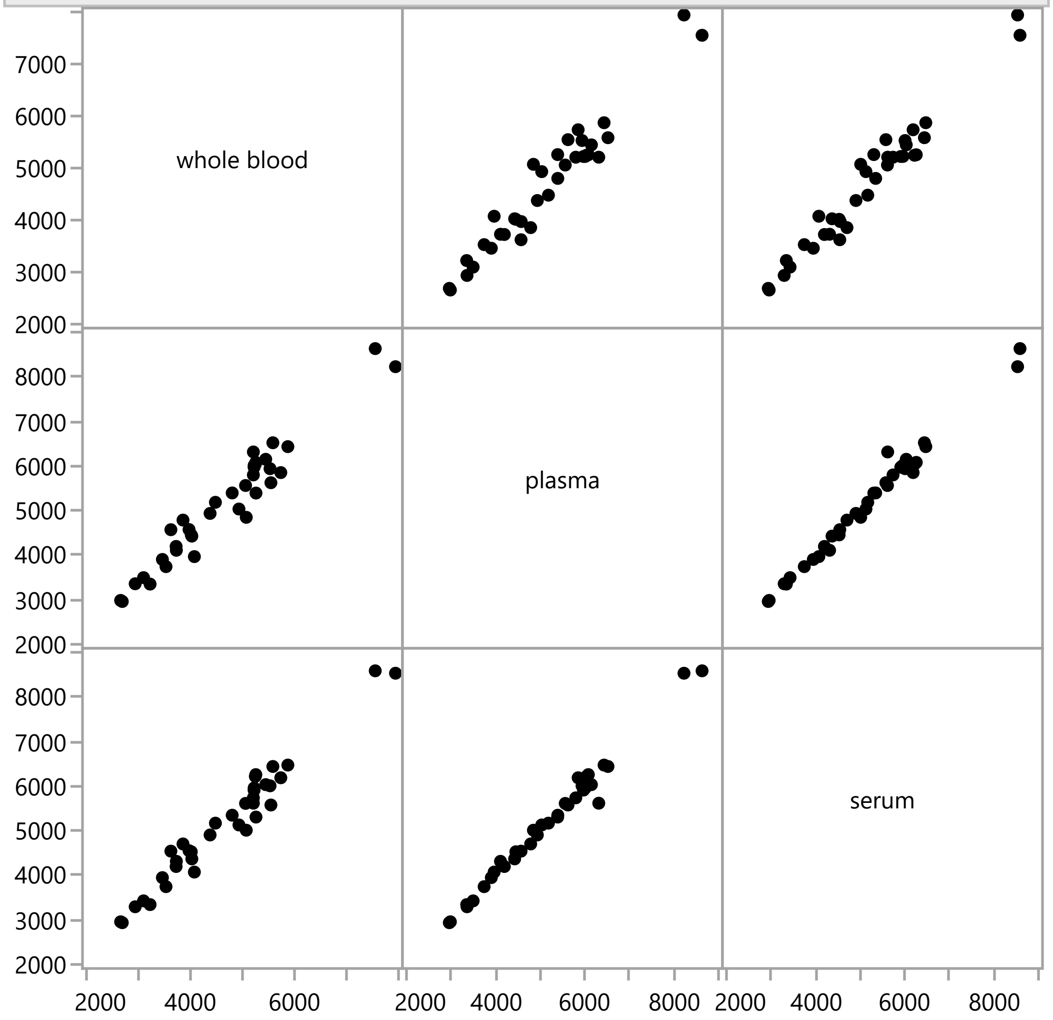

Nonparametric: Spearman's ρ

Variable	by Variable	Spearman ρ	Prob>|ρ|	-.8 -.6 -.4 -.2 0 .2 .4 .6 .8
plasma	whole blood	0.9356	<.0001*	
serum	whole blood	0.9514	<.0001*	
serum	plasma	0.9848	<.0001*	

Multivariate Column 1=valine

Correlations

	whole blood	plasma	serum
whole blood	1.0000	0.8302	0.8569
plasma	0.8302	1.0000	0.9811
serum	0.8569	0.9811	1.0000

The correlations are estimated by Row-wise method.

Scatterplot Matrix

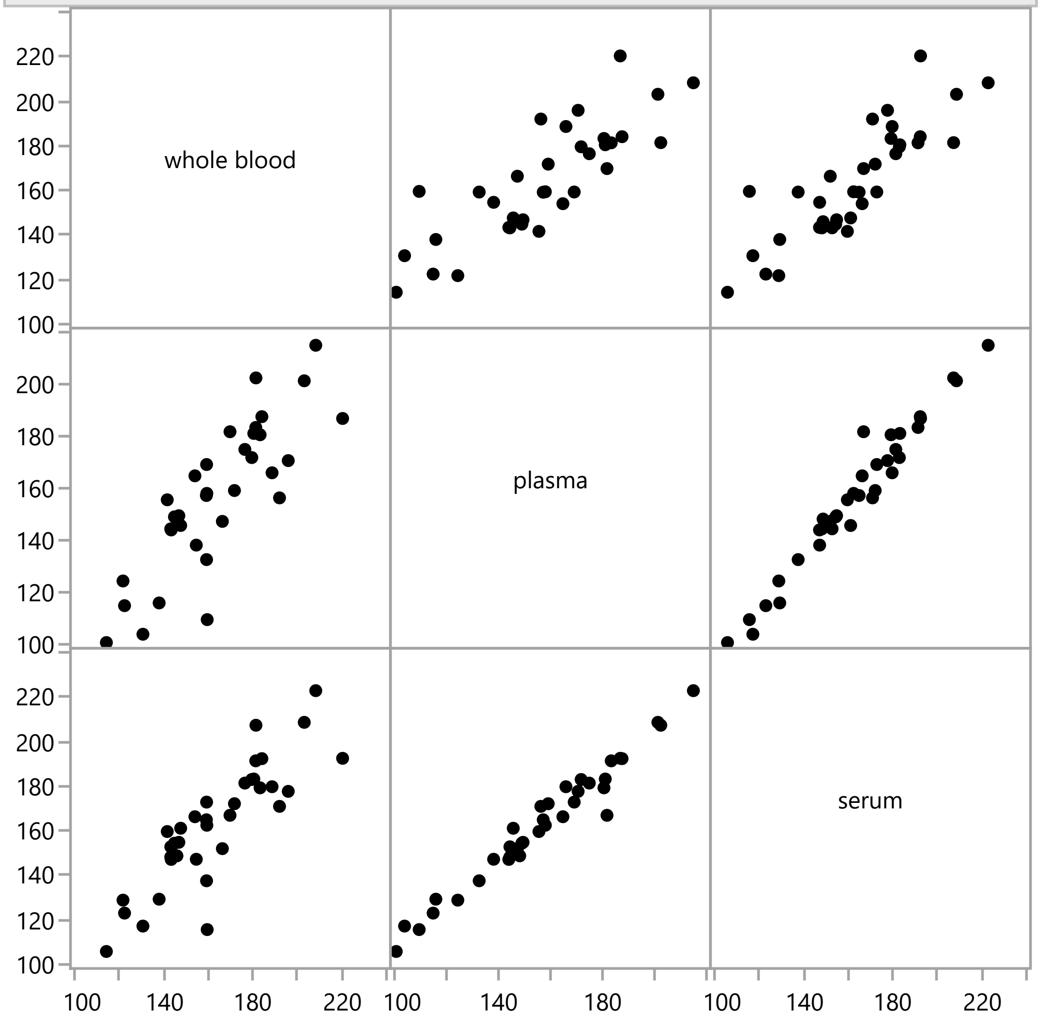

Nonparametric: Spearman's ρ

Variable	by Variable	Spearman ρ	Prob>|ρ|	-.8 -.6 -.4 -.2 0 .2 .4 .6 .8
plasma	whole blood	0.8293	<.0001*	
serum	whole blood	0.8564	<.0001*	
serum	plasma	0.9753	<.0001*	
