# Supplementary material for: Analytical Validation of an Assay for Concurrent Measurement of Amino Acids in Dog Serum and Comparison of Amino Acid Concentrations between Whole Blood, Plasma, and Serum from Dogs
Source: Metabolites. 2022 Sep 22;12(10):891. doi: 10.3390/metabo12100891 (PMC9608751; doi:10.3390/metabo12100891)
Supplement: Supplementary file 1 [file metabolites-12-00891-s001.zip › Table S10.pdf]

Column 1	Variable	by Variable	Spearman ρ	Prob>|ρ|	q-value
1-methylhistidine	serum	plasma	0.95	<.0001	**0.0001**
1-methylhistidine	plasma	whole blood	0.9418	<.0001	**0.0001**
1-methylhistidine	serum	whole blood	0.9763	<.0001	**0.0001**
3-methylhistidine	serum	plasma	0.9725	<.0001	**0.0001**
3-methylhistidine	plasma	whole blood	0.9472	<.0001	**0.0001**
3-methylhistidine	serum	whole blood	0.9663	<.0001	**0.0001**
alanine	serum	plasma	0.984	<.0001	**0.0001**
alanine	plasma	whole blood	0.8932	<.0001	**0.0001**
alanine	serum	whole blood	0.9161	<.0001	**0.0001**
alpha-aminoadipic acid	serum	plasma	0.8728	<.0001	**0.0001**
alpha-aminoadipic acid	serum	whole blood	0.1333	0.4382	0.4458
alpha-aminoadipic acid	plasma	whole blood	0.1387	0.4197	0.4307
alpha-aminobutyric acid	serum	plasma	0.9913	<.0001	**0.0001**
alpha-aminobutyric acid	plasma	whole blood	0.9671	<.0001	**0.0001**
alpha-aminobutyric acid	serum	whole blood	0.9773	<.0001	**0.0001**
ammonia	serum	plasma	0.6649	<.0001	**0.0001**
ammonia	serum	whole blood	0.5115	0.0014	**0.0016**
ammonia	plasma	whole blood	0.5145	0.0013	**0.0016**
anserine	serum	plasma	0.6982	<.0001	**0.0001**
anserine	plasma	whole blood	0.5576	0.0004	**0.0005**
anserine	serum	whole blood	0.6108	<.0001	**0.0001**
arginine	serum	plasma	0.9166	<.0001	**0.0001**
arginine	serum	whole blood	0.7493	<.0001	**0.0001**
arginine	plasma	whole blood	0.8147	<.0001	**0.0001**
asparagine	serum	plasma	0.9575	<.0001	**0.0001**
asparagine	plasma	whole blood	0.7197	<.0001	**0.0001**
asparagine	serum	whole blood	0.7951	<.0001	**0.0001**
aspartic acid	serum	plasma	0.7256	<.0001	**0.0001**
aspartic acid	serum	whole blood	0.0479	0.7816	0.7816
aspartic acid	plasma	whole blood	0.1027	0.5511	0.5559
beta-alanine	serum	plasma	0.6063	<.0001	**0.0001**
beta-alanine	serum	whole blood	0.4946	0.0022	**0.0025**
beta-alanine	plasma	whole blood	0.5734	0.0003	**0.0004**
carnosine	serum	plasma	0.9356	<.0001	**0.0001**
carnosine	plasma	whole blood	0.7467	<.0001	**0.0001**
carnosine	serum	whole blood	0.7576	<.0001	**0.0001**
citrulline	serum	plasma	0.9931	<.0001	**0.0001**
citrulline	plasma	whole blood	0.9493	<.0001	**0.0001**
citrulline	serum	whole blood	0.9588	<.0001	**0.0001**
cystathionine	serum	plasma	0.9609	<.0001	**0.0001**
cystathionine	serum	whole blood	0.6708	<.0001	**0.0001**
cystathionine	plasma	whole blood	0.6914	<.0001	**0.0001**
cystine	serum	plasma	0.8708	<.0001	**0.0001**
cystine	serum	whole blood	0.2414	0.156	0.1659
cystine	plasma	whole blood	0.323	0.0546	0.0592
ethanolamine	serum	plasma	0.581	0.0002	**0.0003**

ethanolamine	serum	whole blood	0.4981	0.002	**0.0023**
ethanolamine	plasma	whole blood	0.5354	0.0008	**0.0010**
glutamic acid	serum	plasma	0.7508	<.0001	**0.0001**
glutamic acid	serum	whole blood	0.5138	0.0014	**0.0016**
glutamic acid	plasma	whole blood	0.5511	0.0005	**0.0006**
glutamine	serum	plasma	0.9884	<.0001	**0.0001**
glutamine	plasma	whole blood	0.949	<.0001	**0.0001**
glutamine	serum	whole blood	0.9503	<.0001	**0.0001**
glycine	serum	plasma	0.9884	<.0001	**0.0001**
glycine	serum	whole blood	0.9539	<.0001	**0.0001**
glycine	plasma	whole blood	0.9542	<.0001	**0.0001**
histidine	serum	plasma	0.9017	<.0001	**0.0001**
histidine	plasma	whole blood	0.8106	<.0001	**0.0001**
histidine	serum	whole blood	0.8175	<.0001	**0.0001**
homocystine	serum	plasma	0.9227	<.0001	**0.0001**
homocystine	serum	whole blood	0.4885	0.0025	**0.0028**
homocystine	plasma	whole blood	0.5028	0.0018	**0.0021**
hydroxylysine	serum	plasma	0.4691	0.0039	**0.0043**
hydroxylysine	serum	whole blood	-0.2376	0.1629	0.1717
hydroxylysine	plasma	whole blood	0.2227	0.1918	0.2004
hydroxyproline	serum	plasma	0.9284	<.0001	**0.0001**
hydroxyproline	plasma	whole blood	0.9315	<.0001	**0.0001**
hydroxyproline	serum	whole blood	0.9676	<.0001	**0.0001**
isoleucine	serum	plasma	0.9665	<.0001	**0.0001**
isoleucine	plasma	whole blood	0.894	<.0001	**0.0001**
isoleucine	serum	whole blood	0.895	<.0001	**0.0001**
leucine	serum	plasma	0.948	<.0001	**0.0001**
leucine	plasma	whole blood	0.8345	<.0001	**0.0001**
leucine	serum	whole blood	0.8546	<.0001	**0.0001**
lysine	serum	plasma	0.9925	<.0001	**0.0001**
lysine	plasma	whole blood	0.8059	<.0001	**0.0001**
lysine	serum	whole blood	0.8088	<.0001	**0.0001**
methionine	serum	plasma	0.955	<.0001	**0.0001**
methionine	plasma	whole blood	0.8206	<.0001	**0.0001**
methionine	serum	whole blood	0.8713	<.0001	**0.0001**
ornithine	serum	plasma	0.9793	<.0001	**0.0001**
ornithine	serum	whole blood	0.7663	<.0001	**0.0001**
ornithine	plasma	whole blood	0.7905	<.0001	**0.0001**
phenylalanine	serum	plasma	0.9423	<.0001	**0.0001**
phenylalanine	serum	whole blood	0.9048	<.0001	**0.0001**
phenylalanine	plasma	whole blood	0.9107	<.0001	**0.0001**
phosphoethanolamine	serum	plasma	0.5953	<.0001	**0.0001**
phosphoethanolamine	serum	whole blood	0.1843	0.282	0.2920
phosphoethanolamine	plasma	whole blood	0.257	0.1303	0.1399
phosphoserine	serum	plasma	0.4824	0.0029	**0.0032**
phosphoserine	serum	whole blood	0.3578	0.0321	**0.0351**
phosphoserine	plasma	whole blood	0.6052	<.0001	**0.0001**

proline	serum	plasma	0.9825	<.0001	**0.0001**
proline	plasma	whole blood	0.9483	<.0001	**0.0001**
proline	serum	whole blood	0.9529	<.0001	**0.0001**
serine	serum	plasma	0.984	<.0001	**0.0001**
serine	plasma	whole blood	0.9344	<.0001	**0.0001**
serine	serum	whole blood	0.9421	<.0001	**0.0001**
taurine	serum	plasma	0.5241	0.001	**0.0012**
taurine	plasma	whole blood	0.5586	0.0004	**0.0005**
taurine	serum	whole blood	0.6484	<.0001	**0.0001**
threonine	serum	plasma	0.9899	<.0001	**0.0001**
threonine	serum	whole blood	0.9491	<.0001	**0.0001**
threonine	plasma	whole blood	0.9622	<.0001	**0.0001**
tryptophan	serum	plasma	0.9905	<.0001	**0.0001**
tryptophan	plasma	whole blood	0.9176	<.0001	**0.0001**
tryptophan	serum	whole blood	0.9184	<.0001	**0.0001**
tyrosine	serum	plasma	0.9554	<.0001	**0.0001**
tyrosine	plasma	whole blood	0.7161	<.0001	**0.0001**
tyrosine	serum	whole blood	0.7249	<.0001	**0.0001**
urea	serum	plasma	0.9848	<.0001	**0.0001**
urea	plasma	whole blood	0.9356	<.0001	**0.0001**
urea	serum	whole blood	0.9514	<.0001	**0.0001**
valine	serum	plasma	0.9753	<.0001	**0.0001**
valine	plasma	whole blood	0.8293	<.0001	**0.0001**
valine	serum	whole blood	0.8564	<.0001	**0.0001**
