# Supplementary material for: Analytical Validation of an Assay for Concurrent Measurement of Amino Acids in Dog Serum and Comparison of Amino Acid Concentrations between Whole Blood, Plasma, and Serum from Dogs
Source: Metabolites. 2022 Sep 22;12(10):891. doi: 10.3390/metabo12100891 (PMC9608751; doi:10.3390/metabo12100891)
Supplement: Supplementary file 1 [file metabolites-12-00891-s001.zip › Table S3.pdf]

S3 Table. Intra-assay variability (precision). Concentrations and coefficient of variation for eight replicates run consecutively from each of eight dogs.

Compound	median [range] μM[a]	median [range] CV%[b]
phosphoserine	5 [4-12]	5.7 [1.7-22.0]
taurine	206 [107-510]	1.0 [0.2-1.5]
urea	6961 [2081-14595]	0.3 [0.2-2.4]
aspartic acid	10 [6-23]	2.0 [0.7-2.9]
threonine	163 [85-401]	0.2 [0.2-0.8]
serine	120 [100-153]	0.3 [0.2-0.8]
asparagine	60 [23-76]	3.1 [1.8-5.7]
glutamic acid	62 [23-194]	1.7 [0.5-2.5]
glutamine	579 [384-775]	0.4 [0.2-0.6]
α-aminoadipic acid	9 [6-13]	4.7 [2.3-24.1]
glycine	214 [151-253]	0.2 [0.1-0.3]
alanine	468 [202-622]	0.2 [0.1-0.3]
citrulline	57 [20-103]	0.6 [0.3-3.4]
α-aminobutyric acid	26 [11-43]	1.3 [0.5-2.6]
valine	190 [86-228]	0.4 [0.2-0.8]
methionine	50 [33-62]	1.4 [1.0-4.3]
cystathionine	9 [0-15]	8.8 [4.2-17.6]
isoleucine	63 [42-108]	0.7 [0.4-1.3]
leucine	127 [72-173]	0.3 [0.1-0.5]
tyrosine	43 [21-59]	1.3 [0.8-4.7]
phenylalanine	66 [44-105]	1.0 [0.7-1.5]
ammonia	68 [49-303]	2.7 [0.6-5.2]
ornithine	18 [10-46]	1.5 [0.7-3.6]
lysine	186 [92-259]	0.3 [0.2-1.0]
1-methylhistidine	14 [4-105]	5.7 [0.5-32.1]
histidine	83 [61-100]	1.1 [0.7-1.6]
tryptophan	55 [29-101]	2.8 [1.0-4.4]
3-methylhistidine	12 [8-44]	6.1 [1.6-17.8]
carnosine	27 [14-117]	4.1 [2.7-7.1]
arginine	150 [107-213]	0.5 [0.2-1.0]
proline	136 [75-154]	1.3 [0.8-2.1]

Repeatability and reproducibility of the assay. [a]Concentrations of samples used (the median and range of the median of eight replicates from eight different animals). [b]Coefficient of variation, calculated from eight replicates from each of eight animals. Compounds that were not detected in half or more of samples were excluded from analysis (phosphoethanolamine, sarcosine, cystine, β-alanine, β-aminoisobutyric acid, homocystine, γ-aminobutyric acid, ethanolamine, hydroxylysine, anserine, and hydroxyproline).
