# Supplementary material for: Analytical Validation of an Assay for Concurrent Measurement of Amino Acids in Dog Serum and Comparison of Amino Acid Concentrations between Whole Blood, Plasma, and Serum from Dogs
Source: Metabolites. 2022 Sep 22;12(10):891. doi: 10.3390/metabo12100891 (PMC9608751; doi:10.3390/metabo12100891)
Supplement: Supplementary file 1 [file metabolites-12-00891-s001.zip › Table S4.pdf]

S4 Table. Inter-assay variability (reproducibility). Concentrations and coefficient of variation for eight replicates run 1 to 14 days apart from each of eight dogs.

Compound	median [range] μM[a]	median [range] CV%[b]
phosphoserine	5 [4-13]	9.5 [3.3-32.3]
taurine	209 [110-505]	1.1 [0.8-1.7]
urea	7135 [2131-14482]	4.9 [3.9-6.0]
aspartic acid	10 [6-22]	4.2 [1.2-8.6]
threonine	164 [81-394]	1.3 [0.6-1.7]
serine	119 [100-153]	1.7 [0.8-2.1]
asparagine	63 [24-78]	4.2 [2.6-8.7]
glutamic acid	63 [24-195]	2.5 [1.3-3.9]
glutamine	628 [421-849]	3.3 [3.1-3.9]
α-aminoadipic acid	8 [3-13]	21.5 [12.6-37.3]
glycine	214 [151-252]	1.0 [0.5-1.4]
alanine	469 [200-618]	0.7 [0.5-1.5]
citrulline	57 [19-104]	2.1 [1.6-3.9]
α-aminobutyric acid	26 [10-43]	3.7 [1.7-6.7]
valine	192 [86-230]	0.9 [0.6-2.2]
methionine	51 [33-70]	3.2 [2.3-4.9]
cystathionine	9 [2-15]	9.7 [4.1-23.6]
isoleucine	64 [42-109]	1.0 [0.6-2.5]
leucine	127 [72-175]	0.8 [0.4-1.6]
tyrosine	44 [21-59]	1.9 [1.3-4.7]
phenylalanine	66 [45-105]	1.7 [1.2-2.3]
ammonia	81 [62-309]	3.9 [2.0-5.3]
hydroxylysine	12 [10-20]	10.1 [3.2-16.4]
ornithine	18 [10-46]	3.0 [1.2-5.4]
lysine	187 [92-260]	0.7 [0.4-2.1]
1-methylhistidine	14 [8-106]	7.0 [0.7-14.2]
histidine	83 [62-102]	1.7 [0.8-2.3]
tryptophan	56 [29-103]	3.4 [2.2-5.0]
3-methylhistidine	12 [9-44]	8.4 [4.3-12.1]
carnosine	29 [20-119]	7.4 [5.0-10.5]
arginine	150 [107-214]	2.1 [1.8-2.4]
proline	137 [74-152]	2.8 [1.7-5.4]

Repeatability and reproducibility of the assay. [a]Concentrations of samples used (the median and range of the median of eight replicates from eight different animals). [b]Coefficient of variation, calculated from eight replicates from each of eight animals. Compounds that were not detected in half or more of samples were excluded from analysis (phosphoethanolamine, sarcosine, cystine, β-alanine, β-aminoisobutyric acid, homocystine, γ-aminobutyric acid, ethanolamine, anserine, and hydroxyproline).
